# A CUC1/auxin genetic module links cell polarity to patterned tissue growth and leaf shape diversity in crucifer plants

**DOI:** 10.1073/pnas.2321877121

**Published:** 2024-06-21

**Authors:** Zi-Liang Hu, David Wilson-Sánchez, Neha Bhatia, Madlen I. Rast-Somssich, Anhui Wu, Daniela Vlad, Liam McGuire, Lachezar A. Nikolov, Patrick Laufs, Xiangchao Gan, Stefan Laurent, Adam Runions, Miltos Tsiantis

**Affiliations:** ^a^Department of Comparative Development and Genetics, Max Planck Institute for Plant Breeding Research, Cologne 50829, Germany; ^b^Université Paris-Saclay, Institut national de recherche pour l’agriculture, l’alimentation et l’environnement, AgroParisTech, Institut Jean-Pierre Bourgin, Versailles 78000, France; ^c^Department of Computer Science, University of Calgary, Calgary, AB T2N 1N4, Canada

**Keywords:** auxin, *CUP-SHAPED COTYLEDON* genes, complex leaves, evolution of development (evo-devo), leaf development

## Abstract

How tissue-level information encoded by fields of regulatory gene activity is translated into the patterns of cell polarity and growth that generate the diverse shapes of different species remains poorly understood. Here, we investigate this problem in the case of leaf shape differences between *Arabidopsis thaliana*, which has simple leaves, and its relative *Cardamine hirsuta* that has complex leaves divided into leaflets. We show that patterned expression of the transcription factor CUP-SHAPED COTYLEDON1 in *C. hirsuta* (ChCUC1) is a key determinant of leaf shape differences between the two species. Through inducible genetic perturbations, time-lapse imaging of growth, and computational modeling, we find that ChCUC1 provides instructive input into auxin-based leaf margin patterning. This input arises via transcriptional regulation of multiple auxin homeostasis components, including direct activation of WAG kinases that are known to regulate the polarity of PIN-FORMED auxin transporters. Thus, we have uncovered a mechanism that bridges biological scales by linking spatially distributed and species-specific transcription factor expression to cell-level polarity and growth, to shape diverse leaf forms.

## Significance

How spatially distributed gene activities are translated into the patterns of cell polarity and growth that generate the diverse forms of multicellular eukaryotes remains poorly understood. Here, we show that species-specific expression of the transcription factor CUP-SHAPED COTYLEDON1 (CUC1) is a key determinant of leaf-shape differences between two related plant species. By combining time-lapse imaging, genetics, and modeling, we found that CUC1 acts as a polarity switch. This switch regulates leaf shape through transcriptional activation of kinases that influence the polarity of auxin transporters, which pattern leaf growth through feedback with the hormone auxin. Thus, we have uncovered a mechanism that bridges biological scales by linking species-specific transcription factor expression to cell-level polarity and growth, to shape diverse leaf forms.

A key question in biology is how tissue-wide coordination of cell polarity and growth shapes organ geometry (1–7), and how such regulation is modified during evolution to generate morphological diversity (8–12). In plants, PIN-FORMED (PIN) proteins are central to organ development because they transport auxin polarly to create auxin asymmetries that underlie tissue patterning and growth (13–16). In plant shoots, PIN1 and auxin can engage in a positive feedback loop where auxin directs PIN1 polarity toward cells with high auxin response, thus forming polarity convergences and auxin maxima. In turn, PIN1 polarity reverses at the vicinity of these auxin maxima, which iteratively triggers new convergences. Such self-organizing patterns are believed to underlie phyllotaxis, the periodic organ initiation at the pluripotent shoot apical meristem (SAM), as well as the sequential formation of outgrowths at the margins of leaves (17–20).

A major open question in the field is to understand which upstream determinants regulate these PIN1 polarity patterns, and how they function in space and time. One hypothesis is that mechanical stresses produced by growth influence PIN1 polarity (21, 22). Although such mechanical models are elegant, their underlying molecular mechanisms remain enigmatic (2, 4, 23). Another possibility, that is not mutually exclusive, is that biochemical inputs act as direct cues for PIN1 polarity. For instance, AUXIN RESPONSE FACTOR 5/MONOPTEROS (ARF5/MP) has been shown to regulate the direction of PIN1 polarity during phyllotaxis (17); however, the mechanisms through which it modulates PIN1 polarity are unknown. PINOID (PID) family kinases can phosphorylate PINs and regulate their polarity and auxin efflux activity (24–27); however, there is little information on how their gene expression is regulated. Thus, tissue scale regulatory mechanisms underlying PIN1 polarity reversals in the vicinity of polarity convergences to shape organ growth remain unclear, as do mechanisms that provide species-specific inputs into this process.

**Fig. 1.**
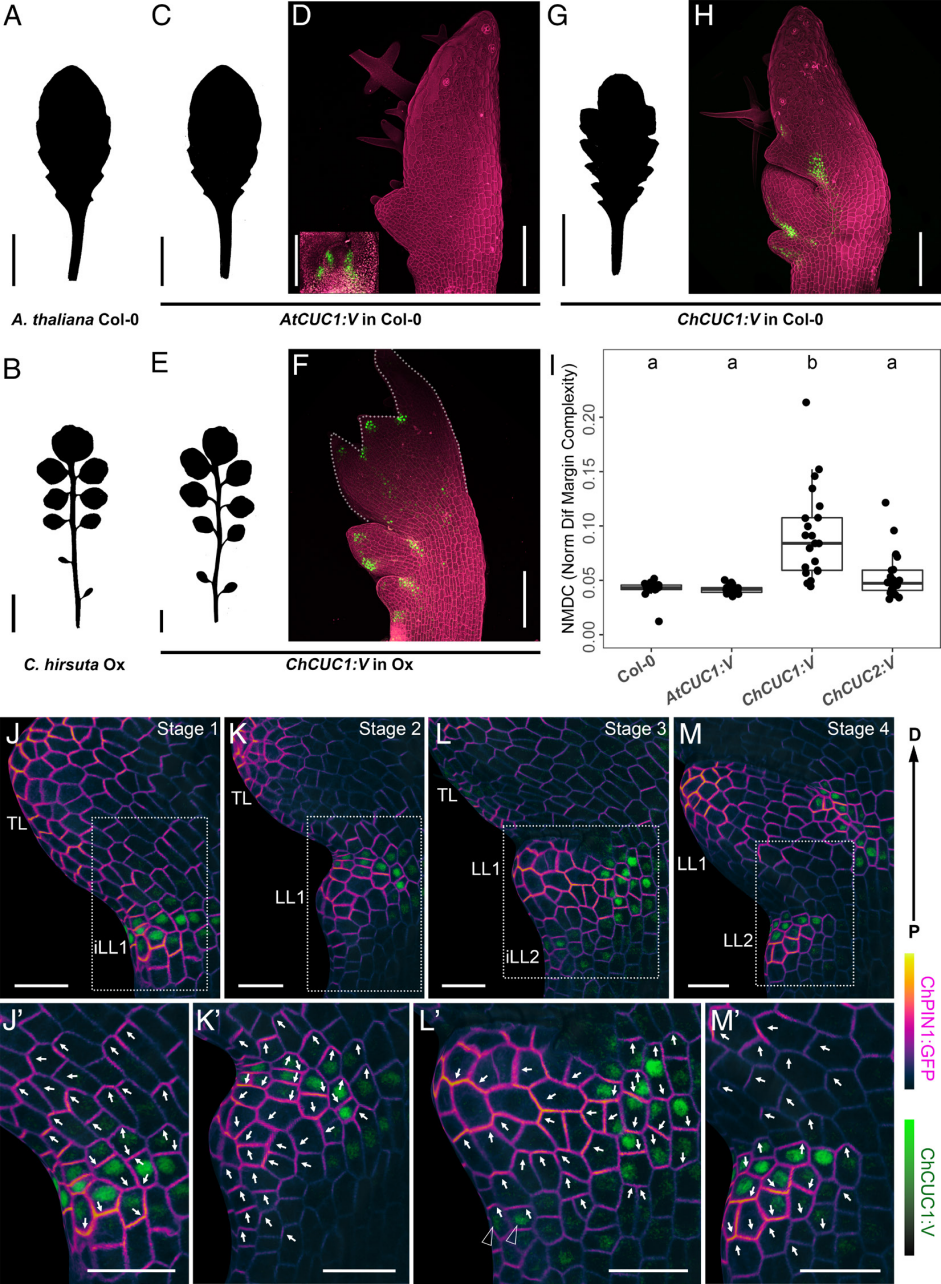
ChCUC1 is sufficient to increase *A. thaliana* leaf complexity upon interspecific gene transfer and its expression associates with PIN1 polarity reversals. (*A* and *B*) Silhouettes of rosette leaf 8 from wild-type *A. thaliana* (Columbia-0, Col-0) (*A*) and *C. hirsuta* (Oxford; Ox) (*B*). (*C* and *D*) Silhouettes (*C*) and transgene expression (*D*) in *A. thaliana* rosette leaf 8 carrying *AtCUC1:V* (*AtCUC1p::AtCUC1g:Venus*). The *Inset* in (*D*) shows the vegetative shoot apex. (*E* and *F*) Silhouettes (*E*) and transgene expression (*F*) in *C. hirsuta* rosette leaf 8 carrying *ChCUC1:V* (*ChCUC1p::ChCUC1g:Venus*). (*G* and *H*) Silhouettes (*G*) and transgene expression (*H*) in *A. thaliana* rosette leaf 8 carrying *ChCUC1:V* (*ChCUC1p::ChCUC1g:V*). (*D*, *F*, and *H*) Maximum intensity projections of confocal stacks. Green: Venus signal; magenta: cell walls visualized with propidium iodide (PI). (*I*) Leaf complexity of leaf 8 from the indicated genotypes by measurement of Normalized Difference Margin Complexity [(perimeter contour-perimeter convex hull)/(perimeter contour + perimeter convex hull)]. Letters a and b indicate significant differences by the Kruskal–Wallis test with Dunn’s post hoc test (*α* = 0.05). *ChCUC2:V* refers to *ChCUC2p::ChCUC2g:V* (34). (*C*–*I*) Replication: *n* (phenotypic analysis) ≥ 20 transgenic T1 lines, *n* (confocal microscopy) = 3 lines. (*J*–*M*’) *C. hirsuta* leaf 5 at different developmental stages showing epidermal expression of *ChCUC1:V* (*ChCUC1p::ChCUC1g:V*) and *ChPIN1:GFP* (*ChPIN1p::ChPIN1g:eGFP*) projected onto a MorphographX mesh (*SI Appendix*, *Materials and Methods*). The white arrows on the leaf margin cells indicate the direction of ChPIN1:GFP polarity. The black arrowheads in (*L*’) indicate leaf margin cells with *ChCUC1:V* expression and apical ChPIN1:GFP polarity. The dotted *Insets* indicate the areas magnified in (*J*’–*M*’). D, distal; P, proximal; TL, terminal leaflet; LL, lateral leaflet; iLL, initiating lateral leaflet. *n* = 3 leaves per stage. The leaf silhouettes were obtained 21 d after sowing. [Scale bars: 1 cm (*A*–*C*, *E*, and *G*); 100 µm (*D*, *F*, and *H*); 20 µm (*J*–*M*’).]

The study of CUP-SHAPED COTYLEDON (CUC) transcription factors may offer mechanistic insight into how upstream developmental cues influence polarity relevant to growth, as CUCs, through unknown mechanisms, can influence PIN1-dependent positioning and orientation of new growth axes in different organs, as well as repress growth after auxin activity maxima establishment (9, 18, 28–31). Leaf shape differences between *Arabidopsis thaliana* and its close relative *Cardamine hirsuta* offer an attractive system to understand how this process is tuned during evolution. This is because in these species, a conserved CUC-PIN1-auxin patterning module sequentially distributes auxin maxima along the leaf margin (9), which then translates into divergent morphologies: simple leaves in *A. thaliana* with small marginal protrusions called serrations (Fig. 1*A*) and complex leaves in *C. hirsuta* with distinct marginal protrusions called leaflets (Fig. 1*B*). *C. hirsuta* leaves express three *CUC* paralogues (*ChCUC1*, *ChCUC2,* and *ChCUC3*), which are redundantly required for leaflet formation, whereas in *A. thaliana CUC1* transcripts are excluded from the leaves (Fig. 1 *C*–*F*) (32, 33). This raises the possibility that *CUC1* expression provides species-specific input into leaf marginal patterning and contributes to leaf shape variation between *C. hirsuta* and *A. thaliana* by influencing cell polarity and growth in developing leaf primordia.

Here, we investigated the mechanisms underlying ChCUC1-dependent PIN1 polarization and their role in the diversification of crucifer leaf form. We demonstrated that interspecies gene transfer of *ChCUC1* into *A. thaliana* is sufficient to drive gene expression in leaves and to transform the simple leaves of *A. thaliana* into more complex ones that resemble those of *C. hirsuta.* By combining inducible perturbations, including the generation of genetic mosaics, with time-lapse imaging and computation of cellular growth patterns, we found that ChCUC1 affects development by providing instructive input into PIN1-dependent auxin patterning. Using RNA-seq, ChIP-seq, and DAP-seq, we show that ChCUC1 regulates the expression of auxin-related genes, including the direct transcriptional activation of WAG kinases, which are known regulators of PIN1 polarity. We then used genome editing to genetically validate the role of these kinases in *C. hirsuta* leaflet formation. Finally, we utilized computational modeling to conceptualize how the CUC1/WAG module affects tissue-level auxin-dependent patterning. Taken together, our data demonstrate how a CUC1/WAG transcriptional module regulates cell polarity, and how its action is translated into species-specific organ morphology via the modulation of cellular growth.

## Results and Discussion

### CUC1 Is a Key Determinant of Leaf Shape Differences between *A. thaliana* and *C. hirsuta*.

To test the idea that species-specific *CUC1* expression contributes to differences in leaf growth and shape of *C. hirsuta* versus *A. thaliana*, we first conducted an interspecies gene transfer experiment (35) and evaluated the ability of *C. hirsuta* versus *A. thaliana CUC1*, expressed under the control of their endogenous upstream sequences (*ChCUC1p::ChCUC1g:Venus*, [*ChCUC1:V*]; *AtCUC1p::AtCUC1g:V*, [*AtCUC1:V*]), to increase leaf complexity in *A. thaliana*. We observed that *ChCUC1:V* but not *AtCUC1:V* can drive expression in *A. thaliana* leaves and increase their complexity (Fig. 1 *C*, *D*, *G*, and *H*). We also found that leaf complexity in *A. thaliana* increased significantly by expressing *ChCUC1:V* compared to *ChCUC2:V* (*ChCUC2p::ChCUC2g:V*) (Fig. 1*I*). Based on these findings and the observation that *ChCUC1* can also restore PIN1:GFP convergences and serrations in the smooth leaf margin of the *A. thaliana cuc2-1* mutant (*SI Appendix*, Fig. S1 *A–F*’), we conclude that species-specific expression of *ChCUC1* in leaves plays a central role in regulating *C. hirsuta* leaf patterning and leaf shape diversity in crucifer plants.

On the basis of the above findings, we leveraged *ChCUC1* to understand the effects of *CUCs* on PIN1-dependent leaf margin patterning and leaf shape. First, we visualized the expression of *ChCUC1:V* during the initiation of lateral leaflets in *C. hirsuta* complex leaves, while simultaneously monitoring PIN1 polarity using a functional ChPIN1:GFP fusion protein constructed for the purpose of this study (*ChPIN1p::ChPIN1g:eGFP*; *SI Appendix*, Fig. S16). We observed *ChCUC1:V* expression at lateral leaflet (LL) initiation sites along the leaf margin. At the LL1 initiation site, ChPIN1:GFP polarities were oriented basally, away from a preexisting protrusion of the terminal leaflet (TL), which displayed apical polarity (Fig. 1 *J* and *J’* and *SI Appendix*, Fig. S18*A*). ChPIN1:GFP later formed a polarity convergence at the LL1 site, marking its protrusion, while *ChCUC1:V* expression was limited to the distal side of the LL1 base (Fig. 1 *K* and *K’* and *SI Appendix*, Fig. S18*B*). As LL1 developed further, *ChCUC1:V* expression was faintly detected at the proximal side of the LL1 base (Fig. 1 *L* and *L’*). ChPIN1:GFP polarities in these *ChCUC1:V*-expressing cells were still oriented distally, toward the tip of the LL1 (Fig. 1 *L* and *L’*, black arrowheads; *SI Appendix*, Fig. S18*C*), but later reversed and formed a new polarity convergence (Fig. 1 *M* and *M’* and *SI Appendix*, Fig. S18*D*). This polarity convergence correlated with the emergence of the LL2 protrusion, marked by *ChCUC1:V* expression at its distal side (Fig. 1 *M* and *M’*). These findings indicate that during leaflet initiation, *ChCUC1* expression correlates with the reversal of ChPIN1:GFP polarity at the base of existing LLs, toward a subsequent LL initiation site. Fate mapping of the cells expressing *ChCUC1:V* before leaflet emergence showed that PIN1 convergences are derived from the proximal ChCUC1 domain where PIN1 reversals occur and that they additionally recruit more proximal non *ChCUC1:V*-expressing cells (*SI Appendix*, Fig. S2). Together, these observations are consistent with the idea that *ChCUC1* acts upstream of ChPIN1.

### ChCUC1 Provides Instructive Input into Auxin-Based Margin Patterning.

**Fig. 2.**
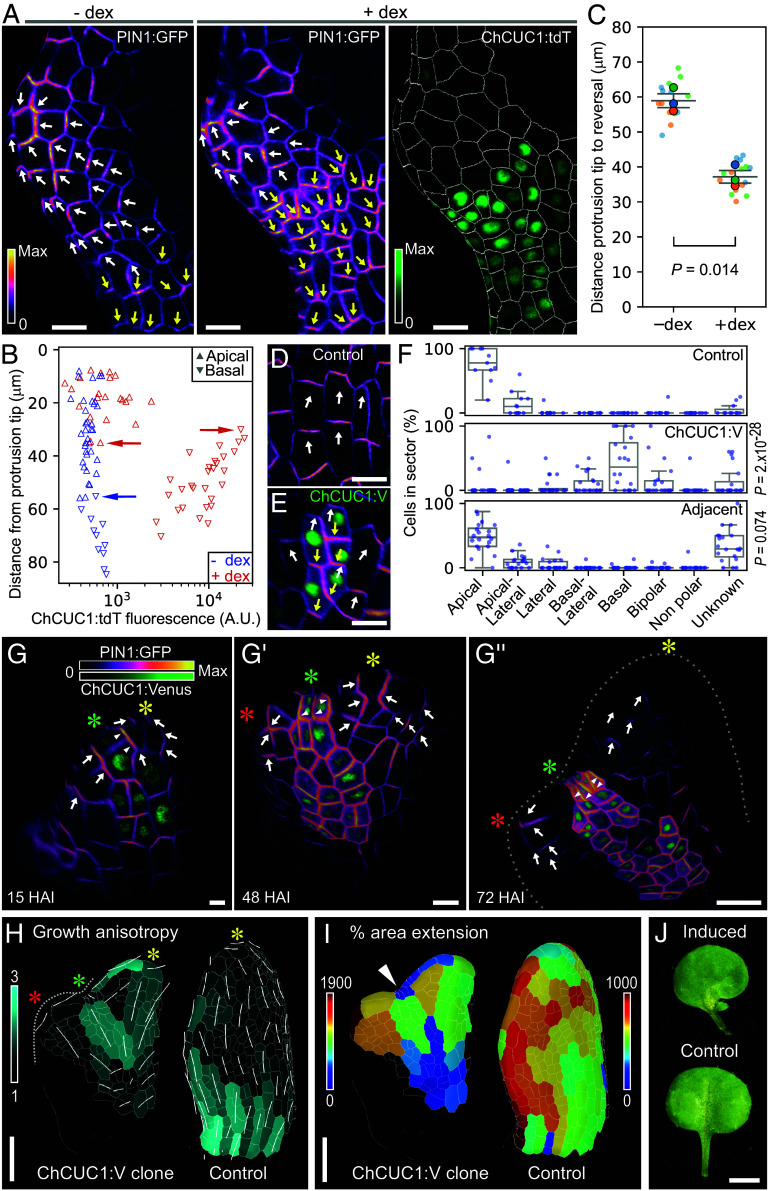
ChCUC1 is sufficient to repolarize PIN1 and organize new outgrowths in the leaf margin. (*A*–*C*) Effect of ChCUC1 induction on PIN1:GFP polarity during *A. thaliana* leaf margin patterning. (*A*) Leaf 4 from *ChCUC1p::LhG4:GR; Op6::ChCUC1:tdTomato* plants showing PIN1:GFP polarity and ChCUC1:tdT expression 24 h after treatment with dexamethasone (+dex) or mock (−dex) solutions. The white and yellow arrows on the leaf margin cells indicate, respectively, PIN1:GFP polarization toward a protrusion tip, or toward the opposite direction. (*B*) Analysis of the cells in (*A*), showing their PIN1:GFP polarity, ChCUC1:tdT expression, and distance from the protrusion tip. The horizontal arrows highlight that PIN1 reversal (downward-pointing triangles) after dex treatment can be explained by the level of ChCUC1:tdT expression, which overrides the polarity pattern of the control sample. (*C*) Distance from the tip of a protrusion to PIN1:GFP polarity reversal in leaves treated with dex and mock solutions. The reversal position in each leaf (large dots) was calculated by averaging the distances of the five cells closest to the protrusion with clear basal polarity (small dots). *n* = 3 leaves per treatment. Unpaired *t* test. (*D*–*F*) PIN1:GFP polarity in response to ectopic ChCUC1:V clones in the abaxial epidermis of leaves and sepals of *A. thaliana HSp::dBox:Cre; 35Sp::lox--lox::ChCUC1:V*. (*D* and *E*) Examples of control cells (*D*) and ChCUC1:V clones (*E*). The white and yellow arrows indicate, respectively, apical and basal PIN1:GFP accumulation. (*F*) Frequency of PIN1:GFP polarity directions in control cells, ChCUC1 clones, and neighbors adjacent to ChCUC1:V clones, 24 h after heat shock. *n* (control) = 11 samples, *n* (ChCUC1:V clone) = 20. Chi-squared test, *P* values correspond to comparisons with the control group. (*G*–*J*) Effect of leaf margin ChCUC1:V clones on tissue polarity and growth. (*G*–*G*’’) PIN1:GFP polarity and ChCUC1:V clone expression in an *A. thaliana* leaf 1 over 72 h after heat shock induction (HAI). The white arrows on the leaf margin cells indicate the direction of PIN1:GFP accumulation. The yellow asterisk indicates the primary polarity convergence at the apex of the leaf. The green asterisk marks the margin cell lineage containing a ChCUC1:V clone. The red asterisk indicates an ectopic polarity convergence point. Frequency of outgrowths: margin ChCUC1:V clones = 3/3, controls = 0/3. (*H* and *I*) Growth anisotropy (*H*) and area extension (*I*) of the leaf shown in *G*–*G*’’ (over 57 h) and a control sample (over 48 h). The white lines in (*H*) indicate the main direction and magnitude of growth. The asterisks are as in (*G*–*G*’’). The white arrowhead in (*I*) points at a strong growth repression zone in the margin not observed in the control *A. thaliana* leaf 1. (*J*) Final morphology of a control leaf and a leaf subjected to heat shock that shows a margin sinus and an outgrowth. Leaves shown belong to nodes 1 or 2 14 d after sowing. *n* (induced) = 4, *n* (control) = 10. (*A* and *G*–*I*) MorphographX surface meshes with epidermal signal projected. [Scale bars: 10 µm (*A*, *D*, and *E*); 10 µm (*G*–*G*’’); 30 µm (*H* and *I*); 20 mm (*J*).]

To determine whether *ChCUC1* is sufficient to cause PIN1 polarity reversals in the leaf margin, we expressed in *A. thaliana* a dexamethasone (dex)-inducible ChCUC1:tdTomato functional fusion from its endogenous upstream regulatory sequences (*ChCUC1p::LhG4:GR; Op6::ChCUC1:tdT; PIN1p::PIN1:GFP*; *SI Appendix*, Fig. S3). Then, we applied dex or mock solutions to developing leaves and characterized the PIN1:GFP polarity response 24 h later. The leaves from both treatments displayed convergent PIN1:GFP polarity fields directed toward the tips of margin protrusions (Fig. 2*A*, white arrows; *SI Appendix*, Fig. S18 *E* and *F*). However, while in mock samples PIN1:GFP polarity reversed at the base of the protrusion, dex-treated samples displayed polarity reversals in the protrusion itself (yellow arrows), and this behavior coincided spatially with *ChCUC1:tdT* expression (Fig. 2 *A* and *B* and *SI Appendix*, Fig. S4). As a consequence, *ChCUC1:tdT* expression caused a reduction in the distance between protrusion tips and the cells with reversed PIN1:GFP polarity in the basally adjacent margin (Fig. 2*C*). Therefore, *ChCUC1* margin expression can act as an instructive signal for PIN1 repolarization. To test whether *ChCUC1* can influence the frequency of leaf margin patterning events, we used the auxin activity reporter *DR5v2::NLS:tdT* to measure the number of expression foci in developing leaves of *A. thaliana* wild type versus *ChCUC1:V*, and observed that leaves expressing *ChCUC1:V* had more auxin maxima throughout early stages of development (*SI Appendix*, Fig. S5). Together, these findings indicate that ChCUC1 is sufficient to repolarize PIN1 during *A. thaliana* leaf margin development and to modulate the frequency of auxin patterning.

To test whether and how *ChCUC1* can alter PIN1 polarity directions to create new polarity convergences and growth axes, we generated genetic mosaics using heat shock-inducible Cre-lox recombination [*HSp::dBox:Cre*; *35Sp::lox-spacer-lox::ChCUC1:V* (36)] in *A. thaliana* plants expressing *PIN1p::PIN1:GFP*. We induced ChCUC1:V clones in leaves and sepals [modified leaves with a single polarity axis (37)] and monitored the PIN1:GFP polarity response for up to 72 h after heat shock induction (HAI). We found that in the abaxial epidermis, control cells that did not express *ChCUC1:V* displayed either apical or apical-lateral PIN1:GFP localization (Fig. 2 *D* and *F* and *SI Appendix*, Fig. S18 *G* and *H*). By contrast, in *ChCUC1:V*-expressing clones, the majority of cells showed basal, basal-lateral, or bipolar PIN1:GFP localization (Fig. 2 *E* and *F* and *SI Appendix*, Fig. S6). Imaging of ChCUC1:V clones at higher temporal resolution revealed that these cells, which were initially polarized apically, start repolarization 18 ± 3.5 HAI (n = 6; *SI Appendix*, Fig. S7). The polarity behaviors of direct neighbors of *ChCUC1:V*-expressing cells were comparable to control cells (Fig. 2 *E* and *F*), suggesting a cell-autonomous effect. These observations indicate that, although ChCUC1:V can disrupt the proximodistal PIN1 polarity field by reorienting PIN1:GFP cell autonomously, it is not sufficient to create a new polarity convergence in the abaxial epidermis. Therefore, we hypothesized that polarity convergences and outgrowths might be a specific feature of ChCUC1 action in the leaf margin. To test this idea, we used time-lapse imaging to follow the development of ChCUC1:V clones generated in the margins of the first *A. thaliana* leaf primordium. At 15 HAI, PIN1:GFP was symmetrically oriented toward the distal tip of the primordium in most marginal cells (Fig. 2*G*, yellow asterisk; *SI Appendix*, Fig. S8*A* and S18*I*), including those expressing *ChCUC1:V* (green asterisk). However, within 48 HAI, PIN1:GFP in ChCUC1:V cells transitioned to a bipolar orientation (Fig. 2 *G*’ and *G*’’, green asterisk; *SI Appendix*, Fig. S8*A*), indicating a disruption in marginal polarity. Additionally, cells proximal to the ChCUC1:V clone showed a PIN1:GFP reversal away from the clone and formed an ectopic polarity convergence, revealing a non-cell-autonomous response (Fig. 2 *G*’ and *G*’’, red asterisk; *SI Appendix*, Fig. S8*A*) associated with the emergence of an ectopic protrusion 72 HAI (Fig. 2*G’’* and *SI Appendix*, Fig. S8*A*). These appeared close to the distal tip of the leaf, where serrations do not normally emerge (9, 18), indicating that CUC1 was sufficient to trigger them. Additionally, we observed that ChCUC1:V clones induced changes in the direction of growth anisotropy in the adjacent margin cells (Fig. 2*H* and *SI Appendix*, Fig. S8*B*). We also observed localized growth repression in marginal cells expressing *ChCUC1:V* (Fig. 2*I* and *SI Appendix*, Fig. S8*C*), consistent with the local growth-repressing role of *CUC* genes (9, 38). As control we used cells in equivalent positions from heat-shocked first leaf primordia expressing PIN1:GFP and imaged up to 72 HAI. In those cells, PIN:GFP polarized toward the leaf tips (*SI Appendix*, Fig. S9*A*) and growth was aligned to the proximodistal axis (*SI Appendix*, Fig. S9 *B* and *C*). When heat-shocked Cre-lox-driven ChCUC1:V leaves were allowed to fully expand, they displayed changes in margin morphology, including the presence of lobes (Fig. 2*J*). Together, our analyses of localized *ChCUC1* expression under the control of *ChCUC1* regulatory sequences and genetic mosaics provide evidence for multiple roles of *ChCUC1*: 1) as a cell-autonomous promoter of PIN1 polarity reversal, 2) a local growth repressor, and 3) a long-range, non-cell-autonomous organizer of PIN1 polarity convergences in the leaf margin.

### Genetic Basis for ChCUC1 Action on Auxin Patterning.

**Fig. 3.**
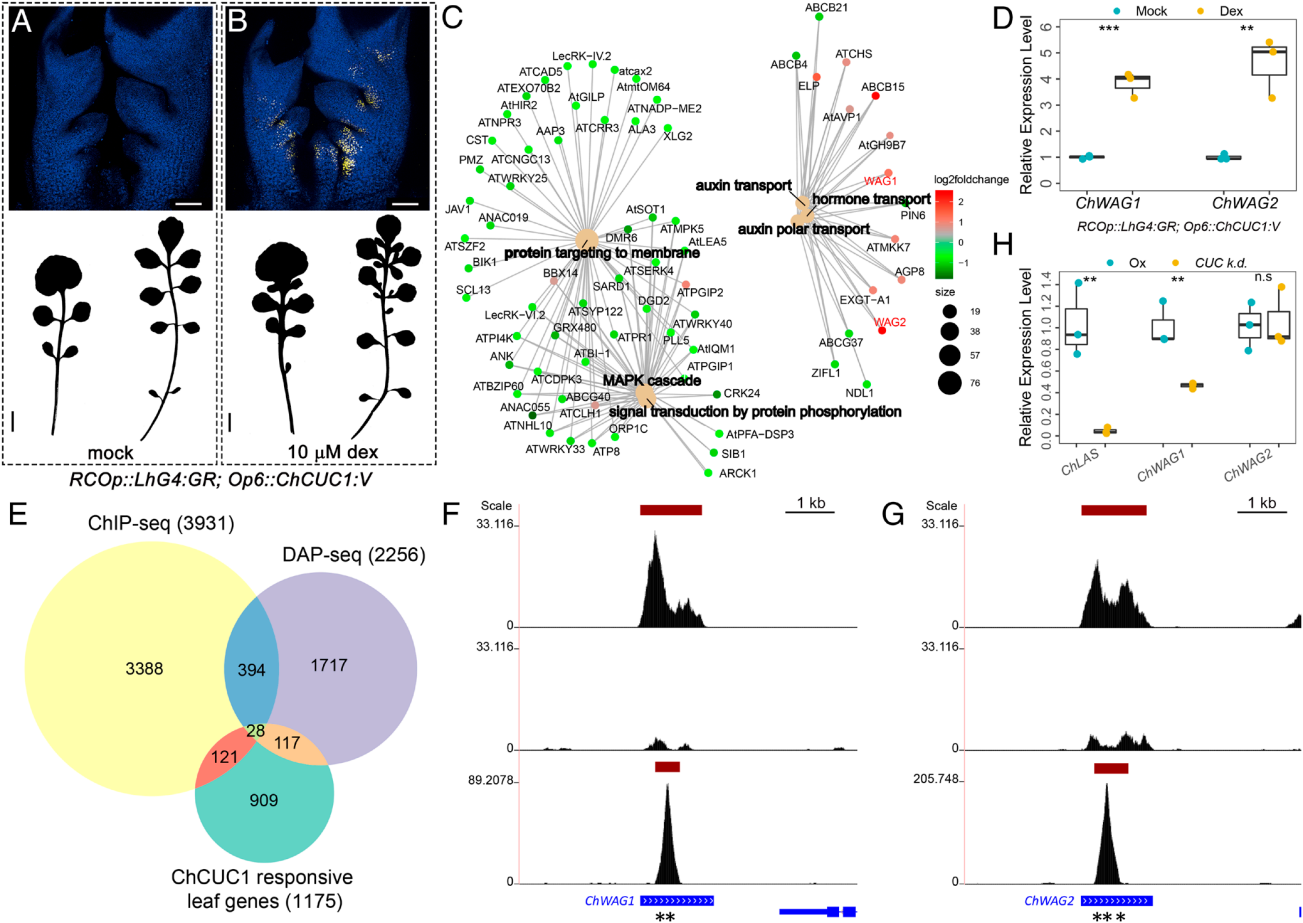
Identification of *ChCUC1* target genes through transcriptome analysis, ChIP-seq and DAP-seq. (*A* and *B*) *ChCUC1:V* expression (*Top*) and silhouettes (*Bottom*) of *C. hirsuta* rosette leaves 5 and 8 carrying *RCOp::LhG4:GR;Op6::ChCUC1:V* 2 h after mock (*Left*) and dexamethasone (dex, *Right*) treatment. *Top* panels, maximum intensity projection of confocal stacks. Yellow: Venus signal; blue: chlorophyll autofluorescence. *n* = 5 transgenic lines. [Scale bars: 100 µm (*Upper* panels); 1 cm (*Lower* panels).] (*C*) Gene-concept network for selected biological processes depicting linkages between significantly enriched GO terms and the associated genes detected after 8 h of *ChCUC1:V* induction. Each node represents a gene and is color-coded according to its expression fold change. (*D*) Relative transcript abundance of *ChWAG1* and *ChWAG2* 8 h after *ChCUC1:V* induction. qRT-PCR performed on *C. hirsuta RCOp::LhG4:GR;Op6::ChCUC1:V* shoot apices with developing leaves 12 d after sowing. Asterisks indicate statistically significant differences (Unpaired *t* test, ***P < 0.001, **P < 0.01, *P < 0.05). (*E*) Proportional Venn diagram depicting the number of *ChCUC1* responsive leaf genes, direct *ChCUC1* target genes detected by ChIP-seq, direct *ChCUC1* target genes detected by DAP-seq, and the overlaps between datasets. Significant enrichment between ChIP-seq and DAP-seq datasets was found by Fisher’s exact test (*P* < 5.64 × 10^−14^), as well as between direct ChCUC1 target genes detected by DAP-seq and *ChCUC1* responsive leaf genes datasets (*P* < 6.67 × 10^−09^), and between direct ChCUC1-target genes detected by ChIP-seq and up-regulated genes in response to ChCUC1 induction (*P* < 1.85 × 10^−06^). (*F* and *G*) ChIP-seq and DAP-seq binding profiles at the *ChWAG1* and *ChWAG2* loci. From top to bottom: ChCUC1 MOBE-ChIP-seq using *C. hirsuta ChCUC1:V* transgenic plants, control MOBE-ChIP-seq using *C. hirsuta* Ox, and ChCUC1 DAP-seq. Pooled signal from three biological replicates from ChIP-seq or DAP-seq assays are shown. Vertical axes: −log(*P*-value); red bars: significant peaks; blue bars: exons; asterisks: CUC binding sites. (*H*) Relative transcript abundance of *ChWAG1* and *ChWAG2* in *C. hirsuta* wild type and *ChCUC knock-down* (*CUC k.d.*, *35Sp::MIR164A;35Sp::CUC3-RNAi*) measured by qRT-PCR on developing leaves (300 to 500 µm in length, 12 d after sowing). *LATERAL SUPPRESSOR* (*LAS*), a previously described CUC downstream gene (42) was used as a positive control. Same statistical analysis as in (*D*).

To understand the molecular mechanisms through which *ChCUC1* exerts these effects on auxin patterning and leaf development, we sought to identify its downstream target genes. To this end, we transformed *C. hirsuta* wild type with a dex-inducible transgene (*RCOp::LhG4:GR; Op6::ChCUC1:V*), in which *ChCUC1* expression is driven by the *REDUCED COMPLEXITY* (*RCO*) promoter that acts in discrete foci exclusively along the margin of developing complex leaves (39). In transformants, we observed *ChCUC1:V* expression 2 h after dex induction (HAI), and the subsequent emergence of ectopic LLs during development (Fig. 3 *A* and *B*), indicating that localized *ChCUC1:V* activation is sufficient to generate LLs. Therefore, this transgenic line provides good opportunity to identify ChCUC1 transcriptional targets involved in lateral leaflet formation while reducing confounding effects from other cell types where *ChCUC1* is expressed, such as the SAM (32). We performed transcriptomic analysis on 12-d-old shoot apices comprising developing leaves (sampled 2, 4, 6, and 8 HAI), and identified *ChCUC1:V*-dependent differentially expressed genes (DEGs; fold change > 1.5 and adjusted *P*-value < 0.05) at different time points (*SI Appendix*, Fig. S10*A* and Dataset S1). Among the DEGs induced 8 HAI (*SI Appendix*, Fig. S10*B*), the Gene Ontology (GO) terms “auxin polar transport,” “protein targeting to membrane,” “MAPK cascade,” and “signal transduction by protein phosphorylation” were significantly enriched (Fig. 3*C* and *SI Appendix*, Fig. S10 *C–F*) indicating that *ChCUC1* may act through these processes. We found 1,175 genes showing differential expression at more than one time point, and refer to these as *ChCUC1* responsive leaf genes (Dataset S1). Notably, these genes included *WAVY ROOT GROWTH 1* and *2* (*ChWAG1* and *ChWAG2*) (Fig. 3 *C* and *D*), which encode AGCVIII kinases that phosphorylate PINs and modulate their polarity and auxin efflux activity (25, 27). Three lines of evidence indicate that *ChWAG* genes are direct transcriptional targets of *ChCUC1* during leaf development. First, ChCUC1 associates with chromatin at the *ChWAG1* and *ChWAG2* loci in ChIP-seq and DAP-seq assays (Fig. 3 *E–G* and *SI Appendix*, Fig. S11 and Dataset S2). The C[T/G]TG binding sites identified in these experiments correspond to the known core DNA binding motif of the NAC superfamily of proteins to which ChCUC1 belongs (40, 41) and are present in *ChWAG* genes (Fig. 3 *F* and *G* and *SI Appendix*, Fig. S11 *A* and *B*). Second, the intersection of *ChCUC1* responsive leaf genes, ChIP-seq, and DAP-seq datasets indicates that the number of overlapping genes is overrepresented relative to what would be expected by chance (Fig. 3*E* and Dataset S3). This analysis also allowed us to identify 28 high-confidence *ChCUC1* target genes that include *ChWAG1* and *ChWAG2*. Third, qRT-PCR analysis in *C. hirsuta* wild-type and *ChCUC knock-down* leaf primordia [which have reduced expression levels of *ChCUC1, 2,* and *3* (32)] indicated that *ChCUC1*, together with its redundantly acting paralogues *ChCUC2* and *ChCUC3*, are required for *ChWAG1* transcripts to accumulate in the developing leaf primordia (Fig. 3*H*). Taken together, these assays indicate that direct transcriptional induction of *ChWAG* kinases may mediate *ChCUC1* effects on ChPIN1 function during *C. hirsuta* leaf margin patterning.

**Fig. 4.**
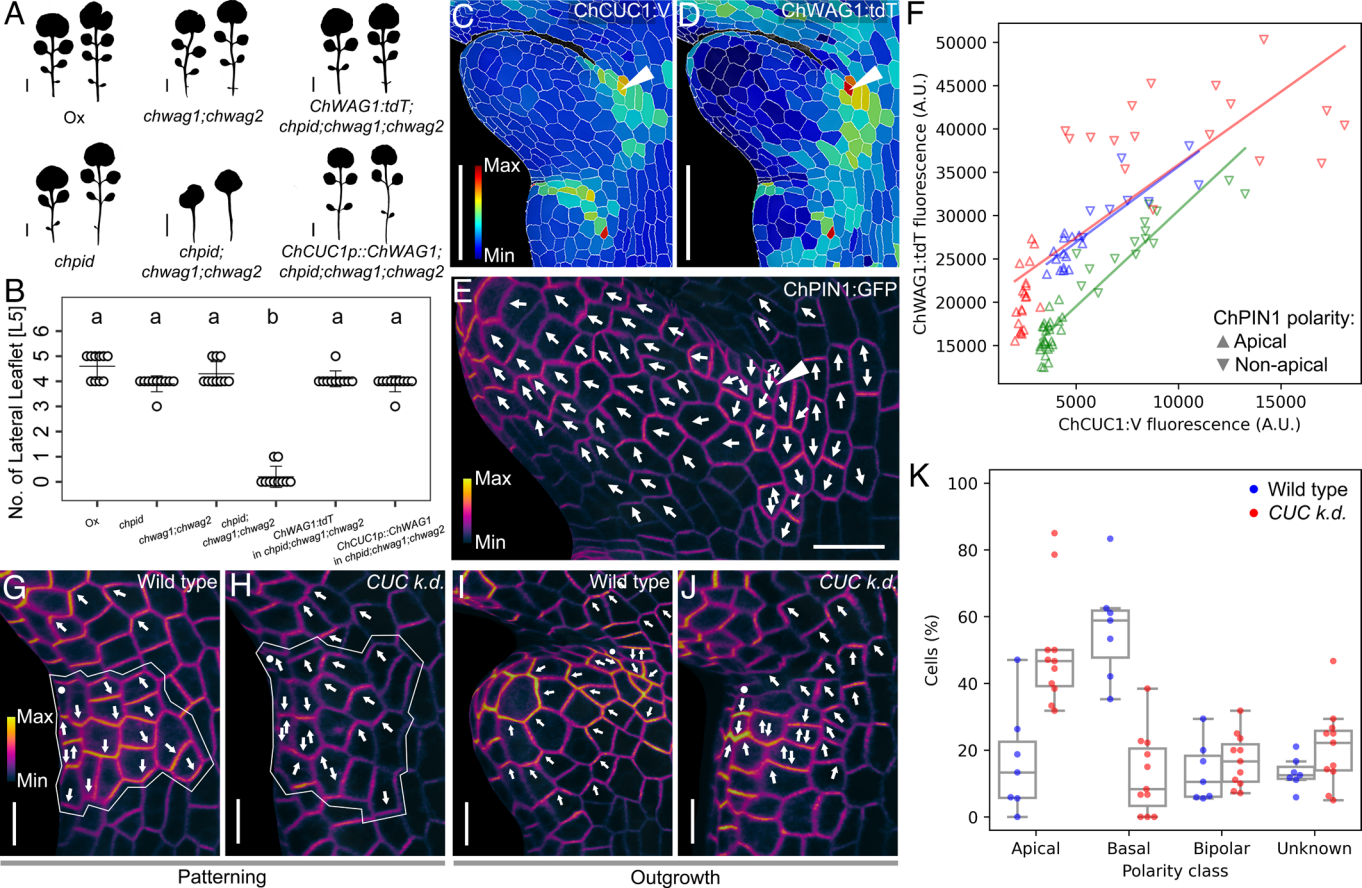
The ChCUC-ChWAG-ChPIN1 module in *C. hirsuta* leaf margin patterning. (*A* and *B*) Phenotypes of *C. hirsuta* strains from the indicated genotypes. (*A*) Silhouettes of rosette leaves 5 and 6 3 wk after sowing. (Scale bars: 1 cm.) (*B*) Number of lateral leaflets borne by leaf 5 (L5). The dot plot depicts mean values and SD (error bars). n ≥ 10 leaves per genotype. Letters a and b indicate statistically significant differences by the Kruskal–Wallis test with Dunn’s post hoc test (*α* = 0.05). (*C*–*F*) Quantification of the epidermal expression of *ChCUC1:V* (*C*) and *ChWAG1:tdT* (*D*), and the polarity of ChPIN1:GFP (*E*) in *C. hirsuta* leaf 5 during the development of lateral leaflets. White arrowheads: the same cell in images (*C*–*E*). White arrows: direction of ChPIN1:GFP accumulation. [Scale bars: 50 µm (*C* and *D*); 20 µm (*E*).] (*F*) Correlation between the expression of *ChCUC1:V* and *ChWAG1:tdT*, and the polarity of ChPIN1:GFP. Each color represents an independent leaf (*n* = 3). The solid lines indicate the predicted relationship between *ChCUC1:V* and *ChWAG1:tdT* expression calculated by linear regression on each sample independently: R^2^ = 0.78 (*P* = 5.1 × 10^−19^); 0.62 (6.5 × 10^−9^); 0.76 (1.5 × 10^−7^). Point biserial correlation test between *ChCUC1:V* expression and ChPIN1:GFP polarity (for each sample): 0.84 (*P* = 5.6 × 10^−12^); 0.81 (1.6 × 10^−9^); 0.80 (9.1 × 10^−6^). (*G*–*K*) Polarity dynamics of ChPIN1:GFP during patterning (*G* and *H*) and outgrowth (*I* and *J*) of the first lateral leaflet of *C. hirsuta* leaf 5 from wild-type and *ChCUC knock-down* (*CUC k.d.*) plants. White dot: boundary between terminal and lateral leaflet. White outline: region of interest (ROI, presumptive *CUC* domain defined as a grid of 4 × 4 cells originating from the white dot) used for the analysis shown in (*K*). White arrows: direction of ChPIN1:GFP accumulation in the ROI depicted in (*G* and *H*) and the leaf margin cells in (*I* and *J*). [Scale bars: 10 µm (*G*–*J*).] (*K*) Polarity frequency distribution of ChPIN1:GFP within the region of interest defined in (*G* and *H*). *n* (wild type) = 7 samples, *n* (*CUC k.d.*) = 11. Chi-squared test. *P* = 10^−23^.

If *CUC*-dependent transcriptional activation of *WAG* genes is important to regulate PIN1 function in leaflet formation, we predicted that loss-of-function phenotypes of *ChWAG* genes would cause leaf development defects comparable to those observed in mutants with reduced *ChPIN1* and *ChCUC* function (8, 32). To test this idea, we used CRISPR-Cas9 gene editing to generate loss-of-function alleles of *ChWAG1* and *ChWAG2*, and their potentially redundant paralogue *ChPINOID* (*ChPID*) (*SI Appendix*, Fig. S12). We observed that *chpid;chwag1;chwag2* triple and *chpid;chwag1* double mutants present a strong reduction in the number of LLs, including the occasional fusion of LLs with the rachis or with the TL (Fig. 4 *A* and *B* and *SI Appendix*, Fig. S13 *A* and *B*). By using multivariate shape analysis (*SI Appendix*, *Materials and Methods*), we also found that the TLs of *chpid;chwag1;chwag2* triple mutants occupy a position in shape-space similar to that of the *chpin1* and *ChCUC knock-down* TLs (*SI Appendix*, Fig. S13 *C* and *D*). These observations indicate that *ChCUCs*, *ChPID/ChWAGs*, and *ChPIN1* act in overlapping processes to control leaflet initiation and development. We next examined whether the specific expression of *ChWAG1* in the *ChCUC1* domain (*ChCUC1p::ChWAG1*) is sufficient to rescue the leaflet formation defects in the *chpid;chwag1;chwag2* triple mutant, and confirmed this to be the case (Fig. 4 *A* and *B*). Therefore, our genetic analyses are consistent with the molecular evidence that ChCUC1 directly activates *WAG* genes, which are known regulators of PIN1. Further evidence that *WAG* genes together with *PID* function in overlapping developmental processes with *PIN1* in *C. hirsuta* comes from the observation that *chpid;chwag1;chwag2* triple mutants resemble *chpin1* mutants and show naked inflorescence meristems, indicating a requirement of these genes in organ initiation at the shoot meristem (*SI Appendix*, Fig. S14 *A* and *B*) (8). Overall, our data indicate that ChCUC1 modulates PIN1 function at the leaf margin, at least in part via the direct activation of *WAGs*.

If ChCUC1 indeed acts in the way indicated above, then the expression of ChCUC1, ChWAG1, and ChPIN1 should overlap in space and time in developing leaf primordia. To test this, we simultaneously monitored the spatiotemporal expression dynamics of *ChCUC1:V*, *ChWAG1:tdT* (*ChWAG1p::ChWAG1:tdT*, Fig. 4*A*), and *ChPIN1:GFP* in early stages of leaf development (1 to 5 d after leaf initiation) at cellular resolution. We observed that *ChCUC1:V*, *ChWAG1:tdT*, and *ChPIN1:GFP* initially display continuous expression in the distal leaf margin (*SI Appendix*, Fig. S15 *A* and *A’’*). Later, *ChCUC1:V* and *ChWAG1:tdT* expression domains became discrete, flanking the TL (*SI Appendix*, Fig. S15 *B* and *B’*) defining domains that also showed high *ChPIN1:GFP* expression (*SI Appendix*, Fig. S15*B’’*). After the emergence of LLs, *ChCUC1:V* appeared in discrete expression domains between each LL (Fig. 4*C* and *SI Appendix*, Fig. S15*C*), which is reminiscent of the expression pattern of *ChCUC2:V* in *C. hirsuta* after LL initiation (43). *ChWAG1:tdT* and *ChPIN1:GFP* expression foci strongly coincided with *ChCUC1:V* at the boundary between LLs (Fig. 4 *C–E* and *SI Appendix*, Fig. S15 *C’* and *C’’*), and in the cells where ChPIN1 polarity reversals occur during the formation of new LLs (Fig. 4*E* and *SI Appendix*, Fig. S18*J*). Cell-level quantification of the signals from these three reporters revealed a positive correlation between the expression levels of ChWAG1:tdT and ChPIN1:GFP with ChCUC1:V (Fig. 4*F* and *SI Appendix*, Fig. S15*D*), and high expression of *ChCUC1:V* and *ChWAG1:tdT* predicted the reversal of ChPIN1:GFP polarity (Fig. 4*F*, triangle directions). In conclusion, we show that ChWAG1:tdT and ChPIN1:GFP colocalize with ChCUC1:V during both the patterning and emergence of LLs in *C. hirsuta* leaf primordia, consistent with the idea that transcriptional activation of *ChWAG1* by ChCUC1 regulates ChPIN1 function.

Two further lines of evidence support the idea that ChCUC1 action in *C. hirsuta* leaf development is in part mediated by ChPID/ChWAGs, in a process that involves PIN1 phosphorylation. First, a “phosphodead” ChPIN1 transgene (*ChPIN1p::ChPIN1_S1,2,3A_:GFP*), in which we mutated to alanine three conserved PIN1 serine residues (S1, S2, and S3) that are phosphorylated by PID/WAGs in *A. thaliana* (25–27), failed to rescue the simplified leaf phenotype of the *chpin1* mutant (*SI Appendix*, Fig. S16 *A–E*). This result indicates that ChPIN1_S1,2,3_ phosphorylation is required for *C. hirsuta* leaflet formation. Second, the ability of *ChCUC1:V* to increase the complexity of the *A. thaliana* leaf margin is suppressed in the *pid;wag1;wag2* mutant background (*SI Appendix*, Fig. S17 *A*–*C*). Taken together, these data indicate that direct transcriptional induction of WAG kinases by ChCUC1 plays a key role in PIN1-mediated *C. hirsuta* leaf margin complexity.

To better understand the significance of CUC function for ChPIN1 polarity and patterning in the leaf margin, we imaged *ChPIN1:GFP* and studied ChPIN1 polarity dynamics in wild-type and *ChCUC knock-down* leaves during the patterning of the first LL. In wild type, the majority (60%) of the cells in the *ChCUC1* domain (*SI Appendix*, *Materials and Methods*) showed basally oriented (reversed) ChPIN1:GFP polarity (Fig. 4 *G* and *K* and *SI Appendix*, Fig. S18 *K–N*), whereas in *ChCUC knock-down* samples, only 10% of these cells showed a basal orientation (Fig. 4 *H* and *K*). These patterning events were followed by the generation of a deep sinus and LL outgrowth in the wild-type leaves but not in the *ChCUC knock-down* leaves (Fig. 4 *I* and *J*). These data show that *ChCUC1*, together with its paralogues, is necessary for ChPIN1 polarity reversal during LL formation, consistent with the transcriptional activation of *ChWAGs* by ChCUC1.

### Conceptualizing the Instructive Role of CUCs on Auxin Patterning Using Computational Models.

**Fig. 5.**
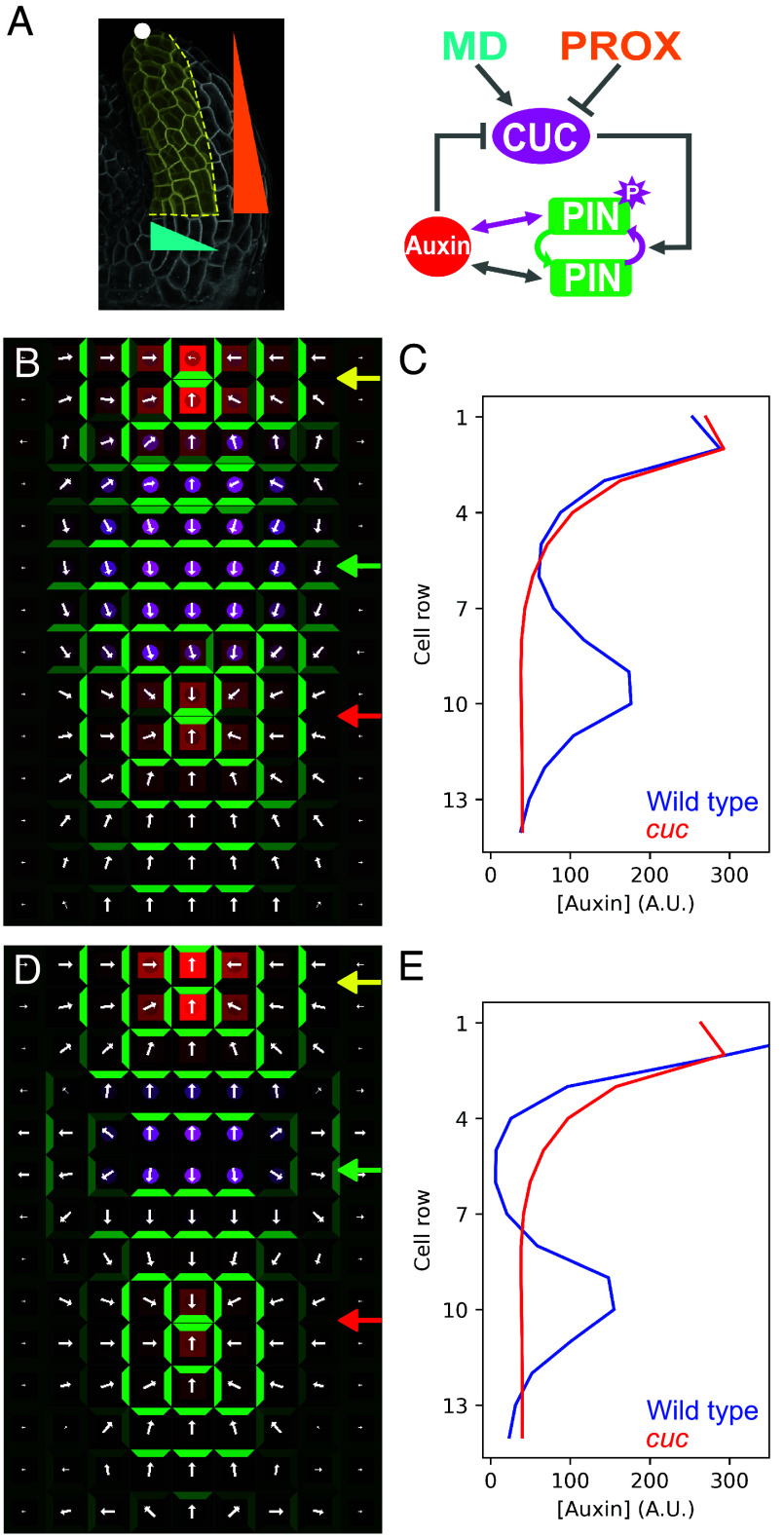
Computational models that modulate PIN1 properties in the *CUC* domain can account for local repolarization and formation of auxin maxima on the leaf margin. (*A*) Cartoon representing the tissue modeled (*Left*), which corresponds to the leaf margin during patterning of the first lateral auxin site and protrusion, and network that controls the levels of CUC/auxin and determines the activity and polarization mode of PIN1 (*Right*). The white dot indicates the apex of the leaf that typically displays an auxin activity maximum, and that has been modeled as an auxin source. The wedges indicate the spatial distribution of the middle domain (MD, cyan) and proximal domain (PROX, orange) identity factors used to model the CUC expression domain (for details see *SI Appendix*). The straight bidirectional arrows indicate the PIN-auxin feedback loops. (*B*–*E*) Output of the PIN polarization modulation model (CUC-induced PIN phosphorylation switches PIN polarization mode from up-the-gradient to with-the-flux, *B* and *C*) and the PIN efficiency modulation model (CUC-induced PIN phosphorylation increases PIN transport efficiency, *D* and *E*). (*B* and *D*) Simulations steady state of wild-type leaf margins showing a distal auxin maximum and associated polarity convergence (yellow arrow), CUC-dependent PIN1 repolarization (green arrow), and formation of an emergent convergence point (red arrow). CUC, PIN, and auxin are color coded as in (*A*). The white arrows indicate the direction of net PIN1-mediated auxin transport in each cell. (*C* and *E*) Auxin profiles along the central cell column (middle domain) of the simulations shown in (*B*) and (*D*) respectively (blue), and from control simulations without CUC (red) that fail to generate emergent auxin foci.

Previously, the role and behavior of PIN1 in organogenesis and leaf development have been explored using computational models based on feedback between auxin and PIN1: It has been proposed that PIN1 allocation to a plasma membrane segment can be promoted either by auxin efflux across the membrane [with-the-flux hypothesis—WTF (44, 45)] or by elevated auxin in the neighboring cells [up-the-gradient hypothesis—UTG (18–20)]. In a previous UTG-based leaf model (18) that reproduced the formation of polarity convergences and auxin maxima, CUC was ascribed the role of a “reversal enabling factor” that permitted spontaneous PIN1 reversals in response to changes in auxin distribution. Our data above provide mechanistic detail into leaf margin patterning, indicating that 1) CUC actively promotes PIN1 polarity reversals instead of simply enabling them, and that 2) CUC activates the expression of *WAG1* and *2*. These kinases can increase PIN1 transport activity (27, 46, 47) and influence its polarity, perhaps through modulating its affinity for alternative endosomal recycling pathways that target PIN1 to specific plasma membrane sections (26, 48–51). To test the importance of the CUC-WAG module on leaf margin patterning, we investigated whether computational models incorporating these insights could explain our experimental observations during leaf margin patterning. To this end, we modeled CUC as a factor that promotes the accumulation of phosphorylated PIN1 and assumed that this fraction of phosphorylated PIN1 either (a) polarizes WTF instead of UTG (PIN polarization modulation model, PMM), or (b) has increased transport efficiency (PIN efficiency modulation model, EMM) (Fig. 5*A*; see formal description of the model in *SI Appendix*), thereby allowing us to examine whether such instructive inputs of CUC on PIN1 polarity might be sufficient to explain our biological observations.

We first created a UTG template that captures the features of young leaf primordia: an auxin maximum at the distal tip and PIN1 polarities describing a convergent pattern with all margin cells polarized apically. To initiate these patterns, we set an auxin source (Fig. 5*A*, white dot) in the central cell of the second row. Then, we tested the PMM (Fig. 5 *B* and *C*) and observed that patterned CUC accumulation (Fig. 5*A*) and the associated promotion of WTF polarization, caused polarity reversals in several cells (Fig. 5*B* and Movie S1). The resulting basal auxin transport led to an incipient localized auxin accumulation at the base of the CUC domain, subsequently sensed by adjacent UTG cells, and amplified by the auxin-PIN feedback loop. We next explored the EMM (Fig. 5 *D* and *E*). The increased auxin transport in the CUC cells altered the auxin gradient, resulting in PIN apical to basal repolarization (Fig. 5*D* and Movie S2). As in the previous model, this local polarity switch led to the formation of a basally adjacent PIN convergence and an auxin maximum. These features did not emerge in either of the models when simulating a *cuc* mutant template (Fig. 5 *C* and *E*). As biological systems are inherently noisy, we examined the robustness of our models to determine whether they could reproduce our observations in the presence of different levels of noise in auxin or CUC concentrations. We found that both models retained their ability to repolarize PIN and generate a new auxin maximum under moderate noise ([Auxin] ±10% or [CUC] ± 30%; *SI Appendix*, Figs. S19 and S20). While the position stability of the emergent auxin peak was comparable between the two models, the amplitude was more predictable in the PMM. The similar robustness of these two models likely reflects the fact that, although the events that lead to PIN repolarization are different in each model, the resulting auxin distribution is similar (Fig. 5 *C* and *E*).

In summary, these simulations indicate that patterned modulation of PIN1 polarization mode and/or activity can account for key findings from our ChCUC1 fate mapping and genetic mosaic experiments: First, the cell-autonomous reversals of PIN1 polarity observed in the *ChCUC1*-expressing cells (compare the cells marked with green arrows/asterisks in the simulations in Fig. 5 *B* and *D* and in the leaf with a ChCUC1 clone in Fig. 2 *G*–*G’’*), and second, the non-cell-autonomous organization of PIN1 polarity convergences and creation of new auxin sites for organ initiation (cells marked with red arrows/asterisks in Fig. 5 *B* and *D* and Fig. 2 *G*–*G’’*). A difference of the two models is that while the PMM considers CUC as a direct regulator of PIN polarization, in the EMM the effect of CUC on PIN polarity emerges indirectly through its effects on auxin concentration, which in turn are a result of increased auxin transport efficiency. This difference likely underlies the more pronounced overlap of cells forming the convergence with the CUC domain in the PMM compared to the EMM. However, these models are not mutually exclusive, and clarifying the relative significance of ChCUC1-mediated modulation of PIN1 transport activity versus its mechanism of polarization in relation to auxin will require future work. This will include analyzing PIN1 polarities and phosphorylation after experimental perturbation of auxin dynamics in the CUC domain, and the generation of 3D growing cellular models, which is technically challenging. The models we present here provide a starting point for such future studies, as they incorporate the CUC1 PIN1 interaction in a more mechanistically rooted manner, replacing more phenomenological assumptions present in previous models of leaf margin patterning (18). From a developmental perspective, CUC patterned expression can be conceptualized as a regulatory layer that modulates the PIN-auxin self-organizing system in specific domains to promote repolarization events, and thus makes auxin-mediated marginal patterning more flexible during development and evolution. This behavior is similar to that observed during the radially arranged digit patterning in the mouse limb bud, where a gradient of Fgf signaling adjusts the wavelength of a reaction–diffusion system to ensure correct digit formation (52).

## Concluding Remarks

Overall, our findings show how patterned expression of the ChCUC1 transcription factor early in leaf development can, through activation of its downstream effectors, modulate cell polarity, organize tissue patterning, and regulate cell growth to sculpt organ shape. We also demonstrate how species-specific tinkering with this process can contribute to morphological diversity between species. In conclusion, we conceptualized how a specific genetic cascade acts across biological scales to cause diversification of plant form.

Our findings also raise the question of how the action of CUC1 in driving leaf shape diversity that we report here is integrated with the function of other known determinants of evolutionary diversification of leaf complexity. *Class I KNOTTED1-LIKE HOMEOBOX* (*KNOX1*) genes, which encode TALE (Three-Amino acid Loop Extension) homeodomain proteins, promote leaflet formation, and have contributed to diversification of leaf form (53–55). They are thought to delay differentiation and increase the competence of leaf margins to respond to auxin-based patterning (8, 9). There is also evidence for positive feedback between *KNOX1* and *CUC* genes (32, 56), so this interaction might modulate the CUC-mediated growth polarization that underpins leaflet formation. Thus, it is likely that the KNOX1-CUC module contributes to both delaying differentiation and facilitating auxin-mediated PIN1 polarization. Further investigation particularly using KNOX1 genetic mosaics will help further understand the role of this mutual reinforcement between CUC and KNOX1 in leaflet outgrowth. *REDUCED COMPLEXITY* (*RCO*) is a different type of homeobox gene -belonging to the HD-ZIP I class- that contributed to the evolution of leaf complexity (39). *RCO* is expressed proximally to *CUC2* in emerging leaflets where it represses growth locally, and it acts postpatterning, as opposed to *CUC* genes that also act during patterning (Fig. 1) (43). RCO and CUC2 (which acts redundantly with its paralogue CUC1), have been shown to largely operate independently at the leaf margin (43). Therefore, in the future, it will be important to test whether the concurrent expression of *RCO* and *CUC1* from their endogenous regulatory elements is sufficient to cause generation of leaflets in the simple leaves of *A. thaliana*, as opposed to their individual expression which causes leaf lobing (39) (Fig. 1).

It is of note that CUCs, PIN1, and PID/WAGs regulate many developmental processes including phyllotaxis, floral organ development, and embryonic tissue patterning, so it is likely that the mechanisms we characterize here are broadly important for plant development. For example, direct regulation of WAGs by CUCs may also contribute to PIN1 repolarization at the SAM boundary, enabling new PIN1 convergences underlying iterative organogenesis at the SAM (30), consistent with the observed upregulation of *PID* expression in the SAM boundary domain (28, 57). To test these ideas, it will be important to understand whether the phyllotactic defects reported in *cuc* mutants (58, 59) reflect reduced expression of *PID/WAG* genes, mutants which also have aberrant phyllotaxis (15, 60). Finally, future computational and experimental work will be required to evaluate the precise manner in which other genetic and mechanical inputs (2, 23) are integrated alongside the processes we described here to shape plant form.

## Materials and Methods

Full description of the materials used and protocol-level methods are shown in *SI Appendix*, including gene and mutant nomenclature, plant materials used and growth conditions, and generation of expression constructs; generation and verification of transgenic plants; generation of mutant alleles using CRISPR/Cas9; analysis of macroscopic phenotypes; dexamethasone induction of ChCUC1 for confocal imaging; generation of ChCUC1 mosaics; confocal scanning laser microscopy (CSLM); generation of curved 2D meshes for downstream analyses; analyses of PIN1:GFP polarity; quantification of tissue and cell parameters in MorphographX; dexamethasone induction of ChCUC1 for transcriptomic analysis; RNA extraction, qRT-PCR, and transcriptomic analysis; chromatin immunoprecipitation sequencing (ChIP-seq); DNA affinity purification sequencing (DAP-seq); transcriptome sequencing data analysis; ChIP-seq and DAP-seq data analysis; replication, statistical analyses, plotting.

## Supplementary Material

Appendix 01 (PDF)

Dataset S01 (XLSX)

Dataset S02 (XLSX)

Dataset S03 (XLSX)

Movie S1. **Simulation of leaf margin patterning using the PIN polarization modulation model.** This movie corresponds to a simulation using the parameter values described in Table S5 (PIN polarization modulation model). Auxin, red; PIN, green; CUC, magenta.

Movie S2. **Simulation of leaf margin patterning using the PIN efficiency modulation model.** This movie corresponds to a simulation using the parameter values described in Table S5 (PIN efficiency modulation model). Auxin, red; PIN, green; CUC, magenta.

## Data Availability

Additional data to support the conclusions of this study can be found as *SI Appendix*. Short sequence data have been deposited at the Gene Expression Omnibus (RNA-seq: GSE241051 (61), ChIP-seq: GSE242999 (62), DAP-seq: GSE241208) (63).

## References

[1] M. T. Butler, J. B. Wallingford, Planar cell polarity in development and disease. Nat. Rev. Mol. Cell Biol. **18**, 375–388 (2017). 28293032 10.1038/nrm.2017.11 PMC5826606

[2] E. Coen, R. Kennaway, C. Whitewoods, On genes and form. Development **144**, 4203–4213 (2017). 29183934 10.1242/dev.151910

[3] V. Gorelova, J. Sprakel, D. Weijers, Plant cell polarity as the nexus of tissue mechanics and morphogenesis. Nat. Plants **7**, 1548–1559 (2021). 34887521 10.1038/s41477-021-01021-w

[4] M. G. Heisler, Integration of core mechanisms underlying plant aerial architecture. Front. Plant Sci. **12**, 786338 (2021). 34868186 10.3389/fpls.2021.786338 PMC8637408

[5] J. J. Ramalho, V. A. S. Jones, S. Mutte, D. Weijers, Pole position: How plant cells polarize along the axes. Plant Cell **34**, 174–192 (2022). 34338785 10.1093/plcell/koab203 PMC8774072

[6] J. Raspopovic, L. Marcon, L. Russo, J. Sharpe, Digit patterning is controlled by a Bmp-Sox9-Wnt Turing network modulated by morphogen gradients. Science **345**, 566–570 (2014). 25082703 10.1126/science.1252960

[7] E. E. Kuchen , Generation of leaf shape through early patterns of growth and tissue polarity. Science **335**, 1092–1096 (2012). 22383846 10.1126/science.1214678

[8] M. Barkoulas, A. Hay, E. Kougioumoutzi, M. Tsiantis, A developmental framework for dissected leaf formation in the *Arabidopsis* relative *Cardamine hirsuta*. Nat. Genet. **40**, 1136–1141 (2008). 19165928 10.1038/ng.189

[9] D. Kierzkowski , A growth-based framework for leaf shape development and diversity. Cell **177**, 1405–1418.e1417 (2019). 31130379 10.1016/j.cell.2019.05.011 PMC6548024

[10] P. A. Lawrence, Last hideout of the unknown? Nature **429**, 247–247 (2004). 15152230 10.1038/429247a

[11] T. Lecuit, L. Le Goff, Orchestrating size and shape during morphogenesis. Nature **450**, 189–192 (2007). 17994084 10.1038/nature06304

[12] C. D. Whitewoods , Evolution of carnivorous traps from planar leaves through simple shifts in gene expression. Science **367**, 91–96 (2020). 31753850 10.1126/science.aay5433

[13] K. Okada, J. Ueda, M. K. Komaki, C. J. Bell, Y. Shimura, Requirement of the auxin polar transport system in early stages of *Arabidopsis* floral bud formation. Plant Cell **3**, 677–684 (1991). 12324609 10.1105/tpc.3.7.677 PMC160035

[14] E. Benková , Local, efflux-dependent auxin gradients as a common module for plant organ formation. Cell **115**, 591–602 (2003). 14651850 10.1016/s0092-8674(03)00924-3

[15] D. Reinhardt , Regulation of phyllotaxis by polar auxin transport. Nature **426**, 255–260 (2003). 14628043 10.1038/nature02081

[16] A. B. Rebocho, P. Southam, J. R. Kennaway, J. A. Bangham, E. Coen, Generation of shape complexity through tissue conflict resolution. eLife **6**, e20156 (2017). 28166865 10.7554/eLife.20156 PMC5295819

[17] N. Bhatia , Auxin acts through MONOPTEROS to regulate plant cell polarity and pattern phyllotaxis. Curr. Biol. **26**, 3202–3208 (2016). 27818174 10.1016/j.cub.2016.09.044 PMC5154752

[18] G. D. Bilsborough , Model for the regulation of *Arabidopsis thaliana* leaf margin development. Proc. Natl. Acad. Sci. U.S.A. **108**, 3424–3429 (2011). 21300866 10.1073/pnas.1015162108 PMC3044365

[19] H. Jönsson, M. G. Heisler, B. E. Shapiro, E. M. Meyerowitz, E. Mjolsness, An auxin-driven polarized transport model for phyllotaxis. Proc. Natl. Acad. Sci. U.S.A. **103**, 1633–1638 (2006). 16415160 10.1073/pnas.0509839103 PMC1326488

[20] R. S. Smith , A plausible model of phyllotaxis. Proc. Natl. Acad. Sci. U.S.A. **103**, 1301–1306 (2006). 16432192 10.1073/pnas.0510457103 PMC1345713

[21] O. Hamant , Developmental patterning by mechanical signals in *Arabidopsis*. Science **322**, 1650–1655 (2008). 19074340 10.1126/science.1165594

[22] M. G. Heisler , Alignment between PIN1 polarity and microtubule orientation in the shoot apical meristem reveals a tight coupling between morphogenesis and auxin transport. PLoS Biol. **8**, e1000516 (2010). 20976043 10.1371/journal.pbio.1000516 PMC2957402

[23] E. Coen, D. J. Cosgrove, The mechanics of plant morphogenesis. Science **379**, eade8055 (2023). 36730409 10.1126/science.ade8055

[24] A. Kareem, N. Bhatia, C. Ohno, M. G. Heisler, PIN-FORMED1 polarity in the plant shoot epidermis is insensitive to the polarity of neighboring cells. iScience **25**, 105062 (2022). 36157591 10.1016/j.isci.2022.105062 PMC9494258

[25] P. Dhonukshe , Plasma membrane-bound AGC3 kinases phosphorylate PIN auxin carriers at TPRXS (N/S) motifs to direct apical PIN recycling. Development **137**, 3245–3255 (2010). 20823065 10.1242/dev.052456

[26] F. Huang , Phosphorylation of conserved PIN motifs directs *Arabidopsis* PIN1 polarity and auxin transport. Plant Cell **22**, 1129–1142 (2010). 20407025 10.1105/tpc.109.072678 PMC2879764

[27] M. Zourelidou , Auxin efflux by PIN-FORMED proteins is activated by two different protein kinases, D6 PROTEIN KINASE and PINOID. eLife **3**, e02860 (2014). 24948515 10.7554/eLife.02860 PMC4091124

[28] M. Furutani , *PIN-FORMED1* and *PINOID* regulate boundary formation and cotyledon development in *Arabidopsis* embryogenesis. Development **131**, 5021–5030 (2004). 15371311 10.1242/dev.01388

[29] F. Galbiati , An integrative model of the control of ovule primordia formation. Plant J. **76**, 446–455 (2013). 23941199 10.1111/tpj.12309

[30] M. G. Heisler , Patterns of auxin transport and gene expression during primordium development revealed by live imaging of the *Arabidopsis* inflorescence meristem. Curr. Biol. **15**, 1899–1911 (2005). 16271866 10.1016/j.cub.2005.09.052

[31] Y. Berger , The NAC-domain transcription factor GOBLET specifies leaflet boundaries in compound tomato leaves. Development **136**, 823–832 (2009). 19176589 10.1242/dev.031625

[32] T. Blein , A conserved molecular framework for compound leaf development. Science **322**, 1835–1839 (2008). 19095941 10.1126/science.1166168

[33] A. Hasson , Evolution and diverse roles of the *CUP-SHAPED COTYLEDON* genes in *Arabidopsis* leaf development. Plant Cell **23**, 54–68 (2011). 21258003 10.1105/tpc.110.081448 PMC3051246

[34] M. I. Rast-Somssich , Alternate wiring of a *KNOXI* genetic network underlies differences in leaf development of *A. thaliana* and *C. hirsuta*. Genes Dev. **29**, 2391–2404 (2015). 26588991 10.1101/gad.269050.115 PMC4691893

[35] L. A. Nikolov, M. Tsiantis, Interspecies gene transfer as a method for understanding the genetic basis for evolutionary change: Progress, pitfalls, and prospects. Front. Plant Sci. **6**, 1135 (2015). 26734038 10.3389/fpls.2015.01135 PMC4686936

[36] B. Sauer, Functional expression of the *cre-lox* site-specific recombination system in the yeast *Saccharomyces cerevisiae*. Mol. Cell. Biol. **7**, 2087–2096 (1987). 3037344 10.1128/mcb.7.6.2087-2096.1987 PMC365329

[37] N. Hervieux , A mechanical feedback restricts sepal growth and shape in *Arabidopsis*. Curr. Biol. **26**, 1019–1028 (2016). 10.1016/j.cub.2016.03.004 27151660

[38] L. Serra, C. Perrot-Rechenmann, Spatiotemporal control of cell growth by CUC3 shapes leaf margins. Development **147**, dev183277 (2020). 32094116 10.1242/dev.183277

[39] D. Vlad , Leaf shape evolution through duplication, regulatory diversification, and loss of a homeobox gene. Science **343**, 780–783 (2014). 24531971 10.1126/science.1248384

[40] S. Lindemose , A DNA-binding-site landscape and regulatory network analysis for NAC transcription factors in *Arabidopsis thaliana*. Nucleic Acids Res. **42**, 7681–7693 (2014). 24914054 10.1093/nar/gku502 PMC4081100

[41] R. C. O’Malley , Cistrome and epicistrome features shape the regulatory DNA landscape. Cell **165**, 1280–1292 (2016). 27203113 10.1016/j.cell.2016.04.038 PMC4907330

[42] C. Tian , An organ boundary-enriched gene regulatory network uncovers regulatory hierarchies underlying axillary meristem initiation. Mol. Syst. Biol. **10**, 755 (2014). 25358340 10.15252/msb.20145470 PMC4299377

[43] N. Bhatia , Interspersed expression of *CUP-SHAPED COTYLEDON2* and *REDUCED COMPLEXITY* shapes *Cardamine hirsuta* complex leaf form. Curr. Biol. **33**, 2977–2987.e2976 (2023). 37453425 10.1016/j.cub.2023.06.037

[44] G. J. Mitchison, S. Brenner, A model for vein formation in higher plants. Proc. R. Soc. Lond., Ser. B: Biol. Sci. **207**, 79–109 (1980).

[45] A.-G. Rolland-Lagan, P. Prusinkiewicz, Reviewing models of auxin canalization in the context of leaf vein pattern formation in *Arabidopsis*. Plant J. **44**, 854–865 (2005). 16297075 10.1111/j.1365-313X.2005.02581.x

[46] K. L. Ung , Structures and mechanism of the plant PIN-FORMED auxin transporter. Nature **609**, 605–610 (2022). 35768502 10.1038/s41586-022-04883-y PMC9477730

[47] P. Wang , Phosphatidic acid directly regulates PINOID-dependent phosphorylation and activation of the PIN-FORMED2 auxin efflux transporter in response to salt stress. Plant Cell **31**, 250–271 (2019). 30464035 10.1105/tpc.18.00528 PMC6391703

[48] J. Kleine-Vehn , PIN auxin efflux carrier polarity is regulated by PINOID kinase-mediated recruitment into GNOM-independent trafficking in *Arabidopsis*. Plant Cell **21**, 3839–3849 (2009). 20040538 10.1105/tpc.109.071639 PMC2814515

[49] M. Michniewicz , Antagonistic regulation of PIN phosphorylation by PP2A and PINOID directs auxin flux. Cell **130**, 1044–1056 (2007). 17889649 10.1016/j.cell.2007.07.033

[50] J. Zhang, T. Nodzyński, A. Pěnčík, J. Rolčík, J. Friml, PIN phosphorylation is sufficient to mediate PIN polarity and direct auxin transport. Proc. Natl. Acad. Sci. U.S.A. **107**, 918–922 (2010). 20080776 10.1073/pnas.0909460107 PMC2818920

[51] J. Friml , A PINOID-dependent binary switch in apical-basal PIN polar targeting directs auxin efflux. Science **306**, 862–865 (2004). 15514156 10.1126/science.1100618

[52] J. B. Green, J. Sharpe, Positional information and reaction-diffusion: Two big ideas in developmental biology combine. Development **142**, 1203–1211 (2015). 25804733 10.1242/dev.114991

[53] D. Hareven, T. Gutfinger, A. Parnis, Y. Eshed, E. Lifschitz, The making of a compound leaf: Genetic manipulation of leaf architecture in Tomato. Cell **84**, 735–744 (1996). 8625411 10.1016/s0092-8674(00)81051-x

[54] G. Bharathan , Homologies in leaf form inferred from *KNOXI* gene expression during development. Science **296**, 1858–1860 (2002). 12052958 10.1126/science.1070343

[55] A. Hay, M. Tsiantis, The genetic basis for differences in leaf form between *Arabidopsis thaliana* and its wild relative *Cardamine hirsuta*. Nat. Genet. **38**, 942–947 (2006). 16823378 10.1038/ng1835

[56] S. V. Spinelli, A. P. Martin, I. L. Viola, D. H. Gonzalez, J. F. Palatnik, A mechanistic link between *STM* and *CUC1* during *Arabidopsis* development. Plant Physiol. **156**, 1894–1904 (2011). 21685178 10.1104/pp.111.177709 PMC3149926

[57] B. Landrein , Mechanical stress contributes to the expression of the *STM* homeobox gene in *Arabidopsis* shoot meristems. eLife **4**, e07811 (2015). 26623515 10.7554/eLife.07811 PMC4666715

[58] A. Peaucelle, H. Morin, J. Traas, P. Laufs, Plants expressing a *miR164*-resistant *CUC2* gene reveal the importance of post-meristematic maintenance of phyllotaxy in *Arabidopsis*. Development **134**, 1045–1050 (2007). 17251269 10.1242/dev.02774

[59] A. Burian , The *CUP-SHAPED COTYLEDON2* and *3* genes have a post-meristematic effect on *Arabidopsis thaliana* phyllotaxis. Ann. Bot. **115**, 807–820 (2015). 25681504 10.1093/aob/mcv013 PMC4373294

[60] S. R. Bennett, J. Alvarez, G. Bossinger, D. R. Smyth, Morphogenesis in *pinoid* mutants of *Arabidopsis thaliana*. Plant J. **8**, 505–520 (1995).

[61] Z. Hu, M. Tsiantis, Project: GSE241051. Gene Expression Omnibus. https://www.ncbi.nlm.nih.gov/geo/query/acc.cgi?acc=GSE241051. Deposited 17 August 2023.

[62] Z. Hu, M. Tsiantis, Project: GSE242999. Gene Expression Omnibus. https://www.ncbi.nlm.nih.gov/geo/query/acc.cgi?acc=GSE242999. Deposited 12 September 2023.

[63] Z. Hu, M. Tsiantis, Project: GSE241208. Gene Expression Omnibus. https://www.ncbi.nlm.nih.gov/geo/query/acc.cgi?acc=GSE241208. Deposited 18 August 2023.

