# Supplementary material for: A CUC1/auxin genetic module links cell polarity to patterned tissue growth and leaf shape diversity in crucifer plants
Source: Proc Natl Acad Sci U S A. 2024 Jun 21;121(26):e2321877121. doi: 10.1073/pnas.2321877121 (PMC11214078; doi:10.1073/pnas.2321877121)
Supplement: Supplementary file 1 — Appendix 01 (PDF) [file pnas.2321877121.sapp.pdf]

Supporting Information for

A CUC1/auxin genetic module links cell polarity to patterned tissue growth and leaf shape diversity in crucifer plants

Zi-Liang Hu, David Wilson-Sánchez, Neha Bhatia, Madlen I. Rast-Somssich, Anhui Wu, Daniela Vlad, Liam McGuire, Lachezar A. Nikolov, Patrick Laufs, Xiangchao Gan, Stefan Laurent, Adam Runions, Miltos Tsiantis

Miltos Tsiantis

Email: tsiantis@mpipz.mpg.de

This PDF file includes:

Supporting text

Figures S1 to S20

Tables S1 to S5

Legends for Movies S1 to S2

Legends for Datasets S1 to S3

SI References

Other supporting materials for this manuscript include the following:

Movies S1 to S2

Datasets S1 to S3

Supporting Information Text

Supplementary materials and methods

Gene and mutant nomenclature

In this work we use genes and proteins from *Arabidopsis thaliana* and *Cardamine hirsuta*. All of them were originally described in *A. thaliana*. Therefore, when using a gene name, we do not add the *At* prefix unless if necessary to avoid confusion between *A. thaliana* and *C. hirsuta*. By contrast, we always use the *Ch* prefix when referring to a gene from *C. hirsuta*. In sentences intended as discussion, gene names without species prefix refer to a whole orthologous group. In the section describing computer models, the name CUC is meant to comprise both CUC1 and CUC2.

Plant material and growth conditions

All mutants and transgenic plants used were in *C. hirsuta* Oxford (Ox) (*50*) or *A. thaliana* Columbia-0 (Col-0) backgrounds and are listed in Table S1. Primers for genotyping are described in Table S2. Plants were grown either in a greenhouse or in a climatic chamber. For plants grown in the greenhouse, seeds were sowed on soil and stratified for at least one day at 4°C (generally 3 days for *A. thaliana* and 7 days for *C. hirsuta*). Unless stated otherwise, plants were grown under long-day conditions (16h light/8h dark, supplementary lightening provided when natural light intensity was below 75 µmol m^{-2} s^{-1}) at 20±2°C. For plants grown in climatic chamber, seeds were sterilized by shaking in a solution with 2.4% sodium hypochlorite and 1% Tween20 for 10 minutes. Seeds were then washed two times with sterilized water and resuspended in sterilized 0.1% agarose. Finally, they were sown on ½ MS medium.

Generation of expression constructs

All transgenes were constructed using standard cloning techniques. All finished constructs were verified by sequencing prior to plant transformation. The sequences of the primers mentioned are listed in Table S3.

CUC1p::CUC1g:Venus. The *AtCUC1* promoter (1462 bp) was PCR amplified using Col-0 genomic DNA as the template, and AtCUC1p-SalI-F and AtCUC1p-XhoI-R as primers. The PCR product (1481 bp) was subcloned into pBJ36-*linker:Venus::OCSter* using the SalI and XhoI restriction sites and sequenced. The *AtCUC1* transcribed region (1521 bp) was PCR amplified using Col-0 genomic DNA as the template, and AtCUC1g-XhoI-F and AtCUC1g-NcoI-R as primers. The DNA fragment was digested with XhoI/NcoI, ligated into pBJ36-*AtCUC1p::linker:Venus::OCSter*, and then sequenced. The *AtCUC1p::AtCUC1g:Venus::OCSter* cassette was excised from the pBJ36 plasmid using NotI restriction enzyme and ligated into the binary plasmid pMLBART (1) for plant transformation.

ChCUC1p::ChCUC1g:Venus. The *ChCUC1* promoter region (1921 bp) plus transcribed region (without stop codon and 3'UTR, 1346 bp) was PCR amplified using *C. hirsuta* Ox genomic DNA as the template, and ChCUC1p-BamHI-F and ChCUC1-NcoI-R as primers. The PCR product (3282 bp) was cloned into pGEMTeasy and sequenced. The DNA fragment was then digested with BamHI/NcoI and ligated into pBJ97 containing *Linker:Venus::OCSter* (derived from pBJ97-*STM:Venus::OCSter*). The *ChCUC1p::ChCUC1g:Venus::OCSter* cassette was excised from the pBJ97 plasmid using NotI restriction enzyme and ligated into the binary plasmid pMLBART for plant transformation.

ChCUC2p::ChCUC2g:Venus. The generation of this construct is described in (2).

ChCUC1p::LhG4:GR; Op::ChCUC1:tdTomato and *ChRCOp::LhG4:GR; Op::ChCUC1:Venus.* These dexamethasone-inducible two-component constructs were assembled using the pOpIn2 vector [a kind gift from Ian Moore; (3)]. First, the *ChCUC1* and *ChRCO* promoters were amplified from *C. hirsuta* Ox genomic DNA using the *attB*-containing primers attB1-ChCUC1p-F/attB2-ChCUC1p-R and attB1-ChRCOp-F/attB2-ChRCOp-R, respectively. The amplified sequences encompass, respectively, the 1921 and 3231 bps immediately upstream of the translation start codons. The purified PCR products were recombined into pDONR207 by BP reaction to create Gateway entry clones, which were then recombined by LR reaction with the helper vector pBIN-LR-LhGR2 (a kind gift from Ian Moore) upstream of the *LhG4:GR* sequence. Subsequently, the whole cassettes (*Pro::LhG4:GR::term*) were subcloned into pOpIn2 via restriction-ligation using AscI restriction enzyme sites. The *attL1-ChCUC1cds:Venus-attL2* and *attL1-ChCUC1cds:tdTomato-attL2* fusions were synthesized and cloned into pUC57-Amp by GenScript (GenScript Biotech Corp., Piscataway, New Jersey, USA), and later subcloned in pOpIn2 via Gateway LR reactions downstream from the *pOp6* sequence. The final construct *ChCUC1p::LhG4:GR; Op::ChCUC1:tdTomato* was transformed in a *PIN1p::PIN1:GFP A. thaliana* line (4) and *ChRCOp::LhG4:GR; Op::ChCUC1:Venus* was transformed in *C. hirsuta* Ox plants.

35Sp::lox-spacer-lox::ChCUC1:Venus. First, the fragment *attL1-ChCUC1:Venus-attL2* was synthesized and inserted in pUC57-Amp by GenScript. The final construct was created by Multisite Gateway LR reaction using the entry vectors *P1R4-35S:lox-spacer-lox* (5), *R2R2-ChCUC1:Venus* and *P2RP3e-nosT2* (5), and the pBm43GW destination vector (6). The resulting plasmid was transformed into *A. thaliana* Col-0 *HSp::dBox:Cre; PIN1p::PIN1:GFP*. The *P1R4-35S:lox-spacer-lox* plasmid and the original *HSp::dBox:Cre* line were kind gifts from Ari Pekka Mähönen (University of Helsinki).

pIX-HALO-ChCUC1. The fragment *attL1-ChCUC1cds-attL2* was synthesized and inserted in pUC57-Kan by Genscript. The final construct was created via Gateway LR reaction using the entry vector pUC57-Kan-attL1-ChCUC1c-attL2 and destination vector pIX-HALO (a kind gift from Thomas Hartwig, Max Planck Institute for Plant Breeding Research).

ECp::SpCas9/AtU6p::ChPID ChWAG1 ChWAG2 sgRNA. The CRISPR modular system was used to generate the final vector. The attL5 and attL2 flanking *AtU6p::ChPID ChWAG1 ChWAG2 sgRNAs* block (*ChPID ChWAG1 ChWAG2 sgRNAs* driven by the RNA polymerase III promoter *AtU6p*) was synthesized and cloned into pUC57-Amp by GenScript. The attL1 and attR5 flanking *ECp::SpCas9* block [*SpCas9* driven by egg cell promoter *ECp*; (7, 8)] was synthesized and cloned into pUC57-Amp by GenScript. The pPZP200 harboring *OLE1p::OLE1:RFP* cassette and attR1 and attR2 flanking *ccdB* gene was used as the backbone. MultiSite Gateway LR reaction was performed to recombine all three blocks together. The final construct was transformed in *C. hirsuta* Ox plants.

ChWAG1p::ChWAG1:tdT. The *ChWAG1* promoter (3336 bp) was PCR amplified using *C. hirsuta* Ox genomic DNA as template, and *ChWAG1p*-EcoRI-F and *ChWAG1p*-XmaI-R as primers. The PCR product (3355 bp) was subcloned into pBJ36-*linker:tdTomato::OCSter* (tdTomato with linker sequence (5'-ggagctggtgcaggcgctggagccggtgcc-3') flanked by BamH1 and Xba1 was present in pBJ36) using EcoR1 and XmaI sites. The *ChWAG1* transcribed region (without 5'UTR, stop codon, and 3'UTR, 1482 bp) was PCR amplified using *C. hirsuta* Ox genomic DNA as the template, and ChWAG1c-XmaI-F and ChWAG1c-BamHI-R as primers. The DNA fragment (1501bp) was then digested with XmaI and BamHI, and ligated into pBJ36-*ChWAG1p:linker:tdTomato::OCSter*, and then sequenced. The *ChWAG1p::ChWAG1:tdTomato::OCSter* cassette was excised from the pBJ36 plasmid using NotI restriction enzyme and ligated into the binary plasmid pMLHYG for *C. hirsuta* Ox plant transformation.

ChPIDp::ChPID:eGFP. The *ChPINOID* promoter (3608 bp) was PCR amplified using *C. hirsuta* Ox genomic DNA as the template, and ChPIDp-EcoRI-F and ChPIDp-XmaI-R as primers. The DNA fragment was digested with EcoRI/XmaI, ligated into pBJ36, and then sequenced. The *ChPIDcds:linker:2XeGFP* cassette (2814 bp, *ChPINOID* coding sequence is 1317bp) was synthesized and cloned into pUC57-Kan by GenScript, and subcloned into pBJ36-*ChPIDp::OCSter* using XmaI/ClaI double digestion. The *ChPIDp::ChPIDg:eGFP::OCSter* cassette was excised from the pBJ36 plasmid using NotI restriction enzyme and ligated into the binary plasmid pMLHYG for *C. hirsuta* Ox plant transformation.

ChPIN1p::ChPIN1g:eGFP. The *ChPIN1* promoter (3506 bp) was PCR amplified using *C. hirsuta* Ox genomic DNA as template and ChPIN1p-XhoI-F and ChPIN1p-KpnI-R as primers. The PCR product (3524 bp) was subcloned in pBJ36 using the XhoI and KpnI restriction sites and sequenced. The *ChPIN1g:eGFP* fusion (6128 bp, including introns and 3'UTR, synthesized by GenScript) was from the start codon to 2181 bp downstream of the stop codon. An *eGFP* sequence flanked by linkers on both sites was inserted into position 651 (9). The *ChPIN1g:eGFP* fragment was then digested with KpnI/BamHI and ligated into pBJ36-ChPIN1p::OCSter. The *ChPIN1p::ChPIN1g:eGFP::OCSter* cassette was excised from the pBJ36 plasmid using NotI restriction enzyme and ligated into the binary plasmid pMLBART for *chpin1* (10) plant transformation.

ChPIN1p::ChPIN1gS1,2,3A:eGFP. The construct *ChPIN1p::ChPIN1g:eGFP* in the pBJ36 plasmid was mutagenized by GenScript to generate *ChPIN1p::ChPIN1gS1,2,3A:eGFP*, in which the S1, S2 and S3 serines are replaced by alanines [TCG to GCT; (11)]. The *ChPIN1p::ChPIN1gS1,2,3A:eGFP* cassette was excised from the pBJ36 plasmid using NotI restriction enzyme and ligated into the binary plasmid pMLBART for *C. hirsuta chpin1* plant transformation.

ChCUC1p::ChWAG1. The *ChCUC1* promoter region (1921 bp) was PCR amplified using *C. hirsuta* Ox genomic DNA as the template, and ChCUC1p-PstI-F and ChCUC1p-XmaI-R as primers. The PCR product (1933 bp) was subcloned into pBJ36 and sequenced. The *ChWAG1* cassette was digested with XmaI/BamHI from pUC57-*attL1-Xma1-ChWAG1c-BamH1-attL2* (*ChWAG1* including stop codon), ligated into pBJ36-*ChCUC1p::OCSter*. The *ChCUC1p::ChWAG1:OCSter* cassette was excised from the pBJ36 plasmid using NotI restriction enzyme and ligated into the binary plasmid pMLHYG for *C. hirsuta chpid-1+/-;chwag1-1;chwag2-1* plant transformation.

Generation and verification of transgenic plants

Agrobacterium tumefaciens strain *GV3101* was cultivated in YEB medium at 28°C until reaching OD_{600nm} = 0.8-1 and then centrifuged at 3500 rpm. The cell pellet was then resuspended in infiltration medium including 2.3 g/L Murashige-Skoog (MS) basal salt mixture with vitamins (Duchefa, M0409), 5% sucrose (Carl Roth, CAS Nr. 57-50-1), 2 µg/L Benzylaminopurin (Sigma-Aldrich ref. B3274), and 0.05% (v/v) Silwet L77 (CAS-Nr.: 2730678-1). Plant transformations were performed using the floral dip method (12) and then kept in bags for 24 hours at room temperature in darkness before moving the plants to standard growing conditions.

To select for BASTA resistant T1 plants, the plants were grown on soil and sprayed with 0.2% BASTA (Bayer) solution after the cotyledons had expanded. To select for transgenic plants resistant to hygromycin, the T1 seeds were sterilized in a solution with 2.4% sodium hypochlorite and 1% Tween20 for 10 minutes, washed 3 times with sterile deionized water and resuspended in sterile 0.1% agarose. Seeds were then sowed on 1/2 MS agar plates (without sucrose) containing 50 µg/ml hygromycin (Roth CELLPURE, CAS Nr. 31282-04-9) for *C. hirsuta*. Plates were stratified at 4°C in the dark for one week for *C. hirsuta* before being transferred to the plant cultivation room. To select for transgenic plants harboring the RFP seed coat marker (13), seeds were inspected under a fluorescence stereomicroscope. Moderately fluorescent and strongly fluorescent seeds were selected.

For each construct, a minimum of fifteen independent lines were analyzed. The copy number of the transgenes was determined by IDna Genetics Ltd (Norwich, UK). For this, genomic DNA was extracted from the samples and assayed with duplicate multiplex PCRs for the transgene component and IPC (Internal Positive Control - this is a gene which is invariant in copy number and sequence in the material under study, against which the copy number of the transgene is measured). The number of transgene copies was estimated from the delta CT values of a RT-

qPCR. Copy number information was used to identify single-copy lines and to compare expressions between transgenes or backgrounds.

Generation of *chpid*, *chwag1*, and *chwag2* mutants using CRISPR/Cas9

The single guide RNAs (sgRNAs) targeting the coding sequences of *ChPID*, *ChWAG1*, and *ChWAG2* were designed using CCTop (https://crispr.cos.uniheidelberg.de/). Two sgRNAs (Fig. S12A) with low chance of causing off-target effects and high-efficiency scores were selected for each target gene. Fragments *attL5-AtU6p::sgRNA1-AtU6::sgRNA2-attL2* (containing the sgRNAs driven by the RNA polymerase III promoter *AtU6*) were synthesized by GenScript. Then, the plasmid was used in Multisite Gateway reactions with an *attL1-ECp::SpCas9-attR5* block (SpCas9 driven by a promoter active in the egg cell) and a pPZP200 backbone containing *attR1-ccdB-attR2_OLE1p::OLE1:RFP*. After transformation of *C. hirsuta* Ox plants, the gene-editing efficiency was estimated. For this, genomic DNA was extracted from the primary inflorescence of around 100 T1 plants. Then, the regions targeted by the sgRNAs were amplified and sequenced. The sequences were analyzed using the method Tracking of Indels by Decomposition (TIDE) analysis (http://shinyapps.datacurators.nl/tide/). T1 plants with approximately 100% or 50% editing efficiency were selected as homozygous and heterozygous mutants, respectively. The TIDE analysis was performed again with the T2 progeny plants to validate the zygosity. Finally, Cas9-free homozygous plants were selected in the T2 generation.

Analysis of macroscopic phenotypes

Creation of leaf silhouettes for downstream analyses: Leaves from 3-week-old plants were flattened against white paper using adhesive polyester film (VMR, 731-0311), and were then scanned at 800dpi using an EPSON PERFECTION v700 photo scanner. The images were then binarized using an appropriate grey value as threshold.

Quantification of leaf morphological parameters: Silhouettes of *A. thaliana* leaves and *C. hirsuta* terminal leaflets were analyzed using Leaf Interrogator (LeafI) software (https://gitlab.mpcdf.mpg.de/g-adamrunions/leafinterrogator_zhang_et_al) (14). Silhouettes were registered and converted into vector contours (sequences of 2D positions). Two common landmarks at the tip and base of the leaf blade were identified. Contours were resampled to have N-points between landmarks [(2N+2)-points total per contour, N=60], equally spaced along the contour. The Normalized difference margin complexity (NMDC) of each contour was calculated as (perimeter contour − perimeter convex hull) / (perimeter contour + perimeter convex hull) (15). Leaf shape-space plots were calculated as described in (14). The resampled contours were analyzed using Elliptical Fourier Descriptor (EFD). Translation and scale-invariant EFDs were calculated using a modified procedure that combines Procrustes-Based alignment with the EFD-based approach described by Kuhl and Giardina (16). Variation over the 2 first principal components was plotted. Ellipses indicate half a standard deviation of the mean for each group.

Dexamethasone induction of *ChCUC1* for confocal imaging

Twenty independent T2 lines carrying the *ChCUC1p::LhG4:GR; Op::ChCUC1:tdTomato* transgene were isolated in *A. thaliana* Col-0 *PIN1p::PIN1:GFP* background, and their phenotypes and transgene expression pattern were scored and compared to *ChCUC1p::ChCUC1g:Venus* lines. Based on this information, two representative lines were selected and used in experiments. Dexamethasone powder (Sigma Aldrich, D4902) was dissolved in DMSO (Carl Roth, 4720.1) to 50 mM and used to prepare a working solution containing 10 µM dexamethasone and 0.01% Triton X-100. The control solution was created by replacing the dexamethasone stock for DMSO. For confocal imaging, seedlings were grown in pots with soil until leaf 3 was visible, treated by spraying dexamethasone (induction) or mock solution (control), and cultivated for another 24 h before imaging. Plants were dissected by removing leaves until exposing leaf 4 at the target developmental stage, and mounted on 1.5% agar plates containing 1/2 MS (Duchefa Biochem, M0222.0050), 1% sucrose and 1 ml/L of Plant Preservative Mixture (PPM, Plant Cell Technology Inc., Washington DC, USA). Induction (dex) versus control (mock) treatments to evaluate the effect of ChCUC1 expression on PIN1:GFP polarity in the margin were performed 4 times. Induction (dex) versus control (mock) comparative analysis was performed between leaves showing very similar lengths (+dex: 306, 303, 289 µm; -dex: 336, 288, 317 µm) to reduce variability in PIN1:GFP polarity due to developmental stage.

Generation of ChCUC1 mosaics

Eighty independent transgenic T1 lines carrying the *35Sp::lox-spacer-lox::ChCUC1:V* construct were isolated in a *PIN1p::PIN1:GFP; HSp::dBox:Cre* background. Of these, 10 were verified to produce ChCUC1:Venus clones after a heat shock in roots and leaves. Two lines were studied further and generated the data described in the results section. The effect on PIN1:GFP was evaluated on 2 different types of experiments, either consisting on a single image after induction, which was repeated twice, or in another 2 experiments consisting on time-lapse imaging after induction.

Seeds were sown on STAR DISH sterile 100x15mm square Petri dishes (Phoenix Biomedical) containing MS medium [per 1 L: 10g sucrose (Sigma Aldrich, 84097), 4.3g Murashige and Skoog salts (Sigma Aldrich, M5524), 0.5g MES 2-(MN-morpholino)-ethane sulfonic acid (Carl Roth, 4256.4), 0.8 % BD Bacto™ Dehydrated Agar (Fisher Scientific, Product Code-10455513), 1 mL MS vitamins (Sigma, M3900)]. pH was adjusted to 5.7 with 1M KOH, and vitamins were added prior to pouring. After 48 hours of stratification, plates were moved to a growth chamber at 22°C and long day illumination. Plants were heat shocked 2.5 DAS (to obtain ChCUC1:Venus clones in the leaf margin of developing leaf 1) or 10-15 DAS (clones in the abaxial epidermis of adult leaves or sepals). Heat-shock induction was done by incubating the plates at 37°C for 1.5 hours. Samples were dissected 15-18 hours after heat-shock induction. In the case of leaf 1 margin clones, one cotyledon was dissected to detach it completely without splitting the hypocotyl, in order to expose the first two leaves (17). For clones in the abaxial epidermis, leaves were removed to expose the tissue of interest. Seedlings were moved to a

fresh plate containing vertical slits in the agar. The roots and the hypocotyls of the seedlings were gently pushed inside the slit to anchor them to the medium. For imaging, seedlings were then completely submerged under autoclaved water carrying 1 mL/L of PPM. The abaxial (ventral) side of the samples was imaged 15-18 hours after heat-shock induction and then every 24 hours to follow the ChCUC1:Venus clones for a variable number of days. After every image, plants were returned to the growth chamber. Part of the plants that were heat shocked 2.5 DAS were not dissected and imaged. Instead, they were cultivated for 2 weeks and fully-expanded leaves 1 and 2 were photographed to record their final morphology.

Confocal Scanning Laser Microscopy (CSLM)

CSLM images were obtained with a Leica SP8 upright instrument equipped with a long working distance water-immersion AP 25X/0.95 objective (both Leica Microsystems). GFP was excited using a 488 nm laser and the detector collection window was set to 493-510 nm. Venus was excited using a 514 nm laser and the detector collection window was set to 520-550 nm. tdTomato was excited using a 561 nm laser and the detector collection window was set to 575-600 nm. Chlorophyll emission was collected in the range 650-700 nm. When necessary, cell walls were stained with Propidium Iodide solution (10 mg/mL) for 15 min, washed in water, and then imaged immediately. Propidium Iodide was excited using a 561 nm laser and collection window was set to 600-650 nm. Laser intensity and detector gain were set to ensure the maximum intensity range and avoid signal saturation, except for cell boundary staining for cell segmentation, where controlled saturation is desirable. The XY pixel size was typically between 0.3 and 0.5 µm, whereas the Z step size ranged from 0.7 to 1 µm. Images were obtained with hybrid detectors (HyD), except for chlorophyll, which was recorded using a photomultiplier (PMT) detector. Images were stored with 12-bit or 16-bit channel intensity depth.

Generation of curved 2D meshes for downstream analyses

The curved 2D meshes that represent the surface of leaf confocal stacks were created with MorphographX version 2.0 using standard procedures (18, 19). The surface was calculated from either membrane signal (acyl:tdTomato expression or Propidium Iodide staining) or PIN1:GFP signal. The meshes were smoothened and then subdivided until the triangles were equal to the pixel size of the confocal images. Cell segmentations were also created from either membrane or PIN1:GFP signal. To do so, the signal was projected onto the mesh using the process 'Project Signal' with an appropriate depth range so as to obtain sharp boundaries of the epidermis cells. Subsequently, the process 'Auto Segmentation' was run (parameters: 'combine threshold' = 1.3, remaining ones with default values), after which the segmentation errors were corrected manually.

Analyses of PIN1:GFP polarity

Assessment of polarity: This was done along the epidermal plane (planar polarity) based on the distribution of GFP accumulation in the anticlinal plasma membranes of the cells. For 3D

images converted into MorphographX meshes, the samples were analyzed with the process 'Compute PIN orientation' (default parameters) to obtain the axis and magnitude of the PIN1:GFP signal anisotropy. Next, polarity (the direction towards which PIN1:GFP accumulation is highest) was estimated by inspecting the signal crescents that PIN1:GFP typically displays in the plasma membrane. This step was done by two researchers independently, and the results were compared to verify ChCUC1 effects.

Statistical comparison of polarity frequency distributions between groups: First, the main growth direction of the tissue was established as the reference direction (in most of the blade this direction follows the proximo-distal (PD) axis from the base to the tip of the leaf, and in the marginal outgrowths it is the direction from the base to the tip of the outgrowth). Next, a region of interest was defined in each leaf sample. This was typically the leaf margin undergoing patterning, the ChCUC1 domain in *C. hirsuta*, or ChCUC1:Venus clones and equivalent negative control clones in the case of the mosaic analyses. Then, each cell in the region of interest was assigned to a PIN1:GFP polarity class: 'apical' (GFP towards the reference direction), 'basal' (opposite to the reference direction), 'bipolar' (both apical and basal), 'lateral' (perpendicular to the reference direction), or 'non-polar' (isotropic GFP signal). When appropriate, additional classes were defined (e.g. 'apical-lateral'). Cells for which it was not possible to determine reliably the main direction of PIN1:GFP accumulation were classified as 'unknown'. Finally, the frequencies of each polarity class were calculated, and statistical comparison of groups was performed using Chi2 tests.

Quantification of tissue and cell parameters in MorphographX

Quantification of fluorescent reporter expression: Using MorphographX meshes with segmented cells, the signal from a confocal channel containing expression data was projected onto the mesh. The depth range was adjusted to isolate the signal from the epidermal cells. Next, the signal in each cell was computed with the process 'Heat Map: Signal Interior' using default parameters.

Measurement of cell growth and proliferation: First, two segmented meshes corresponding to different time points of a time-lapse imaging experiment, were loaded simultaneously in MorphographX. Next, each cell in the mesh of the subsequent time point was labeled with its corresponding mother cell from the mesh of the preceding time point. Growth was measured with the process 'Heat Map: Area change' and calculated as *[(area posterior time point / area anterior time point) - 1] × 100*. Proliferation was measured with the process 'Heat Map: Proliferation'.

Determination of the distance between the tip of a leaf margin protrusion and PIN1:GFP repolarization (Figure 2A-C): First, the summit or tip of a protrusion of interest was defined based on where epidermal PIN1 polarities converge and where the provascular PIN1 expression connects with the epidermis. Then, in MorphographX, the mesh vertex corresponding to the tip of the protrusion was selected. Subsequently, the distance of each cell from the tip was calculated using the 'Cell Distance' process (Wall Weights = Euclidean). Finally, the distance

from the protrusion tip to polarity reversal was calculated by averaging the distances of the first 5 cells with clear reversed polarity. All leaves were at the same developmental stage, which was inferred from their length.

Measurement of the leaf length and quantification of auxin activity maxima in Col-0 and ChCUC1g:Venus carrying DR5v2::NLS:TdTomato (Figure S5): Leaf 6 was imaged at different developmental stages in the confocal microscope. The length of each imaged leaf was measured in MorphographX by adapting a Bezier curve originating from the leaf base and aligned with the curved leaf surface along the midrib up to the leaf tip using multiple control points. The number of auxin activity maxima was determined by counting margin fluorescence foci in the tdTomato channel, excluding the foci at the tips of leaves.

Parameters measured in MorphographX were exported as tabular data in CSV format for downstream statistical analysis and plotting.

Dexamethasone induction of *ChCUC1* for transcriptomic analysis

For transcriptomic analysis, 12-days-old seedlings harboring *ChRCOp::LhG4:GR; Op::ChCUC1:Venus* were incubated while shaking in liquid MS medium with 10 µM dexamethasone (induction) or 0.1% DMSO mock solution (control). Seedlings without cotyledons, roots, and mature leaves were harvested at 2, 4, 6, and 8 hours after treatment. For each replicate, twenty seedlings were collected for RNA isolation. Three independent replicates treated with dexamethasone were used as the experimental group, and three independent replicates treated with DMSO mock solution were used as the control group.

RNA extraction, quantitative RT-PCR, and transcriptomic analysis

Total RNA was extracted from 12-day-old seedlings (the cotyledons, mature leaves, hypocotyls, roots were removed, and the shoot apices comprising developing leaves were collected) using the RNeasy Plant Mini Kit (Qiagen 74904). DNase I treatment was conducted on-column during the RNA extraction by RNase-Free DNase Set (Qiagen 79254). Three biological replicates for each treatment or genotype were used. For quantitative RT-PCR, 1µg of total RNA was reverse-transcribed using the SuperScript™ VILO™ cDNA Synthesis Kit (Invitrogen 11754050). Quantitative PCR was performed using *Power* SYBR™ Green PCR Master Mix (ThermoFisher Scientific 4367659) in QuantStudio 3 (96 wells) or QuantStudio 5 (384 wells) (ThermoFisher Scientific). Relative expression levels were calculated by the comparative CT (ΔΔCT) method (20), using *ChUBQ10* or *ChTIP41* for normalization. Oligonucleotide primers used for qRT-PCR are listed in Table S4. For transcriptomic analysis, library construction and subsequent sequencing were performed at the Max Planck-Genome-Centre Cologne (https://mpgc.mpipz.mpg.de/home/). The RNA integrity was first examined using an Agilent 2100 Bioanalyzer II. 1µg of total RNA was used for library preparation following polyA mRNA workflow using NEBNext® Ultra™ II Directional RNA Library Prep Kit for Illumina® (E7760L), and libraries were sequenced on Hi-Seq2500 System (Illumina)

to produce approximately 21 million single-end 150bp reads per sample. RNA-seq data have been deposited at the Gene Expression Omnibus (GEO, GSE241051).

Chromatin Immunoprecipitation sequencing (ChIP-seq)

ChIP-seq was performed as described in Maximized Objects for Better Enrichment (MOBE)-ChIP (21) with some minor modifications. *C. hirsuta ChCUC1p::ChCUC1g:Venus* plants grown on soil in short-day conditions were harvested fourteen days after germination. For crosslinking, for each replicate, about 20 g of seedlings (without root and mature leaves) were crosslinked with 1% formaldehyde for 20 min at 4°C. For chromatin extraction and shearing, each 2-3 g of pulverized tissue (tissue ground with pre-cooled mortar and pestle) was resuspended in 45 mL of extraction buffer 1. The solutions were filtered through double hydrophilic nylon membranes (pore size 60 µm plus 20 µm, from Millipore), then centrifuged for 20 min at 1600 *g* at 4°C. The pellets were resuspended and washed in 20 mL extraction buffer 2 three times and centrifuged for 10 min at 1600 *g* at 4°C in between each resuspension/wash cycle, followed by last resuspension and wash in extraction buffer 3, and centrifuging for 10 min at 1600 *g* at 4°C. The pellets were resuspended in 500 µL of nuclei lysis buffer, transferred to 1.5 mL microcentrifuge tubes, and incubated for 35 min at 4°C with gentle rotation at 10 rpm. After dividing each chromatin solution sample into single aliquots of 250 µL, the chromatin was sheared to 200-500 bp fragments by using a Bioruptor® Pico sonicator (Diagenode) for 8 cycles (30 sec on/30 sec off) at 4°C. Aliquots from each sonicated chromatin sample were combined into a 2 mL microcentrifuge tube, centrifuged for 15 min at full speed at 4°C, and then flash-frozen in liquid nitrogen (10 µL of supernatant as input from each sample). For chromatin immunoprecipitation, each sonicated chromatin solution was diluted ten times with ChIP dilution buffer in 50 mL Falcon tubes and incubated with 16 µL Anti-GFP antibody (ab290, abcam) overnight at 4°C with gentle rotation at 10 rpm. Then 400 µL of pre-washed Dynabeads™ Protein A were added to each chromatin-antibody mixture sample and incubate for 1h at 4°C with gentle rotation at 10 rpm. The magnetic beads were captured with a magnetic stand (DynaMag™-2 magnet). The magnetic beads were washed successively with the following washing buffers 4x2 times: Low salt wash buffer, High salt wash buffer, LiCl wash buffer, and TE buffer. The captured protein-DNA complexes were eluted with 0.5 mL ChIP elution buffer in 1.5 mL microcentrifuge tubes and incubated at 65°C with agitated shaking at 1300 rpm for 40 min. The tubes were centrifuged briefly followed by magnetic separation for supernatant collection. For reverse crosslinking and purification of DNA, the eluates were incubated overnight at 65°C with the presence of 0.2M NaCl, followed by treatment of 15 µL of proteinase K (12 units or 300 µg, New England Biolabs P8107S) for 3h at 45°C. The DNA was further purified by ChIP DNA Clean & Concentrator kit (ZYMO Research, D5205). The precipitated DNA was eluted in 25 µL H2O and then stored at -80°C. The input DNA samples were purified in the same manner as precipitated DNA samples. For sequencing, library construction and subsequent sequencing was performed at the Max Planck-Genome-Centre Cologne (https://mpgc.mpipz.mpg.de/home/). The Low input ChIP-seq libraries were constructed using the NEBNext® Ultra™ DNA Library Prep Kit for Illumina® (E7370). The libraries from three biological replicates of case, control, and input samples, respectively, were sequenced on Hi-Seq3000 System (Illumina) to produce 22-24 million

single-end 150bp reads per sample (3GB each replicate). ChIP-seq data have been deposited at the Gene Expression Omnibus (GEO, GSE242999).

DNA Affinity Purification sequencing (DAP-seq)

DAP-seq was performed as previously described in (22). Briefly, the *C. hirsuta* genomic DNA was extracted from rosette leaves of 4-week-old plants by Qiagen DNeasy Plant Maxi Kit (Cat No. 68163). For DNA library preparation, about 5 µg genomic DNA was first sheared by a Bioruptor® Pico sonicator (Diagenode) for 6 cycles (30 sec on/30 sec off) at 4°C to 200-400 bp fragments, followed by end repair, A-tail reaction, and adaptor ligation processes. For protein expression, 1.5 µg of pIX-Halo-ChCUC1 or empty pIX-Halo plasmid DNA was used to express Halo-ChCUC1 or Halo protein (negative control) *in vitro* by using the TNT SP6 Coupled Wheat Germ Extract System (Promega, L4130). For protein binding, the Halo-ChCUC1 proteins or Halo proteins were incubated with the Magne HaloTag beads (Promega, G7282) at room temperature for one hour. The protein-bead conjugates were then washed three times and incubated with the genomic DNA library at room temperature for one hour. The bound DNA fragments were recovered and PCR-amplified, followed by size selection (200-400bp). Finally, the purified enriched DNA libraries were sequenced to a depth of 22-24 million single-end 150bp reads (3GB each replicate) by Illumina HiSeq3000 at the Max Planck-Genome-Centre Cologne (https://mpgc.mpipz.mpg.de/home/). Three biological replicates were performed in the DAP-seq experiments. DAP-seq data have been deposited at the Gene Expression Omnibus (GEO, GSE241208).

Transcriptome sequencing data analysis

Single-end reads were aligned to the *C. hirsuta* reference genome (CHIV1, http://chi.mpipz.mpg.de/) using STAR v2.4.2a with default parameters. Raw read counts per gene were quantified with HTSeq v0.5.4p1 (https://www-huber.embl.de/users/anders/HTSeq/) using the "--stranded=no --type=CDS'' option. Differential expressed genes between the dexamethasone-treated and mock-treated samples were determined using DESeq (23). We found the most sensitive parameter settings for the function estimateDispersions were method="blind", and sharingMode="fitonly" (24). Gene ontology (GO) analysis of *ChCUC1* downstream responsive genes was performed by converting the *C. hirsuta* gene IDs into their *A. thaliana* orthologue gene IDs, followed by analysis by enrichGO function in the ClusterProfiler package (25) in R.

ChIP-Seq and DAP-seq data analysis

Quality control, read mapping, and peak calling for the DAP-seq and ChIP-seq analyses were conducted with the ENCODE Uniform processing pipeline for ChIP-seq (https://www.encodeproject.org/chip-seq/transcription_factor/). Bowtie and MACS2 were selected as mapper and peak caller, respectively, and only IDR-optimal peaks were used. Peaks were called for case and mock libraries separately, using their respective inputs, and mock peaks were then subtracted from the case peaks using bedtools. Signal and peak files were visualized

and inspected using a local instance of the UCSC genome browser (https://ucscbrowser.mpipz.mpg.de/). The enriched binding motives were identified by MEME-ChIP (http://meme-suite.org/tools/meme-chip). The visualization of distribution of ChCUC1-binding peaks from ChIP-seq and DAP-seq was performed by the R package ChIPseeker (26). Gene ontology (GO) analysis of genes bound by ChCUC1 was performed using the enrichGO function in the clusterProfiler package (25) in R.

Replication, statistical analyses, plotting

Statistical analysis was performed in R (R version 3.6.3) and Python3. The statistical tests and numbers of replicates are indicated in the figure legends. The significance threshold used was P values <0.05. For multiple-pairwise comparisons, Kruskal-Wallis followed by Dunn's *post hoc* test was used as non-parametric test (Figures 1I, 4B; Supplementary figures 13B,D, 14B, 16F, 17C). Comparisons between two groups were performed by two-tailed unpaired Student's t test, assuming equal variances (with n.s., not significant; *** $p < 0.001$; ** $p < 0.01$; * $p < 0.05$) for Figures 2C, 3D,H, and Supplementary figure 15D. To determine whether the distributions of PIN polarities in two samples were different, the Chi-square test of independence was used (Figures 2F, 4K). For enrichment analysis, p-values were calculated by Fisher's exact test (Figures 3E). To compare the expression of *ChCUC1:V* and *ChWAG1:tdT* (Figure 4F), a regression analysis was performed using a linear model.

Description of the computational models

We implemented computational models to understand how the molecular interactions between auxin, the auxin efflux transporter PIN1, and the transcription factor CUC1 might produce emergent polarity patterns in a 2D tissue. For brevity, in the following description, we denote these two proteins as PIN and CUC. To represent the epidermis in a region encompassing the margin of the leaf primordium we use a 2D grid of cells (Display item 1A). Accordingly, the central columns correspond to marginal cells and the columns to the left or right side of this central domain correspond, respectively, to the adaxial and abaxial epidermal cells. Some cellular processes (e.g. expression of a gene) are modulated by two identity factors: margin identity (middle domain, *MD*), which is highest in the central column of cells (Display item 1B), and base identity (proximal, *PROX*), which is highest at the bottom-most row of cells and decreases towards the tip (Display item 1C). To simplify simulations, all cells are squares of unit area, and cell walls are of unit length. In addition to the identity factors, each cell stores a concentration of auxin and CUC, as well as the total amount of PIN in the cell. Each cell wall stores the amount of PIN allocated to each side of the cell (Display item 1D). As in previous models in the literature (27, 28), we assume that the extracellular space can be reasonably approximated by direct cell-to-cell transport of auxin (29).

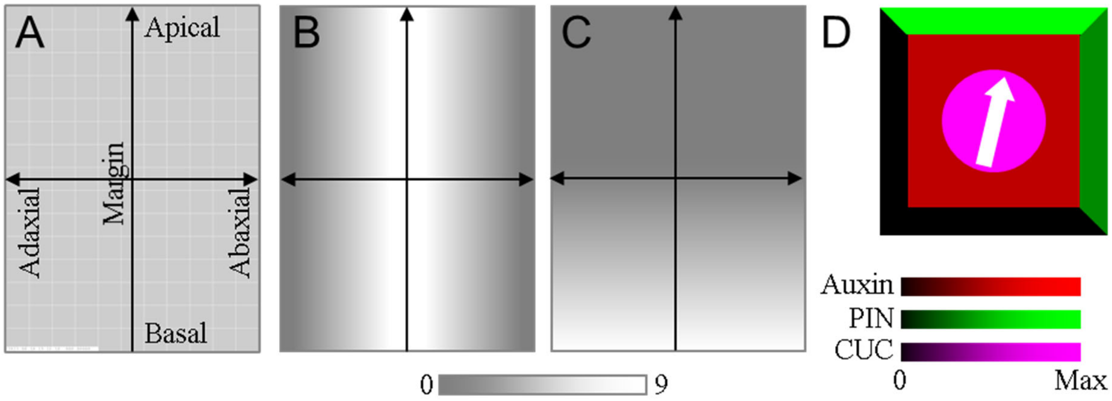

Display item 1. General characteristics of the models. (A) Correspondence between the cells in the model canvas and the axes of a developing leaf. The central cell columns correspond to the leaf margin. (B) Distribution of the middle domain identity factor *MD* on the model canvas. (C) Distribution of the proximal identity factor *PROX* on the model canvas. (D) Graphical representation of a model cell, including the auxin concentration (red central square), the CUC concentration (magenta circle), the PIN amount on each of the four cell faces (four green trapezes), and the direction of net PIN-mediated auxin transport through the cell (white arrow).

The amount of PIN transporters in any given cell face changes over time, and is governed by an auxin-dependent feedback. Our experimental results indicate that the input of CUC to PIN polarity during patterning is instructive (Figure 2), and that this is at least partially via promoting WAG-mediated PIN phosphorylation (Figures 3 and 4). Consequently, we model PIN as the sum of two fractions, corresponding to unphosphorylated and phosphorylated molecules, and assume that CUC promotes PIN phosphorylation, and therefore regulates the relative balance between the two fractions. We explore two model variants, which differ only in the effect of phosphorylation on PIN function. We consider these two specific variants

because they correspond to the two proposed modes by which phosphorylation affects PIN function: by increasing its transport activity (30-32) or by modifying its membrane localization (11, 33-35). In the first model, termed the *PIN efficiency modulation model*, PIN is allocated to the cell wall based on the auxin concentration in the neighboring cell [up-the-gradient hypothesis, UTG; (27, 28)]. In contrast to previous UTG models, where the transport efficiency of PIN is constant between cells, here we assume that the phosphorylated PIN fraction has a higher transport efficiency. In the second model, termed the *PIN polarization modulation model*, the unphosphorylated and phosphorylated PIN fractions are governed by different polarity cues: the former fraction polarizes based on the auxin concentration in the neighbors (UTG), whereas the latter fraction follows auxin flow [with-the-flux hypothesis, WTF; (36-39)].

The models are implemented in Python and the rate equations are solved using the function *scipy.intergrate.odeint* with default parameters, except for those representing cell to cell auxin movement, which are solved using the forward Euler method. The values used for each parameter are listed in Table S5, at the end of this document. The code is available at https://github.com/davidwilson-85/2Dmodel_Auxin_PIN_CUC.

Description of the model equations

Each cell i stores an auxin concentration A_i that changes due to production, degradation and the net movement of auxin between neighboring cells, as described by the equation:

$$\frac{dA_i}{dt} = k_{AS} - k_{AT}A_i - \sum_j \Phi_{i\to j} , \quad \text{(Eq. 1)}$$

where k_{AS} is the basal auxin synthesis rate, k_{AT} is the rate of auxin degradation, and the sum $\Sigma_j \Phi_{i\to j}$ captures changes in concentration due to the movement of auxin between cell i and its four neighbor cells j. The flux from cell i to cell j is denoted $\Phi_{i\to j}$, and has the following form:

$$\Phi_{i\to j} = T_i A_i PIN_{i\to j} - T_j A_j PIN_{j\to i} + k_D(A_i - A_j) . \quad \text{(Eq. 2)}$$

Here the coefficients T_i and T_j capture the transport efficiency of PIN in cells i and j, respectively, and k_D is the auxin diffusion rate. The auxin concentration in cells representing perfect sources or sinks is fixed at a constant prescribed value. All simulations implement a perfect auxin source in the cell at the second row and 6th column of the template, which represents the tip of the leaf, denoted as $A_{(6,2)}$.

Epidermal CUC expression during patterning of the first margin protrusion is visible in the margin but excluded from the base and the tip [Figure S15A; (40)]. To model this expression pattern, CUC production is promoted by the middle domain identity factor *MD*, and inhibited by auxin and the proximal identity factor *Prox*. Accordingly, the CUC concentration in cell i changes following the rate equation:

$$\frac{dCUC_i}{dt} = k_{MDC}MD_i - k_{AC}A_i\, CUC_i - k_{PC}Prox_i\, CUC_i - k_{CT}CUC_i , \quad \text{(Eq. 3)}$$

where k_{MDC} controls the strength of the middle domain identity factor *MD* dependent CUC creation, the k_{AC} and k_{PC} coefficients represent, respectively, the inhibitory effect of auxin and *Prox* on CUC concentration, and k_{CT} is the basal CUC turnover rate.

In leaves, patterning of auxin maxima and outgrowths is confined to the margin, where PIN1 expression is high (40). Within the margin, the highest PIN1 levels coincide with emerging protrusions and with CUC expression (Figure 1J-M). To model this expression pattern, the PIN creation rate is positively influenced by auxin and CUC:

$$\frac{dPIN_i}{dt} = k_{PS} + k_{AP}A_i + k_{CP}CUC_i - k_{PT}PIN_i \,. \qquad \text{(Eq. 4)}$$

Here, k_{PS} and k_{PT} are respectively the PIN synthesis and turnover rates. The coefficients k_{AP} and k_{CP} control the effect of auxin and CUC on PIN expression, respectively.

We assume that the proportion of the phosphorylated and unphosphorylated PIN species in a cell depends on the balance between PIN phosphatase and kinase activities, and that there is a constant dephosphorylation rate while phosphorylation is promoted by CUC (Figures 3 and 4). Assuming the fraction is in quasi-steady state, the proportion of phosphorylated and unphosphorylated PIN in a cell is given by:

$$PIN_{i_u} = \frac{{k_{0.5}}^H}{{k_{0.5}}^H + {CUC_i}^H} PIN_i \,, \; PIN_{i_p} = \frac{{CUC_i}^H}{{k_{0.5}}^H + {CUC_i}^H} PIN_i \,. \qquad \text{(Eq. 5)}$$

In these equations, PIN_{i_u} and PIN_{i_p} are the concentrations of unphosphorylated and phosphorylated PIN molecules in the cell *i*, respectively. The constant $k_{0.5}$ controls the relative balance between phosphorylation and dephosphorylation, *H* (Hill coefficient) modulates the response of PIN to CUC, and CUC_i denotes the concentration of CUC in cell *i*. These equations assume that phosphorylation status changes quickly compared to the production and turnover of PIN proteins.

In the *PIN efficiency modulation model*, unphosphorylated and phosphorylated PIN have low (T_{basal}) and high (T_p) transport efficiencies, respectively (30-32), and therefore T_i depends on the proportion of each molecular species in a cell, computed with the expression:

$$T_i = T_{basal} \frac{PIN_{i_u}}{PIN_i} + T_p \frac{PIN_{i_p}}{PIN_i} \,. \qquad \text{(Eq. 6)}$$

In the *PIN polarization modulation model*, the transport efficiency of PIN is the same for all cells (i.e. $T_i = T_j = T_{basal}$).

In cells where PIN polarizes UTG, its polar localization is simulated using equations previously employed for phyllotaxis and leaf margin patterning (27, 28). The amount of PIN in cell *i* on the cell edge facing cell *j* is denoted PIN_{ij}, and determined by the equation:

$$PIN_{ij} = PIN_i \frac{{b_i}^{A_j}}{\sum_k {b_i}^{A_k}}, \quad \text{(Eq. 7)}$$

where PIN_i is the total amount of PIN available to be distributed between the walls of cell i, A_j is the concentration of auxin in the neighbor j, and A_k is the auxin concentration in the neighbor k. The exponentiation base b_i controls the degree by which differences in auxin amount between the neighbors polarize PIN at the cell edges of cell i. b_i is scaled from 1 to b_{max} based on the amount of middle domain identity in cell i, MD_i, in order to suppress the formation of convergence points outside the margin. Accordingly, b_i is computed with the formula:

$$b_i = 1 + \frac{b_{max} - 1}{MD_{max} - MD_{min}} (MD_i - MD_{min}). \quad \text{(Eq. 8)}$$

Here, b_{max} is the maximum sensitivity of PIN to differences in auxin concentration in neighbors, and MD_{max} and MD_{min} denote the limit values of the middle domain identity factor MD.

In the *PIN polarization modulation model*, the phosphorylated and unphosphorylated fractions of PIN polarize independently. This idea was explored previously by Bayer *et al.* (41), who presented a model that switched between polarization modes based on cellular auxin concentrations. Our data is consistent with a similar transition controlled by CUC. In each cell, the proportions of phosphorylated and unphosphorylated PIN are calculated using equation (5). Next, the unphosphorylated fraction is assumed to polarize following equation (7), whereas the phosphorylated fraction polarizes with the auxin flux as described below.

UTG models have traditionally assumed fast PIN allocation and therefore used quasi-steady state equations (27, 28, 42), whereas WTF models opted for rate of change equations (31, 32, 36-38, 43, 44) Thus, to combine both polarization mechanisms in the same cell, similar to Bayer *et al.* (41), each cell stores an intermediary factor F_{ij} on each face that controls the strength by which the membrane of cell i abutting cell j attracts phosphorylated PIN. Its production is promoted by auxin efflux and therefore we model its behavior using the WTF model equations presented in Rolland-Lagan and Prusinkiewicz (38) and also used in Abley *et al.* (45):

$$\frac{dF_{ij}}{dt} = \begin{cases} \alpha\, \Phi_{i\to j} - \gamma\, F_{ij}, & if\ \Phi_{i\to j} \geq 0 \\ -\gamma\, F_{ij}, & if\ \Phi_{i\to j} < 0 \end{cases}. \quad \text{(Eq. 9)}$$

Here, α determines the strength by which net auxin efflux from cell i to j promotes F_{ij} accumulation, and γ is the turnover rate. It is important to note that, according to equation (2), auxin movement from cell i to j is considered to be positive, whereas movement in the opposite direction is negative.

The amount of PIN in cell i on the cell edge facing cell j is computed as the sum of the unphosphorylated and phosphorylated fractions, as described by the equation:

$$PIN_{ij} = PIN_{i_u} \frac{b_i^{A_j}}{\sum_k b_k^{A_k}} + PIN_{i_p} \frac{c^{F_{ij}}}{\sum_k c^{F_{ik}}}, \quad \text{(Eq. 10)}$$

where the exponentiation base c controls the strength by which differences in F concentration between cell faces polarizes PIN.

The white arrows in the model visualizations represent the direction of net PIN-mediated auxin transport in each cell. To compute their direction, the amount of PIN at each side of the 4 cell faces are first converted to vectors with directions based on transport orientation. The PIN values of the cell faces that lie at the edge of the canvas are considered null. Subsequently, the cell vector is calculated as the sum of the eight face vectors.

Parameter values and stability analysis

To choose parameter values, we first simulated the simpler scenario of a *cuc* null mutant (for example, an *Arabidopsis thaliana cuc2-1* leaf margin): a leaf margin with a distal auxin maximum and an associated PIN polarity convergence, but without margin patterning. In UTG models that simply include auxin and PIN, polarity reversals are an intrinsic emergent property, and the wavelength between convergence points can be modulated by regulating the rate of PIN transport (29). Therefore, we model *cuc* mutants by setting a basal PIN transport efficiency (T_{basal}) value so that the wavelength between reversals is greater than the proximodistal length of the tissue template.

Next, we explored values for the parameters that determine CUC expression. We chose values such that the resultant CUC domain resembles the one observed for ChCUC1 in the epidermal cells from 3D confocal stacks of *C. hirsuta* leaves at the developmental stage where the first margin auxin site forms (Figure S15A).

In the *PIN efficiency modulation model*, we chose a value for T_{pho} (transport efficiency of phosphorylated PIN) that is around 10 times higher than T_{basal}. This ratio is based on PIN activity measurements performed *in vivo* using sea urchin oocytes after incubation with the PIN kinase PINOID (32).

In order to compare the two models explored here in a fair manner, we attempted to use very similar or identical values for all common parameters. To compare the models' robustness to noise, we adjusted parameters so that the shape and magnitude of the emergent auxin maximum was comparable. This helped to both ensure that models produced more comparable outputs and minimize the effect of overall auxin levels on stability when noise is applied to the model. We tested the effect of introducing random noise into auxin and CUC concentrations.

Noise is introduced into simulations by randomly perturbing either the auxin or CUC concentrations of each cell at the end of each simulation cycle. In the case of auxin, noise is added to cell i using the formula:

$$A_i = A_i + \theta_A, \qquad \text{(Eq. 11)}$$

$$\theta_A \in [-\varepsilon_A \frac{A_i}{100}, \varepsilon_A \frac{A_i}{100}], \qquad \text{(Eq. 12)}$$

where θ_A is a random number drawn from a uniform distribution in the range defined by the lower and upper limit, ε_A. CUC noise is added to cell i in an equivalent manner:

$$CUC_i = CUC_i + \theta_C, \qquad \text{(Eq. 13)}$$

$$\theta_C \in [-\varepsilon_C \frac{CUC_i}{100}, \varepsilon_C \frac{CUC_i}{100}], \qquad \text{(Eq. 14)}$$

where θ_C is a random number drawn from a uniform distribution in the range defined by the lower and upper limit, ε_C.

To analyze the output of the noise tests, we characterized the emergent auxin maxima along the central column of cells. The position and height of the maxima were calculated using the Python function *scipy.signal.find_peaks(height=30, prominence=20)*. Maxima with position < 4 (4th cell row) were filtered out, as were considered to belong to the preexisting distal maxima. If multiple emergent maxima were detected in a simulation replicate, the one with the highest amplitude was selected.

We tested different numerical integration time steps (*dt*) and found that in the range 0.1 - 0.001 the simulation outputs were comparable (Display item 2), and thus concluded that results obtained using *dt* = 0.1 are representative of the model's behaviour. This was true for the two model variants presented here. Consequently, tests for robustness to noise were performed using *dt* = 0.1 to be able to obtain enough replicates within a reasonable amount of time.

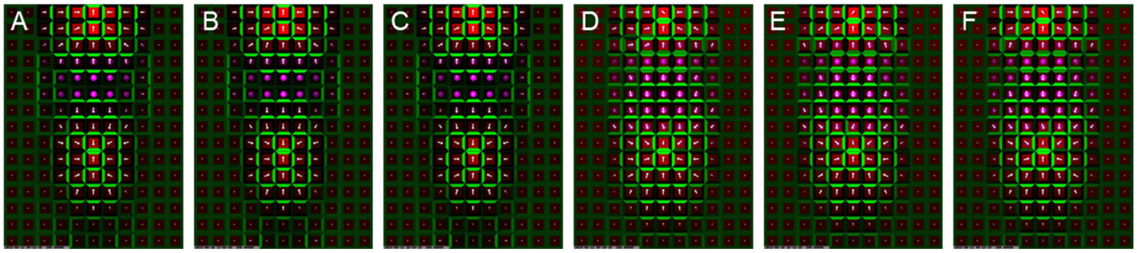

Display item 2. Output of the models using different integration time steps. (A-C) PIN efficiency modulation model. (D-F) PIN polarization modulation model. (A, D) *dt* = 0.1. (B, E) *dt* = 0.01. (C, F) *dt* = 0.001.

Supplementary figures

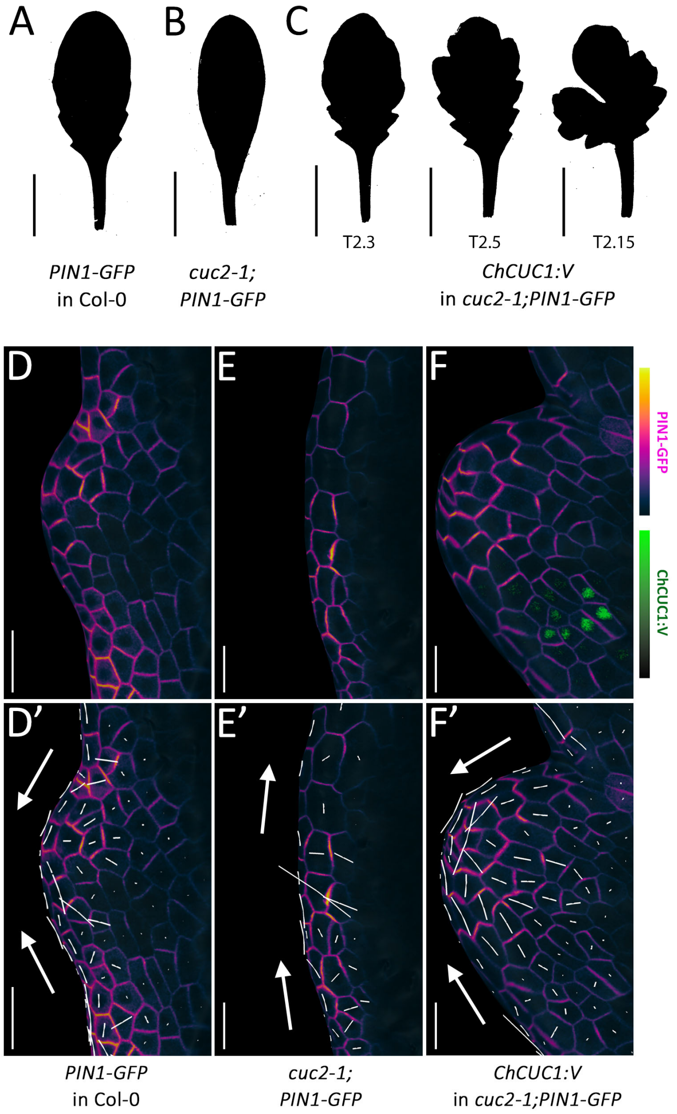

Figure S1. *ChCUC1* can replace *CUC2* to restore PIN1:GFP convergences and protrusion formation in *Arabidopsis thaliana*.

(A-C) Morphology of rosette leaf 8 of *A. thaliana* Col-0 carrying *PIN1:GFP (PIN1p::PIN1:GFP)* (A), *cuc2-1;* carrying *PIN1:GFP* (B), and *cuc2-1* carrying *ChCUC1:V* (*ChCUC1p::ChCUC1g:Venus*) and *PIN1:GFP* transgenes (C). Three independent T2 lines (T2. 3, T2.5, and T2.15) that are representative of the whole phenotypic spectrum obtained are shown.

(D-F') PIN1:GFP localization (D, E, F) and ChCUC1:V expression (F) in *A. thaliana* leaf 5 from the indicated genotypes. Shown are MorphographX surface meshes with the epidermal PIN1:GFP (purple-yellow) and ChCUC1:Venus (green) signals projected. (D', E', F') Same images as in (D, E, F) with overlaid polarity information. The white lines indicate PIN1:GFP orientation (line axis) and the degree of anisotropy (line length). The arrows indicate the overall PIN1:GFP polarity direction in the margin cells. *n* = 5 leaves (A-C). *n* = 3 samples (D-F').
Scale bars: 1 cm (A-C), 20 µm (D-F).

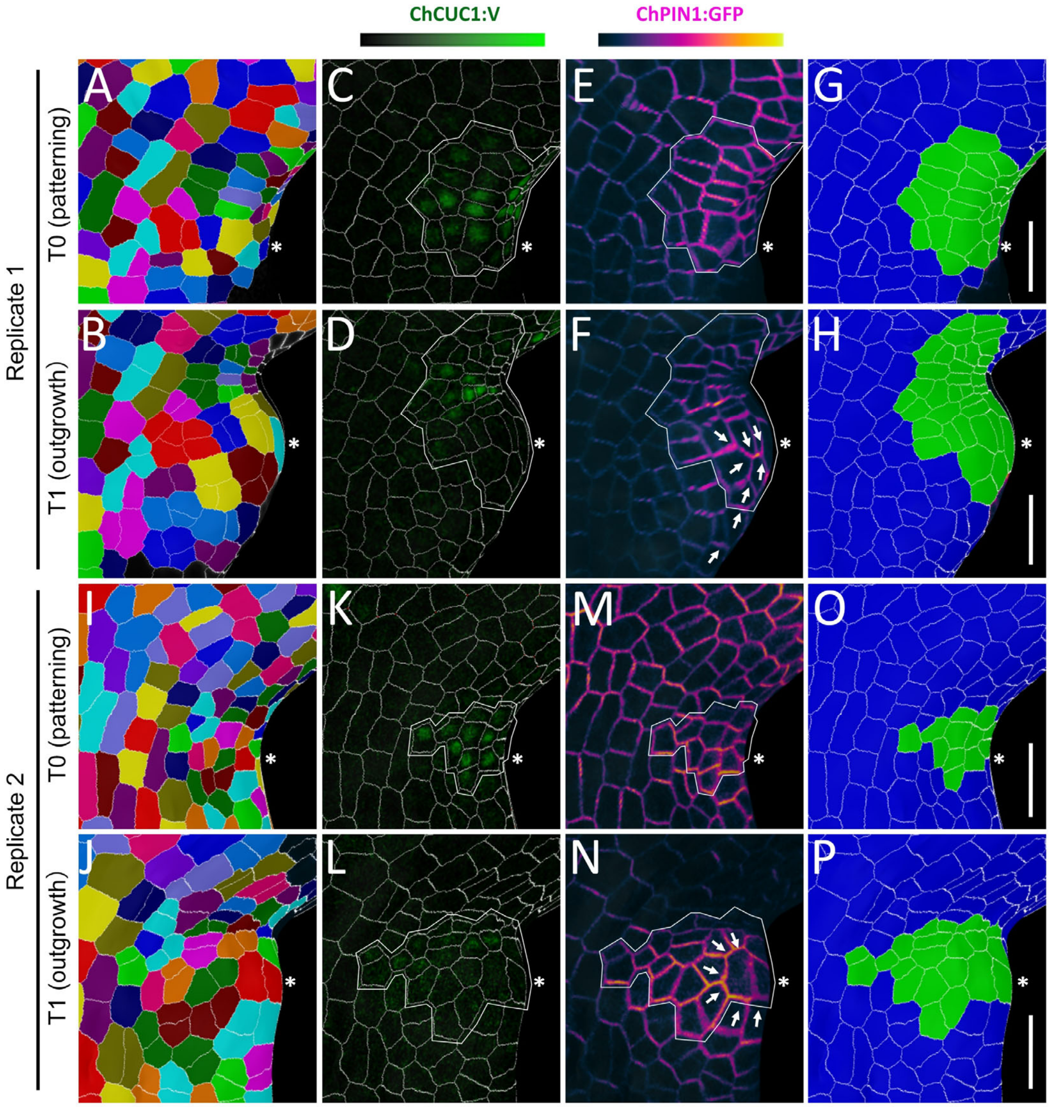

Figure S2. Fate mapping analysis of the ChCUC1 domain during lateral leaflet development in *Cardamine hirsuta* leaves.

The margin region of *C. hirsuta* leaf 5 expressing *ChPIN1:GFP* (*ChPIN1p::ChPIN1g:eGFP*) and *ChCUC1:V* (*ChCUC1p::ChCUC1g:Venus*) was subjected to time-lapse confocal imaging for capturing the patterning (T0 = 0h) and emergence (T1 = 24h) stages of the first lateral leaflet. The images were converted to cell-segmented MorphographX meshes in order to compute cell lineages and analyze the spatial relationship between the epidermal expression of *ChPIN1:GFP* and *ChCUC1:V*. Two replicates are shown (A-H; replicate 1 and I-P; replicate 2).

(A-B) Cell fate maps of the first replicate. The colors indicate the cell lineages. The asterisks mark the position of the ChPIN1 polarity convergence point, as calculated in T1 from (F), and mapped to T0 through lineage tracing. The asterisk allows comparing the ChCUC1 domain at T0 and the position of the ChPIN1 convergence point at T1.

(C-D) Projected epidermal ChCUC1:V signal. The white outline encircles the ChCUC1 domain and was calculated in MorphographX based on an arbitrary signal threshold on (C) and lineage-mapped to (D). Note the clearing of the ChCUC1:V signal in (D).

(E-F) Projected epidermal ChPIN1:GFP signal. The white arrows indicate the predominant direction of ChPIN1:GFP accumulation. Note cells forming PIN1 convergences are derived from the proximal ChCUC1 domain and they additionally recruit more proximal non ChCUC1:V-expressing cells (white arrows towards to the convergence point which are outside of the white outline).
(G-H) Cell lineage map of the cells constituting ChCUC1:V domain (green) and the non-ChCUC1:V domain (blue).
(I-P) Second replicate sample, shown using the same layout as (A-H).
Scale bars: 20 µm. *n* = 3 leaves.

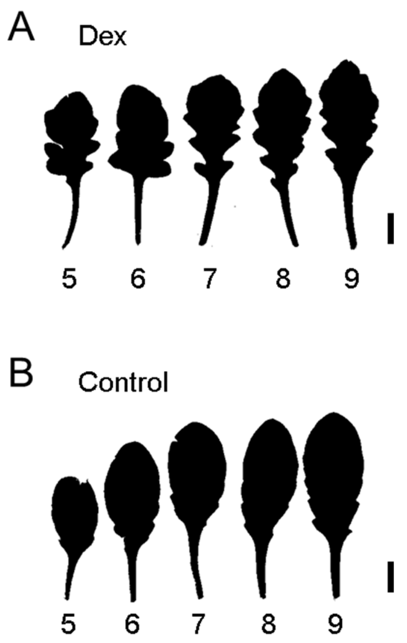

Figure S3. Leaf phenotype caused by sustained induction of *ChCUC1p::LhG4:GR; Op::ChCUC1:tdTomato* in *Arabidopsis thaliana*.

(A-B) Silhouettes of fully-expanded leaves from the *A. thaliana* strain *ChCUC1p::LhG4:GR; Op::ChCUC1:tdTomato; PIN1p::PIN1:GFP* treated with 10 µM dexamethasone (A) or mock (control) (B) solutions. The phenotypes indicate that the construct can increase leaf complexity and therefore is functional, and that it is only expressed after exposure to the inducer (dexamethasone). The numbers indicate the rosette leaf nodes. Plants were sprayed 7 days after sowing and then every 2 days until bolting. *n* (dex) = 1; *n* (control) = 7. Scale bars: 1 cm.

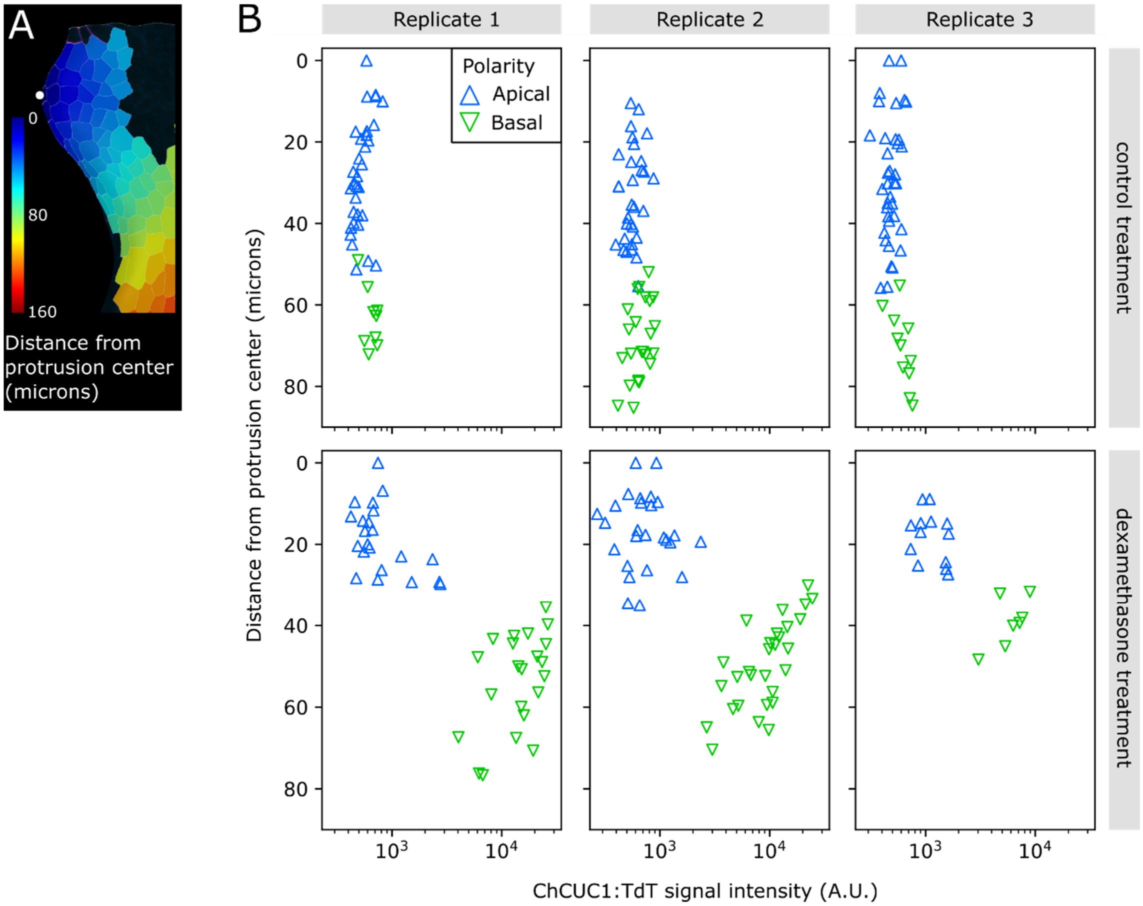

Figure S4. Effect of *ChCUC1p::LhG4:GR; Op::ChCUC1:tdT* on PIN1:GFP polarity in the *Arabidopsis thaliana* leaf margin.

(A) Procedure to classify the margin epidermal cells according to their position relative to an existing distal margin protrusion (polarity convergence point) to study the effect of ChCUC1 on PIN1:GFP polarity reversal. Shown is a MorphographX curved mesh of the epidermis of the margin of a leaf with a developing protrusion. The polygons indicate the cell segmentation. The heatmap indicates the Euclidean distance from the tip of the protrusion (white dot).

(B) Cell-level characterization of PIN1:GFP polarity in leaf 4 from *A. thaliana ChCUC1p::LhG4:GR; Op::ChCUC1:tdTomato* 24h after treatment with control or dexamethasone solutions. Each marker represents an epidermal cell, where the symbol/color indicates the PIN1:GFP polarity direction, the X axis show the ChCUC1:tdT expression, and the Y axis show the distance from the tip of the preexisting polarity convergence point (protrusion tip). Note how PIN1:GFP polarity consistently changes when a cell expresses ChCUC1:tdT. Note also that the X axis is log10 scaled.

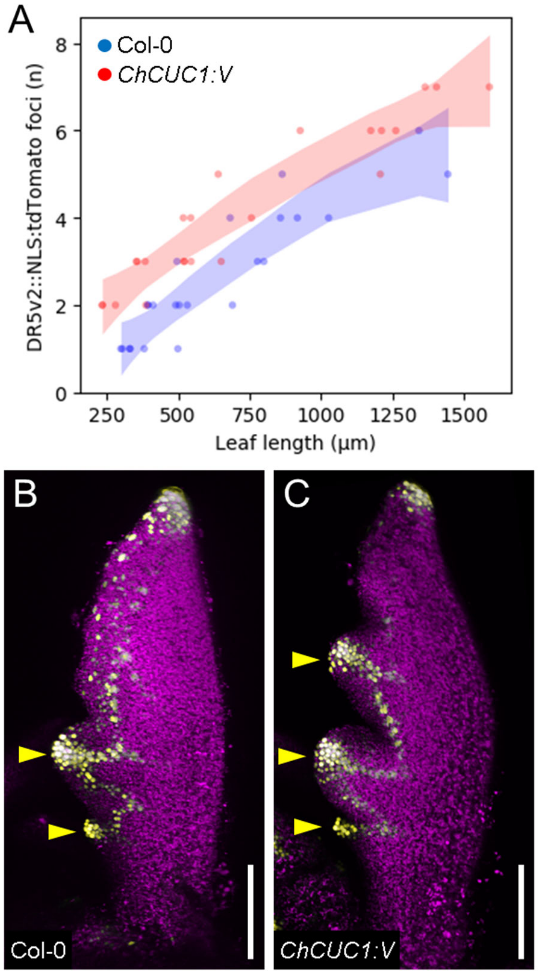

Figure S5. Number of auxin activity maxima in the leaf margins of *Arabidopsis thaliana* Col-0 and *ChCUC1p::CUC1g:Venus* during development.

(A) Number of *DR5v2::NLS:tdTomato* expression foci in the margins of *A. thaliana* Col-0 and *ChCUC1p::CUC1g:Venus* leaf 6 at different developmental stages. The color shadings indicate the 95% confidence intervals obtained in a locally-weighted regression.

(B-C) Representative images of the *DR5v2::NLS:tdTomato* expression pattern in the Col-0 (B) and *ChCUC1p::ChCUC1g:Venus* (C) backgrounds at the 500-µm stage. The yellow channel corresponds to tdTomato and the magenta to chlorophyll autofluorescence. The arrowheads indicate the marginal *DR5v2* expression foci. For clarity, Venus expression is not shown in (C). Scale bars: 100 µm.

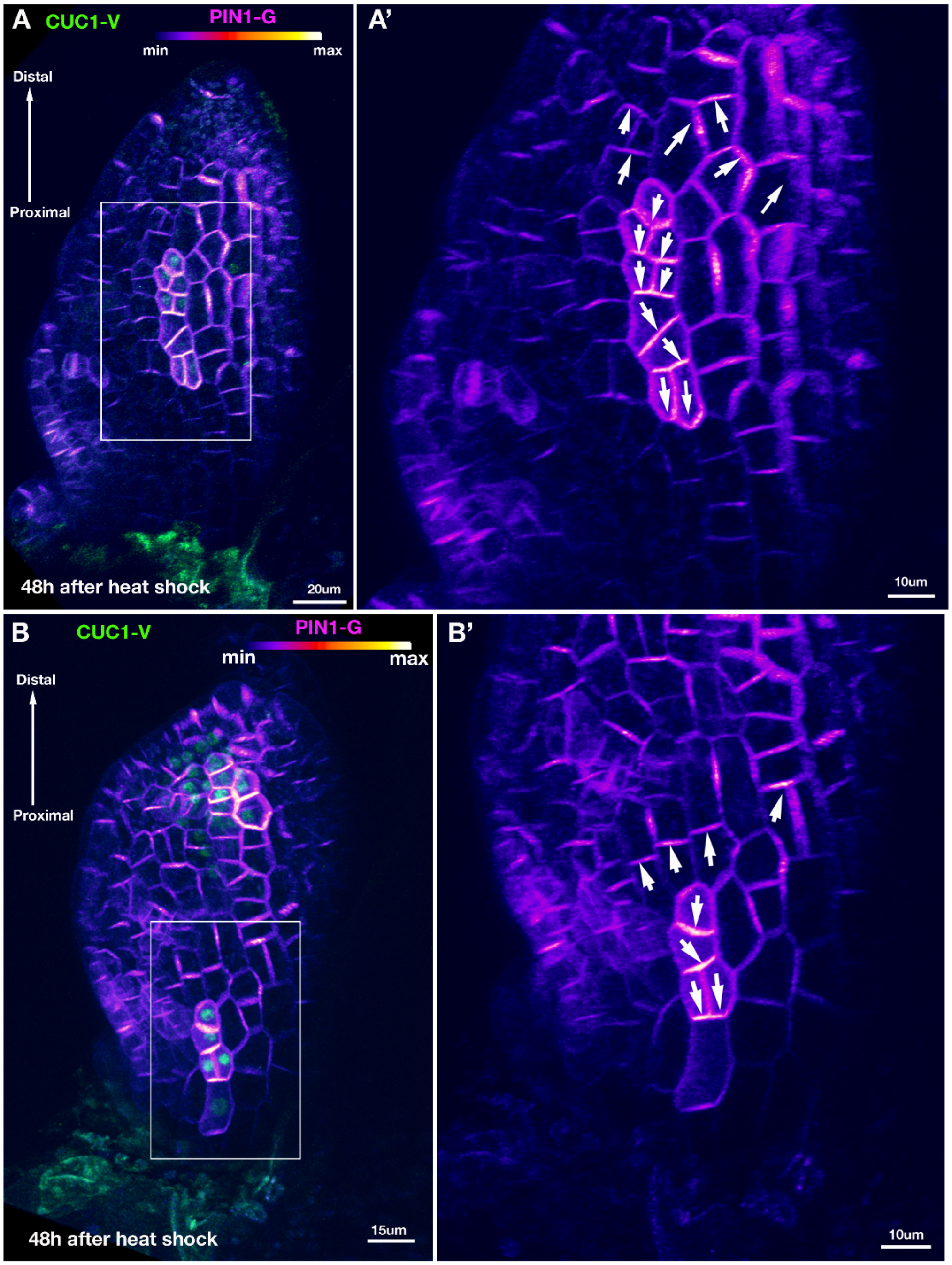

Figure S6. PIN1 polarity in the abaxial epidermis of *Arabidopsis thaliana* first leaf 48 hours after ectopic ChCUC1 clonal expression.

PIN1:GFP polarity 48 hours after heat shock induction of ectopic ChCUC1:V clones in the abaxial epidermis of leaves from the *A. thaliana* strain *HSp::dBox:Cre; 35Sp::lox--lox::ChCUC1:V*.

(A-B) Examples of epidermal ChCUC1:V clones.

(A'-B') Magnifications of insets in (A, B). The arrows indicate the polarity of PIN1:GFP. *n* = 3. The length of the scale bars is indicated on each panel.

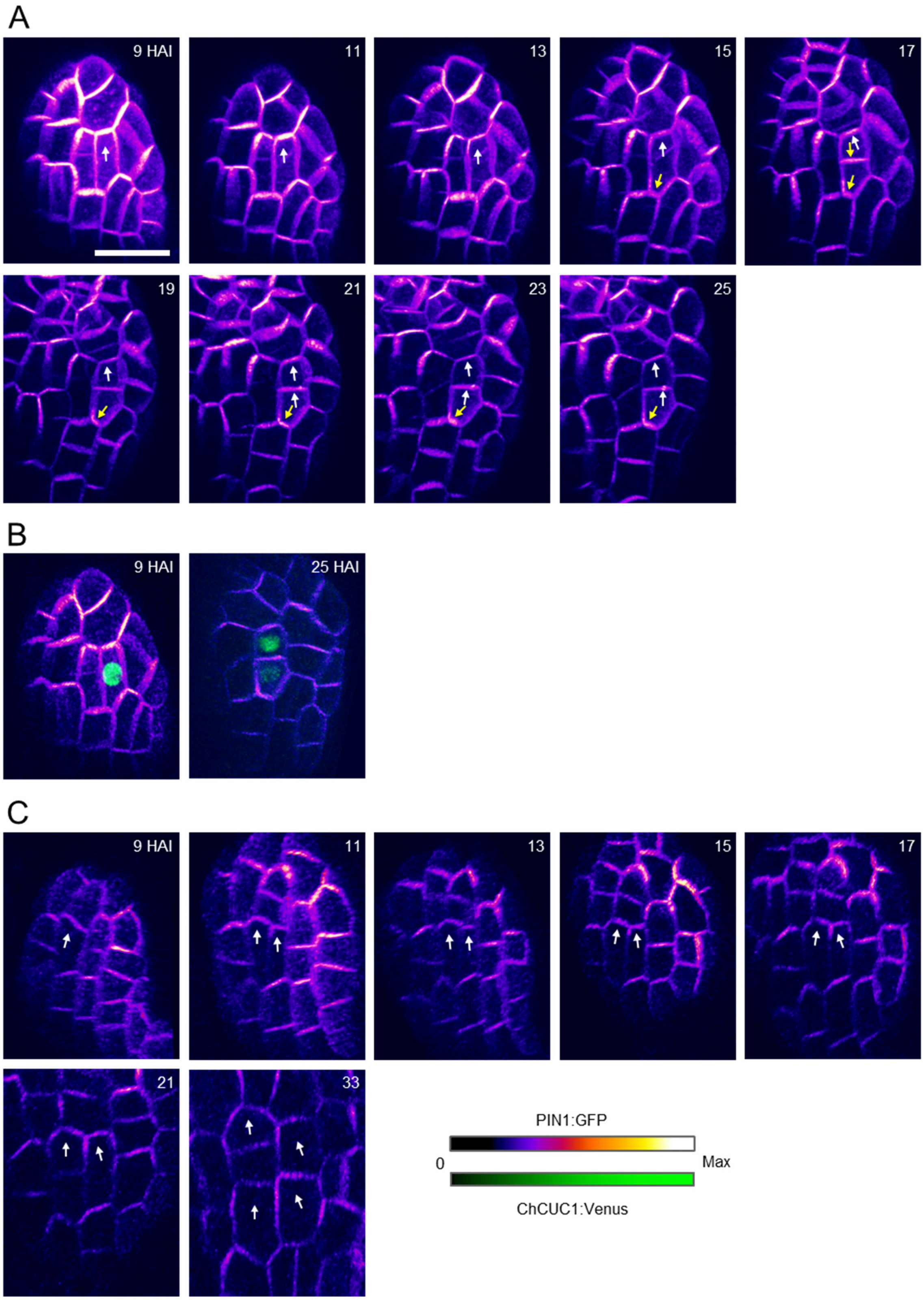

Figure S7. Polarity dynamics of PIN1:GFP after ectopic expression of ChCUC1:Venus clones.

Characterization of PIN1:GFP (PIN1p::PIN1:GFP) polarity changes after heat-shock induction of ChCUC1:V clones (*HSp::dBox:Cre*; *35Sp::lox-spacer-lox::ChCUC1:Venus*) on the abaxial epidermis of *A. thaliana* Col-0 leaf 1-2. Time-lapse imaging was initiated 9 hours after heat shock induction (HAI) to be able to identify ChCUC1:V clones for subsequent polarity tracking.
(A-B) Sample with a ChCUC1:V clone on the abaxial epidermis. (A) PIN1:GFP. Note the initial apical accumulation of PIN1:GFP in the clone (9 HAI) and the subsequent progressive repolarization (starting 15 HAI). (B) Composite of PIN1:GFP and ChCUC1:Venus channels. ChCUC1:V was only imaged in the first and last time points to minimize photobleaching.
(C) Sample with no ChCUC1:V clones, used as control. PIN1:GFP polarity in cells of equivalent position to the clone in (A, white dots) remains apical until 33 HAI.
The white arrows indicate the direction of PIN1:GFP accumulation in the ChCUC1:V clone in (A) and in cells in equivalent position in (B).
Replication information: *n* (epidermal clones) = 6 leaves; *n* (control samples) = 3 leaves.
Scale bar: 20 µm.

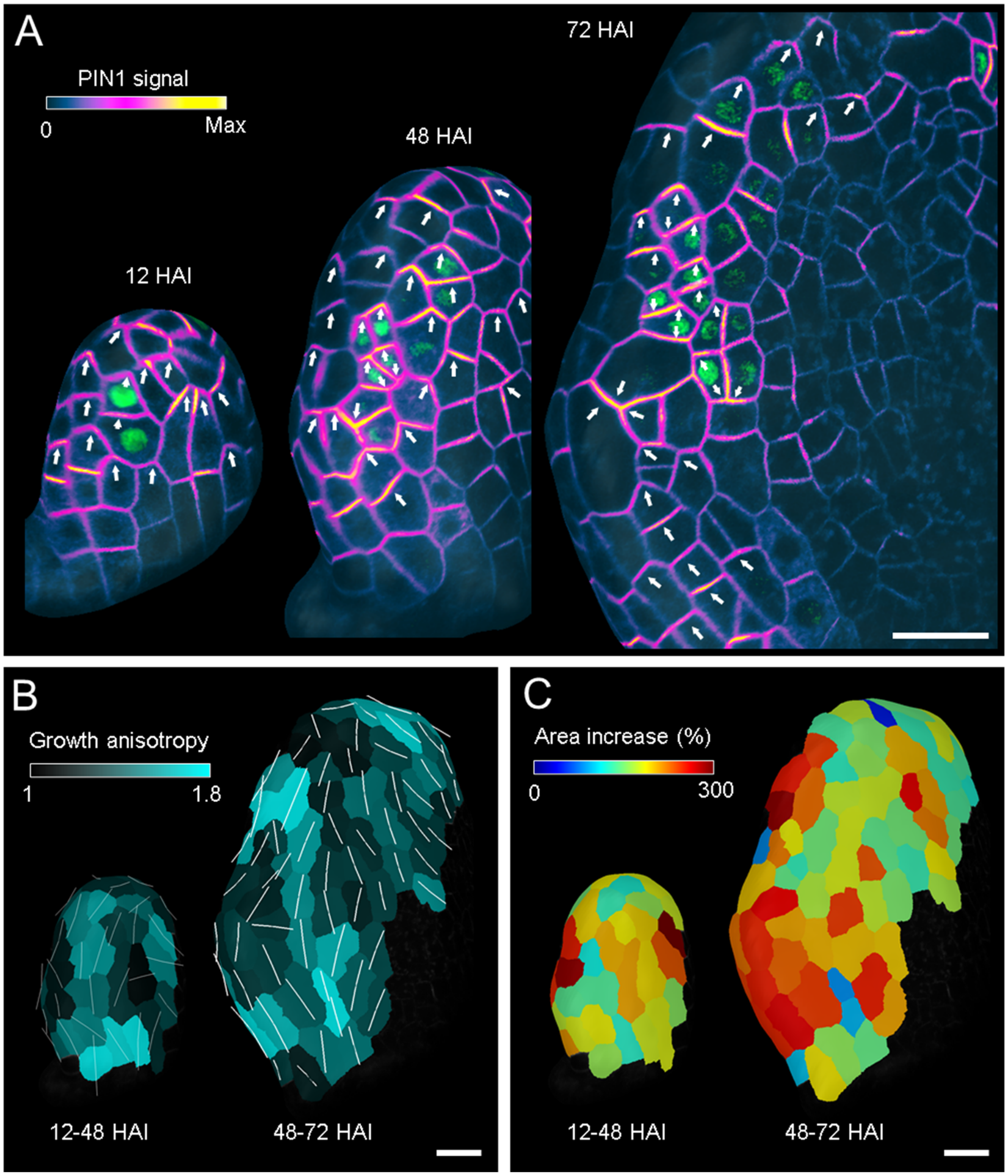

Figure S8. PIN1 polarity and growth anisotropy in the margin of *Arabidopsis thaliana* first leaf after ectopic ChCUC1 clonal expression.

(A) PIN1:GFP polarity in *A. thaliana* first leaf after induction of a sub-marginal ChCUC1:V clone. Note the polarity change from apical to bipolar in the ChCUC1:V clone and the emergence of polarity convergence point in the margin cells basally adjacent to the ChCUC1:V clone. Shown are MorphographX curved epidermal meshes with the epidermal PIN1:GFP (purple-yellow) and ChCUC1:V (green) signals projected. The arrows indicate the PIN1:GFP polarity direction. The images were obtained at the timepoints indicated. HAI: Hours after heat shock induction. Full genotype: *HSp::dBox:Cre; 35Sp::lox-spacer-lox::ChCUC1:Venus; PIN1p::PIN1:GFP*.

(B) Growth anisotropy of the sample shown in (A). The heatmap represents the ratio between the amount of growth in the maximum and the minimum principal directions of growth (PDGs)

during the time intervals indicated. The white lines indicate the direction of the maximum PDG and its magnitude. Note the emergence of polarized growth towards the margin cells basally adjacent to the ChCUC1 clone.
(C) Cell area extension of the sample shown in (A). The heatmap represents the ratio of the cell area between the timepoints indicated. Note the local inhibition of growth in the margin.
Replication: *n* = 3. Scale bars: 20 µm.

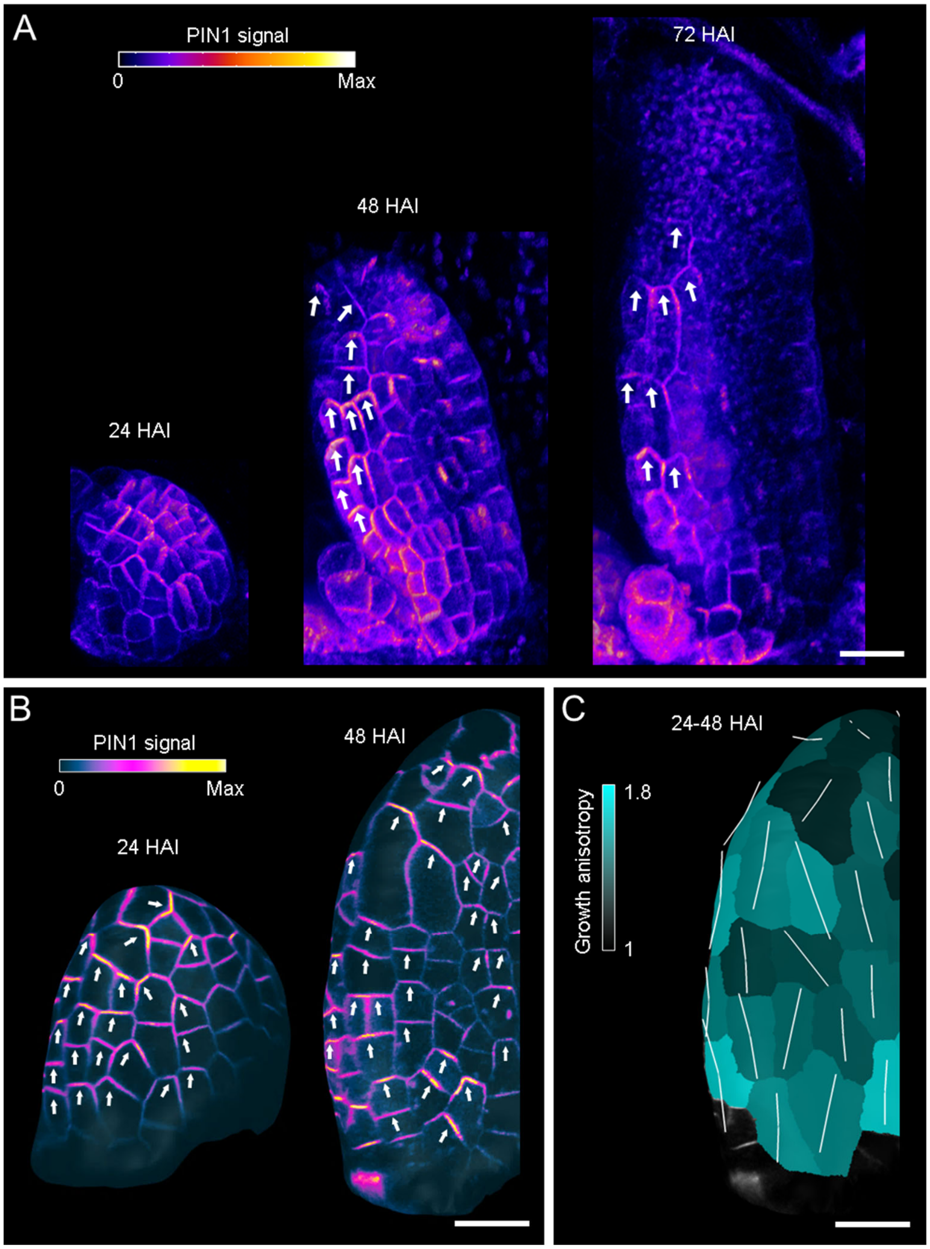

Figure S9. PIN1 polarity and growth anisotropy in the margin of heat-shocked *Arabidopsis thaliana* first leaf.

(A) PIN1:GFP polarity in leaf margin cells of *A. thaliana* Col-0 control sample at the timepoints indicated after heat shock treatment. The arrows indicate the polarity direction of PIN1:GFP. Note that PIN1:GFP polarized towards the distal tip of the primordium in most marginal cells. $n = 10$.

(B-C) PIN1:GFP polarity (B) and growth anisotropy (C) showing that both the PIN:GFP orientation and the main growth direction are aligned with the proximo-distal axis. Shown in (B) are MorphographX curved epidermal meshes with the epidermal PIN1:GFP signal projected. The heatmap in (C) represents the ratio between the amount of growth in the maximum and the minimum principal direction of growth (PDG) during the time interval indicated. The white lines indicate the direction of the maximum PDG and its magnitude.

HAI: Hours after heat shock induction. Full genotype: *PIN1p::PIN1:GFP*.
Scale bars: 20 μm.

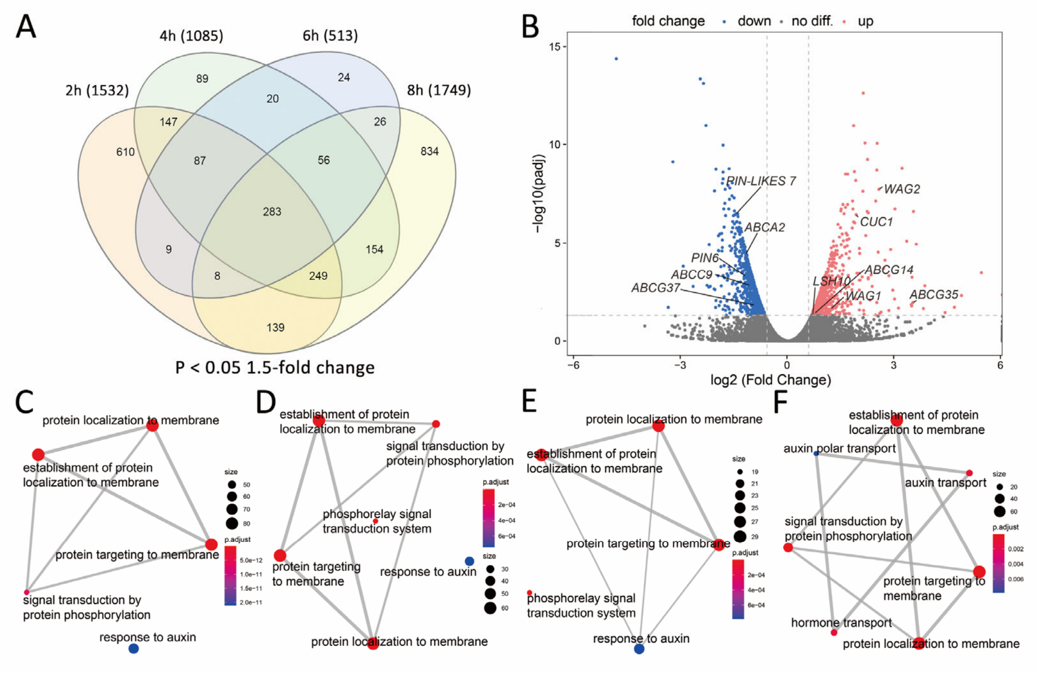

Figure S10. Analysis of time course RNA-seq expression profiling after dexamethasone induction of *RCOp::LhG4:GR; Op::ChCUC1:Venus* in *Cardamine hirsuta* leaves.

(A) Venn diagram showing the number of exclusive and common differentially expressed genes (DEGs) at different time points after *ChCUC1:V* induction. Differential expression was calculated by comparing the induced (10 μM dexamethasone) and control samples at each time point. The data was filtered by adjusted *p*-value < 0.05 and expression fold change > 1.5. The total number of DEGs at each time point is shown above the diagram.

(B) Volcano plot showing DEGs (blue dots, down-regulated DEGs and red dots, up-regulated DEGs) and non-DEGs (grey dots) 8 hours after *ChCUC1:V* induction. DEGs related to cell differentiation and auxin transport are annotated. Note that *ChWAG1* and *ChWAG2* first show up-regulation 2 and 4 hours after *ChCUC1:V* induction, respectively (Dataset S1). The *Ch* prefix has been omitted from gene names in panel B for simplicity.

(C-F) Enrichment networks for Gene Ontology (GO) biological process. The graphs show selected significantly enriched GO terms (hypergeometric test with BH correction, $\alpha \leq 0.05$) detected 2h (C), 4h (D), 6h (E), and 8h (F) after *ChCUC1:V* induction. Each GO term is represented as a circle. The size of the circle reflects the number of DEGs linked to each term, and the color indicates the adjusted *p*-value.

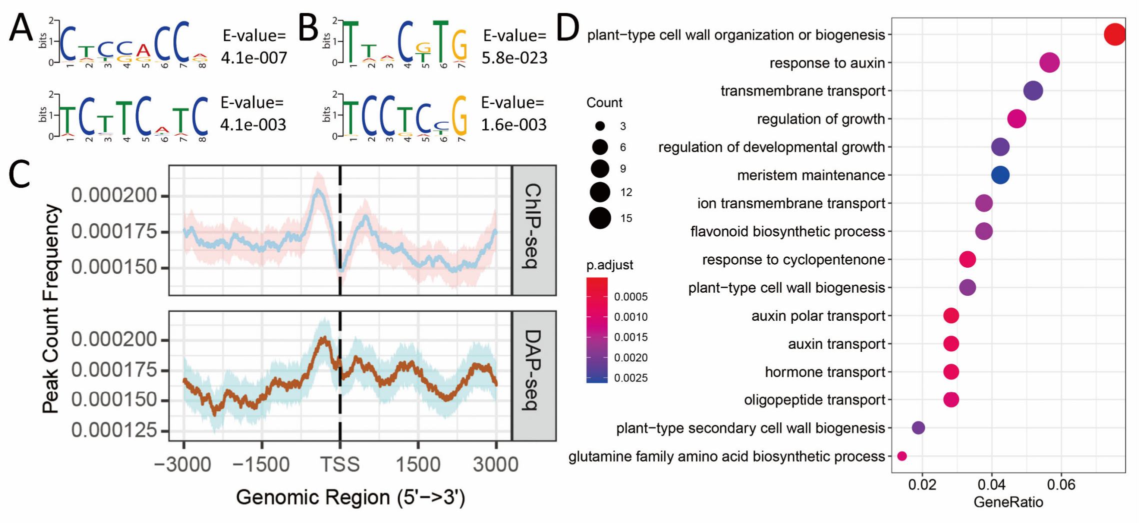

Figure S11. Analysis of ChCUC1 ChIP-seq and DAP-seq experiments.

(A-B) Binding motifs with the highest statistical significance obtained from ChIP-seq (left) and DAP-seq (right), calculated by the MEME-ChIP program (46).

(C) Distribution of ChCUC1-binding peaks according to their distance from the gene transcription start sites (TSS), as obtained for ChIP-seq and DAP-seq assays.

(D) Semantic clustering of GO categories enriched in the set of 422 high-confidence ChCUC1 binding genes (Supplementary data 3) by intersection of ChIP-seq and DAP-seq datasets. The area and color of each circle reflect the *p*-values for individual GO terms within the cluster.

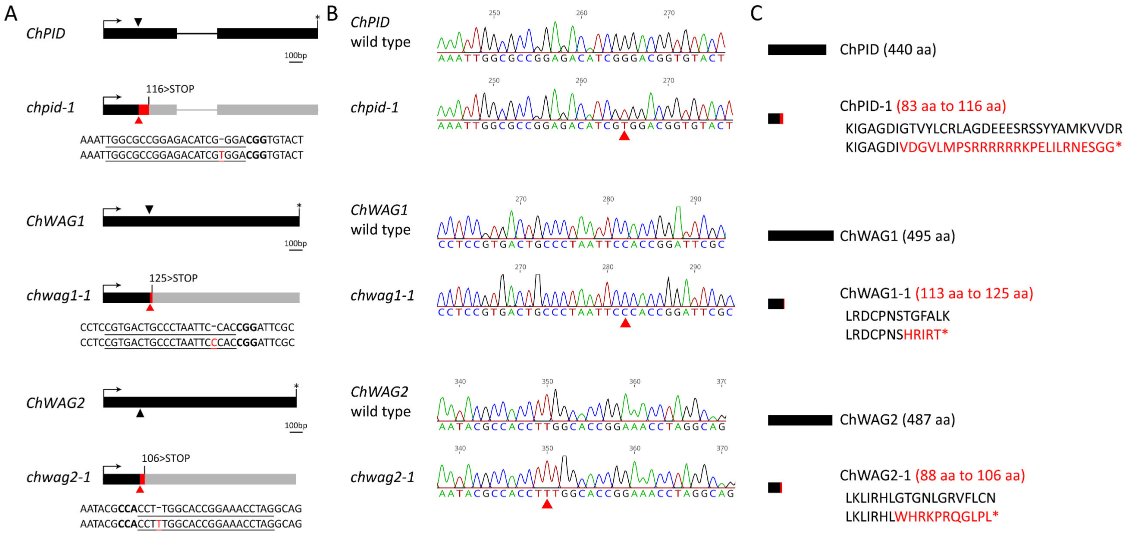

Figure S12. Characterization of CRIPSR/Cas9 alleles of *ChPID*, *ChWAG1*, and *ChWAG2*.
(A) Gene models of *ChPID*, *ChWAG1*, and *ChWAG2* indicating the positions of the CRIPSR/Cas9-induced mutations. All three independent CRISPR derived mutant alleles, *chpid-1*, *chwag1-1*, and *chwag2-1*, are caused by single nucleotide insertion followed by premature translation termination. Black boxes, exons; arrows, start codon; asterisk, termination codon; black triangles, location of CRISPR sgRNAs; red triangles, location of single nucleotide insertion; underlined DNA sequences, CRISPR sgRNAs; bold DNA sequences, PAM sites; red rectangles, missense codons caused by single nucleotide insertions; vertical black lines, position of premature stop codons; grey rectangles, untranslated region caused by premature translation termination.
(B) Representative Sanger DNA sequencing chromatograms of PCR products amplified from *ChPID*, *ChWAG1*, or *ChWAG2* loci from wild-type and corresponding mutant alleles. The red triangles indicate the inserted bases.
(C) Predicted protein products encoded by the wild-type and mutant alleles. Black rectangles, correct protein product; red rectangles, incorrect protein product due to missense translation in the mutant alleles. Red letters, mutant amino acid sequences that result from translation frameshift. Red asterisk, premature translation termination. The number of amino acids of proteins from wild-type and mutant alleles are described next to the representation. For example, ChPID (440 aa) denotes ChPID protein from wild-type which harbors 440 amino acid residues, and ChPID-1 (83 aa to 116aa) indicates that in mutant protein, aa residues 83 to 116 result from missense translation, followed by premature translation termination.

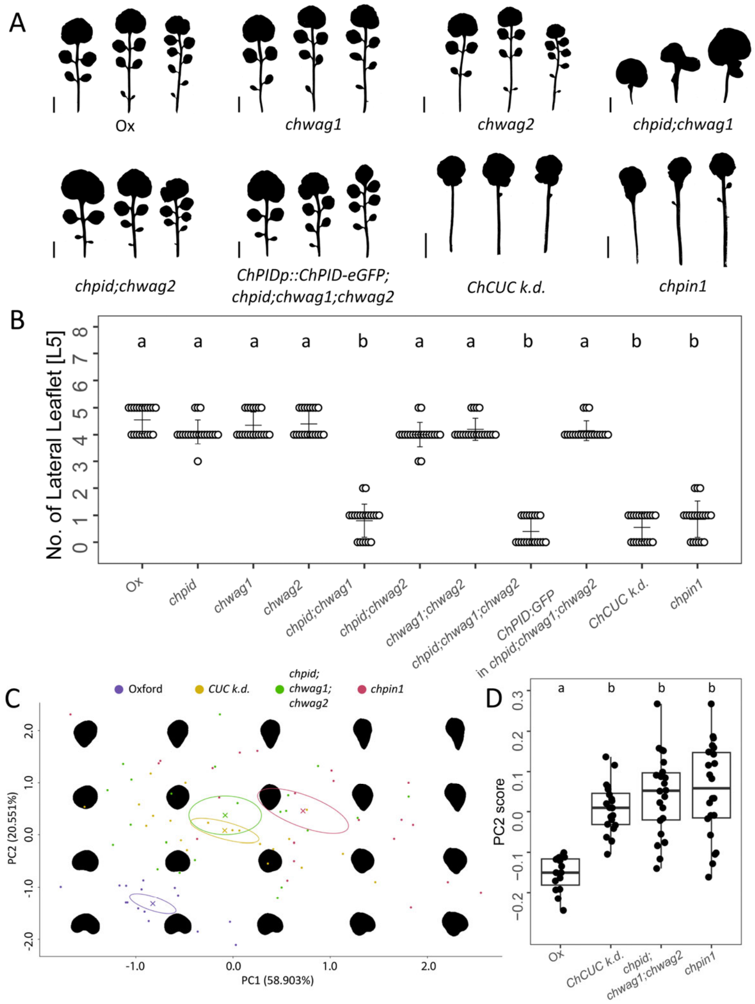

Figure S13. Characterization of the roles of *ChPID*, *ChWAG1* and *ChWAG2* in *Cardamine hirsuta* leaf development.

(A) Silhouettes showing the morphology of *C. hirsuta* rosette leaves 5, 6, and 7 from *C. hirsuta* plants with the indicated genotypes. Scale bars: 1 cm.

(B) Number of lateral leaflets on rosette leaves 5 from *C. hirsuta* plants with the indicated genotypes. The dot plot depicts mean values and standard deviation (error bars). $n \geq 20$ leaves per genotype.

(C-D) PCA-based shape analysis of the terminal leaflet from leaves of the indicated *C. hirsuta* genotypes. (C) Leaf shape-space plot. (D) Extracted PC2 values. The analysis was performed with LeafInterrogator (see materials and methods). The crosses and ellipses in (C) indicate the mean value and standard deviation of each genotype, respectively. $n \geq 15$.

The *ChPID* and *ChWAG1/2* mutant alleles are generated in this work. Other alleles have been previously described: *ChCUC knock down (ChCUC k.d.)* (*27*), *chpin1 (7)*. Letters a and b in (B, D) indicate statistically significant differences using a Kruskal-Wallis with Dunn's *post hoc* test ($\alpha = 0.05$).

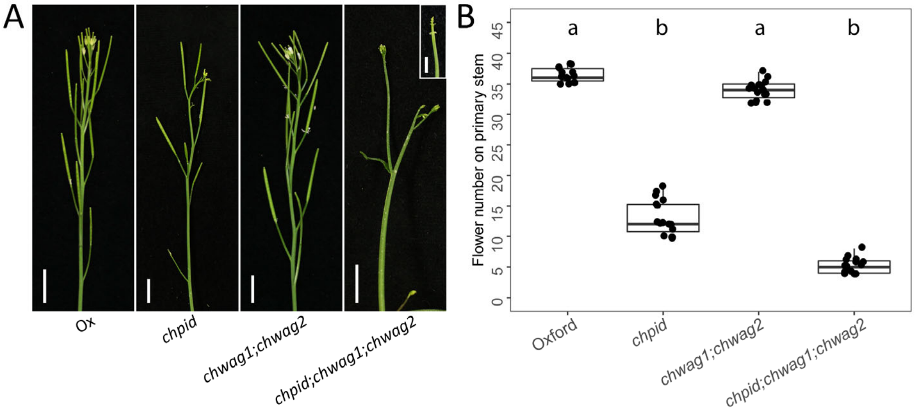

Figure S14. Characterization of the roles of *ChPID*, *ChWAG1* and *ChWAG2* in *Cardamine hirsuta* inflorescence development.

(A) Inflorescence stems of the indicated *C. hirsuta* genotypes. Note that *chpid;chwag1;chwag2* plants are infertile and show pin-like inflorescence (inset). Scale bars: 1cm or 0.2cm (inset).

(B) Number of flowers on the primary inflorescence stems of *C. hirsuta* plants with the indicated genotypes ($n \geq 10$). Letters a and b in (B) indicate statistically significant differences using a Kruskal-Wallis with Dunn's *post hoc* test ($\alpha = 0.05$).

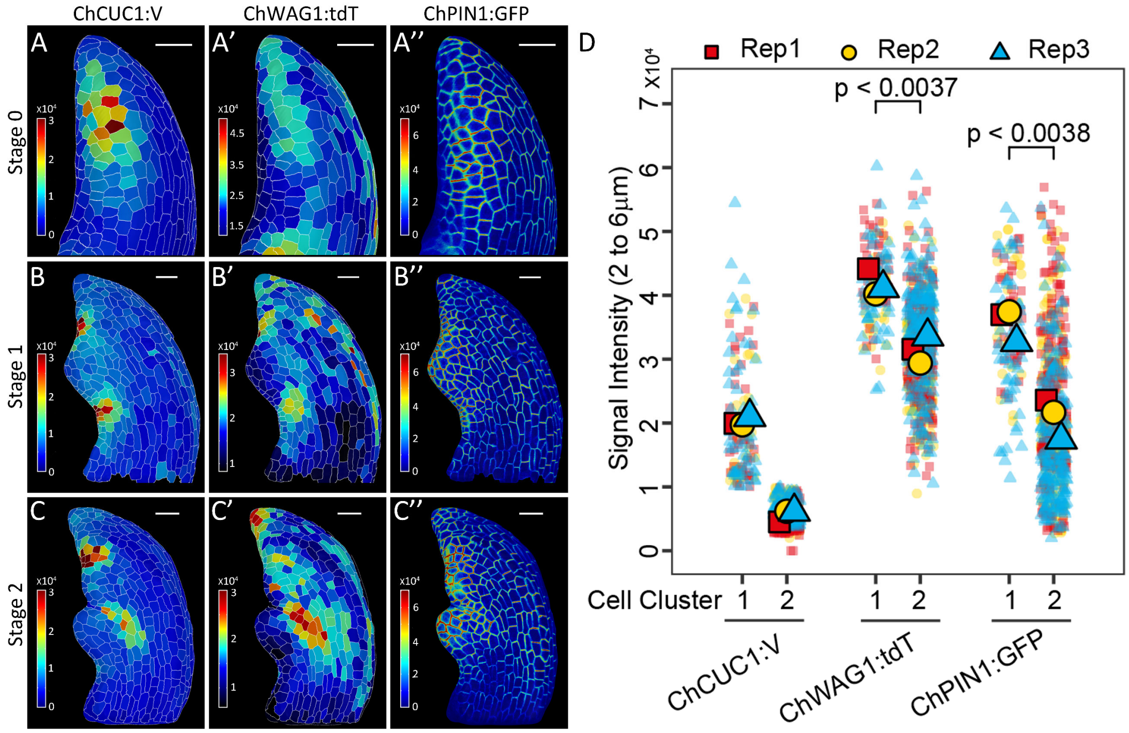

Figure S15. Correlation between expression of *ChCUC1*, *ChWAG1*, and *ChPIN1* at different *Cardamine hirsuta* leaf developmental stages.

(A-C'') Epidermal expression of *ChCUC1p::ChCUC1:Venus* (A,B,C), *ChWAG1p::ChWAG1:tdTomato* (A',B',C'), and *ChPIN1p::ChPIN1:GFP* (A'',B'',C'') in leaf 5 of *C. hirsuta*. Shown are MorphographX cell-segmented surface meshes from 3D confocal stacks with the epidermal signal from the three transgenes projected. Scale bars: 20 μm.

(D) Comparison between the expression of ChCUC1:V, ChWAG1:tdT, and ChPIN1:GFP at cell resolution after splitting the cells into two groups according to their *ChCUC1* expression level (cell clusters 1 and 2, cells in cluster 1 have high ChCUC1:V expression and cells in cluster 2 have low ChCUC1:V expression). The symbol type indicates the biological replicates. The small symbols indicate the signal intensity from each cell. The big symbols indicate the mean value of each replicate. Unpaired *t*-test. $n \geq 3$.

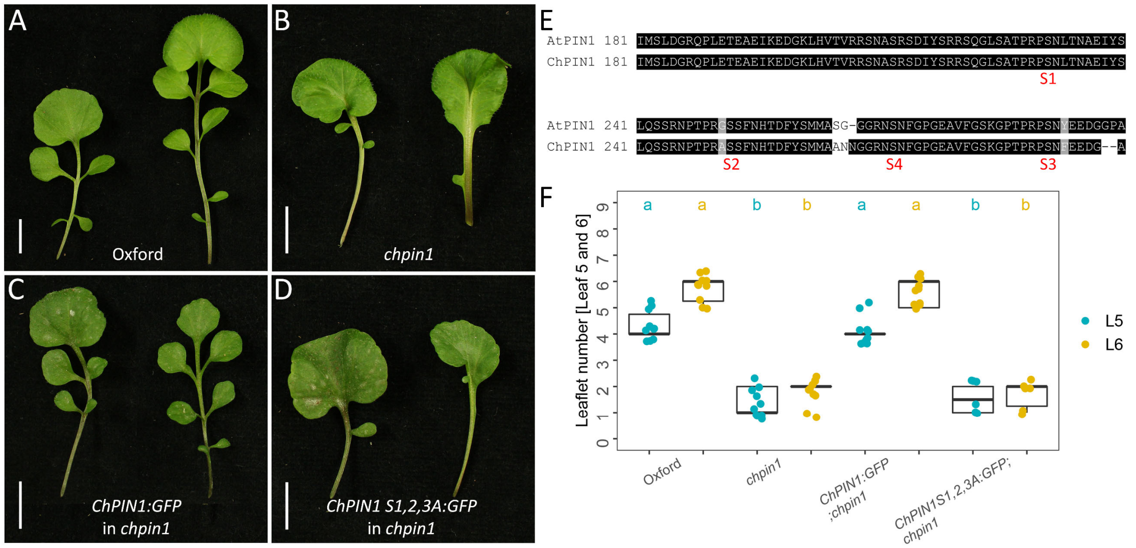

Figure S16. Phenotypes of *chpin1*, *ChPIN1:GFP* and *ChPIN1*$_{S1,2,3A}$*:GFP* in *chpin1* background.

(A-D) Rosette leaves 5 and 6 from the *C. hirsuta* genotypes indicated, wild type Ox (A), *chpin1* (B), *ChPIN1:GFP;chpin1* (C), and *ChPIN1*$_{S1,2,3A}$*:GFP;chpin1* (D) plants. Scale bars: 1 cm.

(E) Protein sequence alignment of N-terminal part of cytoplasmic hydrophilic loop of AtPIN1 and ChPIN1. The serine phosphosites targeted by PID/WAGs are conserved and indicated by the labels in red S1-S4.

(F) Number of lateral leaflets on rosette leaves 5 and 6 of *C. hirsuta* plants with the indicated genotypes ($n \geq 10$).

At least six independent T1 plants for each transgenic line were checked, and they all present similar phenotypes to those shown in (C) and (D). Letters a and b in (F) indicate statistically significant differences after a Kruskal-Wallis with Dunn's post hoc test ($\alpha = 0.05$).

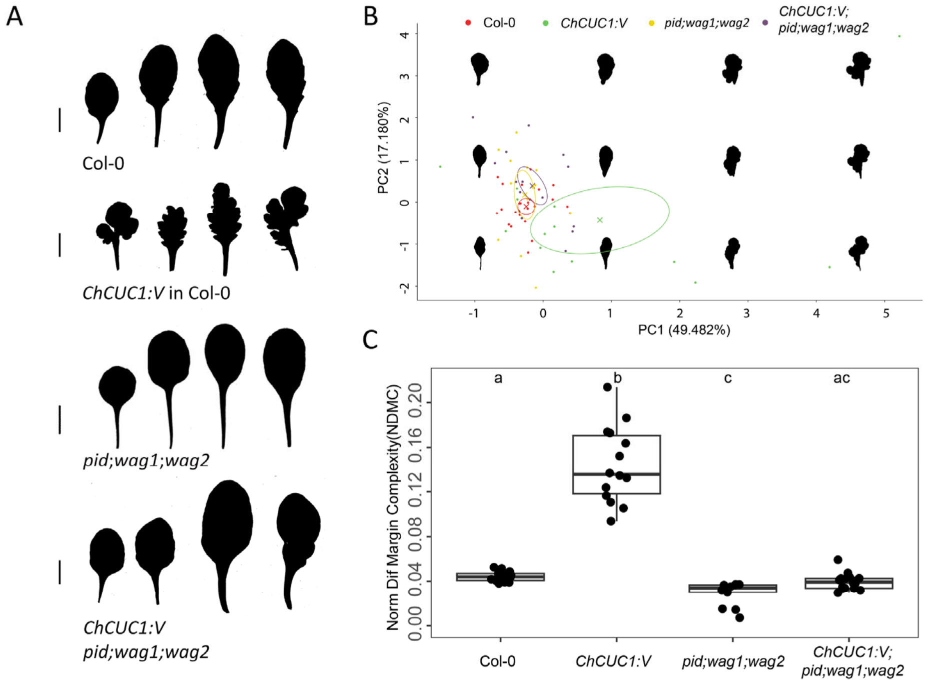

Figure S17. Simultaneous mutation of *PID, WAG1 and WAG2* suppresses the gain-of-function phenotype of *ChCUC1p::ChCUC1g:Venus* in *Arabidopsis thaliana* leaves.

(A) Silhouettes showing the morphology of four-week-old rosette leaves 5 to 8 from *A. thaliana* plants with the indicated genotypes. Scale bars: 1 cm.

(B) Shape-space plot of *A. thaliana* leaves with the indicated genotypes. Obtained through PCA-based shape analysis performed with LeafInterrogator (see materials and methods). The crosses and ellipses indicate the mean value and standard deviation of each genotype, respectively. $n \geq 13$.

(C) Margin complexity of rosette leaf 8 from *A. thaliana* plants with the indicated genotypes. Complexity was calculated as the Normalized Difference Margin Complexity [(perimeter contour-perimeter convex hull)/(perimeter contour + perimeter convex hull)]. $n \geq 13$.

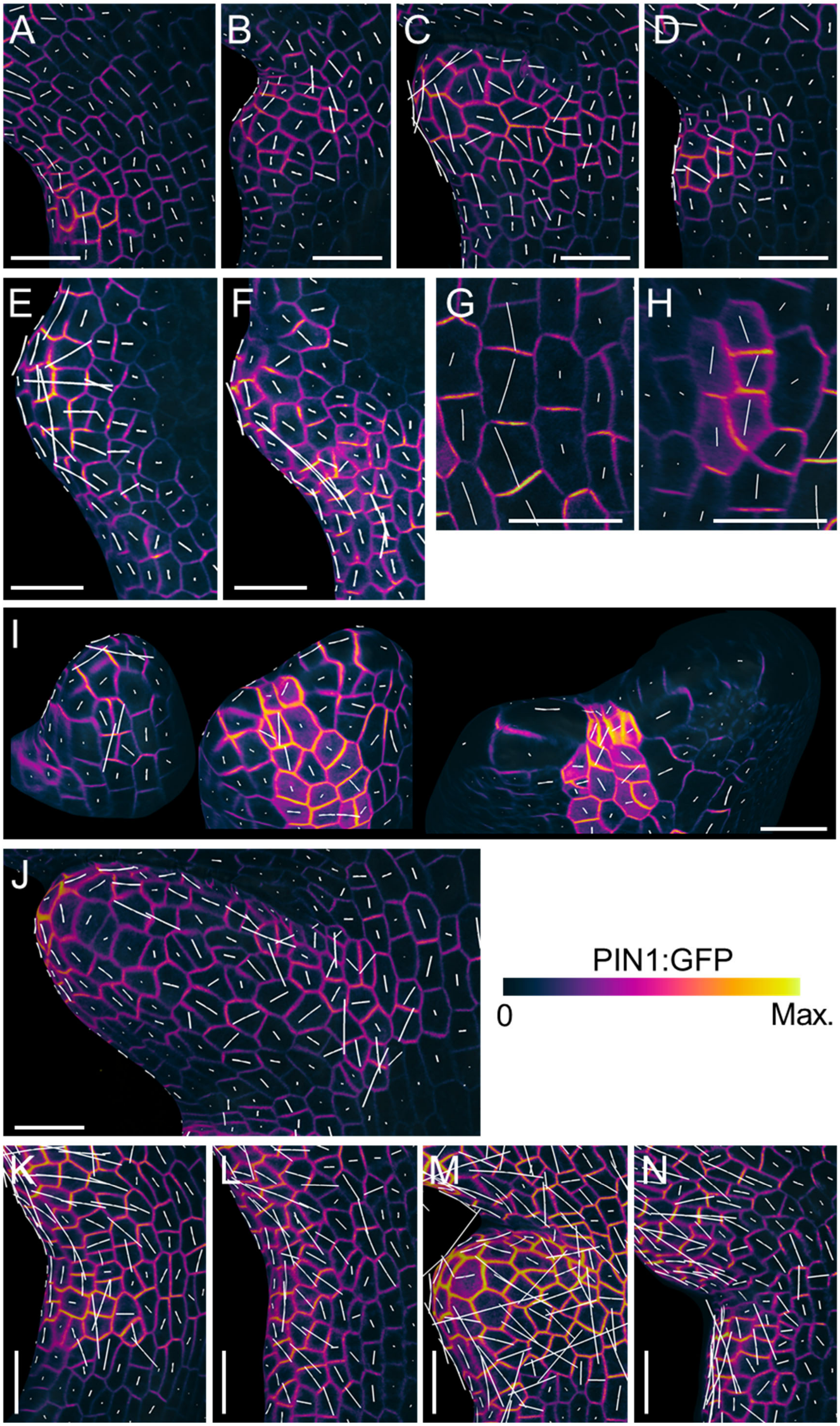
A
B
C
D
E
F
G
H
I
J
K
L
M
N
PIN1:GFP
0
Max.

Figure S18. PIN1:GFP axis orientation in selected samples as computed by the software MorphographX.

PIN1:GFP samples shown in the main text with overlaid PIN1:GFP orientation axes computed with MorphographX (white lines). These axes were used to assist during PIN1 polarity assessment (see methods). Each line corresponds to one cell. The line direction represents the cell axis with highest signal. The line length is the degree of PIN1:GFP anisotropy, or difference between the axis with highest signal and the perpendicular axis.

(A-D) Samples shown in figure 1J-M'.

(E-F) Samples shown in figure 2A.

(G-H) Samples shown in figures 2D and E.

(I) Samples shown in figure 2G.

(J) Sample shown in figure 4E.

(K-N) Samples shown in figures 4G-J.

Scale bars: 20 μm.

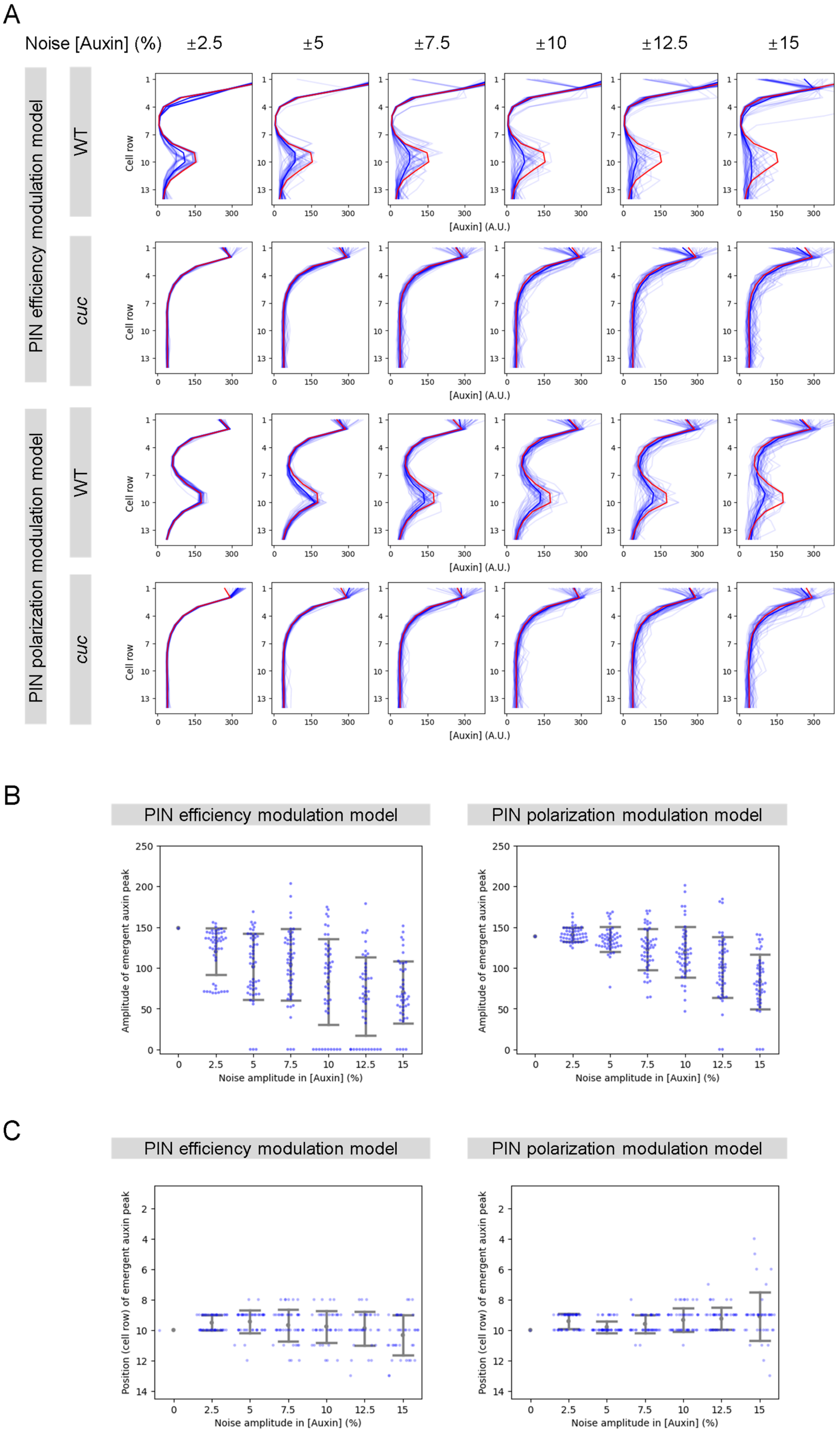
A
Noise [Auxin] (%)
±2.5
±5
±7.5
±10
±12.5
±15
PIN efficiency modulation model
PIN polarization modulation model
WT
cuc
Cell row
[Auxin] (A.U.)
B
PIN efficiency modulation model
PIN polarization modulation model
Amplitude of emergent auxin peak
Noise amplitude in [Auxin] (%)
C
PIN efficiency modulation model
PIN polarization modulation model
Position (cell row) of emergent auxin peak
Noise amplitude in [Auxin] (%)

Figure S19. Robustness analysis of the CUC-PIN-auxin computational models with different amplitudes of auxin noise.

(A) Auxin profiles along the central cell column of the simulations shown in Figure 4 with auxin noise of increasing amplitude. Thick red line: simulation without noise; thin blue lines: replicates with noise; thick blue line: average of the noise replicates. n = 50 replicates.

(B-C) Characterization of the emergent auxin peak from the simulations shown in (A). (B) Amplitude of the emergent auxin peak. Calculated as the difference between the height of the emergent auxin peak and the minimum value of the auxin profile. (C) Proximodistal position of the emergent auxin peak. n = 50 replicates. The line plot represents the mean (grey dot) ± standard deviation (whiskers).

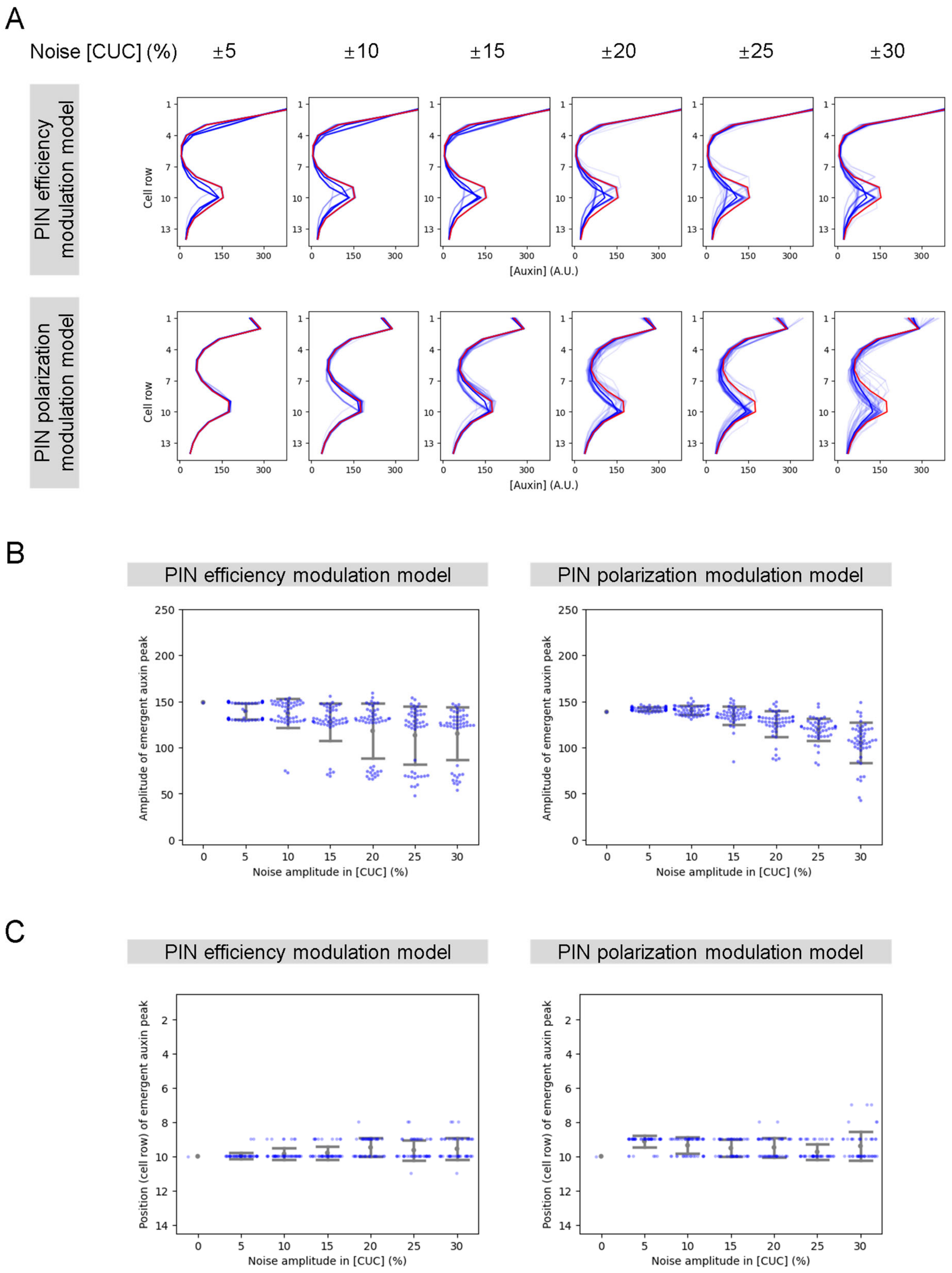

Figure S20. Robustness analysis of the CUC-PIN-auxin computational models with different amplitudes of CUC noise.

(A) Auxin profiles along the central cell column of the simulations shown in Figure 4 with auxin noise of increasing amplitude. Thick red line: simulation without noise; thin blue lines: replicates with noise; thick blue line: average of the noise replicates. n = 50 replicates.

(B-C) Characterization of the emergent auxin peak from the simulations shown in (A). (B) Amplitude of the emergent auxin peak. Calculated as the difference between the height of the emergent auxin peak and the minimum value of the auxin profile. (C) Proximodistal position of the emergent auxin peak. n = 50 replicates. The line plot represents the mean (grey dot) ± standard deviation (whiskers).

Supplementary tables

Table S1. Origin of mutant and transgenic plant strains used in this work

Plant strain	Source
Arabidopsis thaliana	
CUC1p::CUC1g:Venus* (*CUC1:V*)	This work
ChCUC1p::ChCUC1g:Venus (*ChCUC1:V*)	This work
ChCUC2p::ChCUC2g:Venus (*ChCUC2:V*)	This work
PIN1p::PIN1:GFP	(4)
cuc2-1 (isolated in *Landsberg erecta*, backcrossed to Col-0 for 5 times)	(47) (Patrick Laufs)
ChCUC1p::LhG4:GR; Op::ChCUC1:tdTomato	This work
PIN1p::PIN1:GFP; HSp::dBox:Cre; 35Sp::lox-spacer-lox::ChCUC1:Venus	This work
DR5v2::NLS:tdTomato	(14)
UBQ10p::acyl:tdTomato	(48) (Elliot Meyerowitz)
wag1 (SALK-002056); *wag2* (SALK-070240)	(49) (Claus Schwechheimer)
pid-14 (SALK-049736)	(49) (Claus Schwechheimer)
pid-14; wag1; wag2	(49) (Claus Schwechheimer)
Cardamine hirsuta	
ChCUC1p::ChCUC1g:Venus (*ChCUC1:V*)	This work
RCOp::LhG4:GR; Op::ChCUC1:Venus	This work
35S::miR164B; 35S::CUC3-RNAi (*CUC k.d.*)	(50) (Patrick Laufs)
chpid-1	This work
chwag1-1	This work
chwag2-1	This work
ChPIDp::ChPID:eGFP (*ChPID:GFP*)	This work
ChWAG1p::ChWAG1g:tdTomato (*ChWAG1:tdT*)	This work
ChCUC1p::ChWAG1:tdT	This work
chpin1	(10)
ChPIN1p::ChPIN1g:eGFP (*ChPIN1:GFP*)	This work
ChPIN1p::ChPIN1-S123A:eGFP	This work

**g* indicates genomic sequence (coding sequence with introns).

Table S2. Primers for genotyping

Name	Sequence (5’→3’)	Use
ChWAG1-CRISPR-F	GACGGTTATTATAATCTCGACACCG	for genotyping *ChWAG1* CRISPR mutant. PCR product 860bp.
ChWAG1-CRISPR-R	TCTCGCTCGTACTTAACCTCC	for genotyping *ChWAG1* CRISPR mutant. PCR product 860bp.
ChWAG2-CRISPR-F	TCCGCTAAGGTCAAAACCATCA	for genotyping *ChWAG2* CRISPR mutant. PCR product 873bp.
ChWAG2-CRISPR-R	ACCGTCTTCACGGAGCAAAA	for genotyping *ChWAG2* CRISPR mutant. PCR product 873bp.
ChPID-CRISPR-F	TCACCGAGACTATCCTCTGTTAAA	for genotyping *ChPID* CRISPR mutant. PCR product 854bp.
ChPID-CRISPR-R	AGAACTGAAAAAGACCGACATGA	for genotyping *ChPID* CRISPR mutant. PCR product 854bp.
chpin1-1-Pst1-F2	GGAAATAACATAAGCAACAAGACGCtG	dCAPS primer for genotyping *chpin1-1*
chpin1-1-Pst1-R2	TTGAAAGAGATGAGGGACCAGG	dCAPS primer for genotyping *chpin1-1*
LBb1.3	ATTTTGCCGATTTCGGAAC	Newly used by Salk Genotyping Project and with better results
pinoid14-LP	CAGTCGGGAAACTCAACTGTC	Left genomic primer for genotyping SALK_049736
pinoid14-RP	ATTTTGCGATGAAAGTTGTGG	Right genomic primer for genotyping SALK_049736
wag1-LP	TATATTGCGCAGGGTTTGTTC	Left genomic primer for genotyping SALK_002056
wag1-RP	TCTCGATCTCAGCTTCACCTC	Right genomic primer for genotyping SALK_002056
wag2-LP	TAAAGGAATATTCCGAACGCC	Left genomic primer for genotyping SALK_070240
wag2-RP	CCAAAACCCCCAAACATAAAC	Right genomic primer for genotyping SALK_070240

Table S3. Primers for generation of expression constructs

Name	Sequence (5'→3')	Use
AtCUC1p-SalI-F	ACGCgtcgacGGTTTAAATTTGGAAGAAAT	Amplification of the *AtCUC1* promoter region for construction of *AtCUC1p::AtCUC1g:Venus*
AtCUC1p-XhoI-R	CCGctcgagTGTCGGCACAAGAAGAAAGC	Amplification of the *AtCUC1* promoter region for construction of *AtCUC1p::AtCUC1g:Venus*
AtCUC1g-XhoI-F	CCGctcgagATGGCTGCGGCTGCTGCCGC	Amplification of the *AtCUC1* transcribed region for construction of *AtCUC1p::AtCUC1g:Venus*
AtCUC1g-NcoI-R	CATGccatggCAGTGTGTGGCCGTTTACTCTC	Amplification of the *AtCUC1* transcribed region for construction of *AtCUC1p::AtCUC1g:Venus*
ChCUC1p-BamHI-F	ggatccTAGGACCTTCACGGGTTTCA	Amplification of the *ChCUC1* genomic region for construction of *ChCUC1p::ChCUC1g:Venus*
ChCUC1-NcoI-R	CAGccatggGAGAGCAAACGGCCAGTAACTC	Amplification of the *ChCUC1* genomic region for construction of *ChCUC1p::ChCUC1g:Venus*
attB1-ChCUC1p-F	ggggacaagtttgtacaaaaaagcaggcttAGGACCTTCACGGGTTT	Amplify *ChCUC1* promoter to clone in pBIN-LR-LhGR2
attB2-ChCUC1p-R	ggggaccactttgtacaagaaagctgggttGTAGAGGCAACACAGGAGA	Amplify *ChCUC1* promoter to clone in pBIN-LR-LhGR2
attB1-ChRCOp-F	ggggacaagtttgtacaaaaaagcaggctttTCTGAGATAGAGAAGAGAGTC	Amplification of the *ChRCO* promoter region for construction of *RCOp::LhGR/ChCUC1:Venus*
attB2-ChRCOp-R	ggggaccactttgtacaagaaagctgggttTTCCTTTCCTTAATAAGAAGAC	Amplification of the *ChRCO* promoter region for construction of *RCOp::LhGR/ChCUC1:Venus*
ChWAG1c-XmaI-F	TCCCcccgggATGGAAGACGACGGTTATTA	Amplification of the *ChWAG1* transcribed region for construction of *ChWAG1p::ChWAG1:tdT*
ChWAG1c-BamHI-R	CGCggatccAACGCGTTTGCGAGTAGCGTT	Amplification of the *ChWAG1* transcribed region for construction of *ChWAG1p::ChWAG1:tdT*
ChWAG1p-EcoRI-F	CCGgaattcCGTTTGTTATGATTAGAATCGAACCAC	Amplification of the *ChWAG1* promoter unit for construction of *ChWAG1p::ChWAG1:tdT*
ChWAG1p-XmaI-R	TCCCcccgggTTTTTTCTGATGAAGTTTGTTTTTGGT	Amplification of the *ChWAG1* promoter unit for construction of *ChWAG1p::ChWAG1:tdT*
ChWAG2c-XmaI-F	TCCCcccgggATGGAACTTCAAGATTTCTA	Amplification of the *ChWAG2* transcribed region for construction of *ChWAG2p::ChWAG2:tdT*
ChWAG2c-BamHI-R	CGCggatccAACGCGTTTACGACTCGCGTA	Amplification of the *ChWAG2* transcribed region for construction of *ChWAG2p::ChWAG2:tdT*
ChWAG2p-EcoRI-F	CCGgaattcAAAGTGAAAGAGAAAGCTGAAT	Amplification of the *ChWAG2* promoter unit for construction of *ChWAG2p::ChWAG2:tdT*
ChWAG2p-XmaI-R	TCCCcccgggTTTTTGTGTTTCTTGATTCACTT	Amplification of the *ChWAG2* promoter unit for construction of *ChWAG2p::ChWAG2:tdT*
ChPIDp-EcoRI-F	CCGgaattcTATAAAATATTAATATTTGTCACACTTTCT	Amplification of the *ChPID* promoter unit for construction of *ChPIDp::ChPID:eGFP*
ChPIDp-XmaI-R	TCCCcccgggCTCCGGGAAATCGAAGTTAAATCAA	Amplification of the *ChPID* promoter unit for construction of *ChPIDp::ChPID:eGFP*
ChPIN1p-XhoI-F	CCGctcgagTCCAATTTTACCCCTATCCC	Amplification of the *ChPIN1* promoter region for construction of *ChPIN1p::ChPIN1:eGFP*
ChPIN1p-KpnI-R	CGGggtaccTTTTTTTCGCCGGAGAGTGGAG	Amplification of the *ChPIN1* promoter region for construction of *ChPIN1p::ChPIN1:eGFP*

ChCUC1p-PstI-F	AAAActgcagTAGGACCTTCACGGGTTTCA	Amplification of the *ChCUC1* promoter region for construction of *ChCUC1p::ChWAG1*
ChCUC1p-XmaI-R	TCCCcccgggTGTAGAGGCAACACAGGAGA	Amplification of the *ChCUC1* promoter region for construction of *ChCUC1p::ChWAG1*

Table S4. Primers for quantitative RT-PCR

Name	Sequence (5’→3’)	Use
TIP41_qF	GATGGTGTGCTTATGAGATTGAGAG	Housekeeping gene
TIP41_qR	TCAACTGGATACCCTTTCGCA	Housekeeping gene
ChUBQ10_qF	TGGTACTTTTGTGTGTTTTGAGGC	Housekeeping gene
ChUBQ10_qR	AAAGAGAGATAAGGACGCAAACATAGT	Housekeeping gene
ChWAG1-qF	TAGATTGGTGGGCGTTTGGG	*ChWAG1* forward for *C. hirsuta*. Product length 141bp.
ChWAG1-qR	CCTCTTCGTCTCGCTCCAAC	*ChWAG1* reverse for *C. hirsuta*. Product length 141bp.
ChWAG2-qF	AAGTGGCCGTTGATTAGGCA	*ChWAG2* forward for *C. hirsuta*. Product length 138bp.
ChWAG2-qR	AGCCCTCCACAAAAACCACT	*ChWAG2* reverse for *C. hirsuta*. Product length 138bp.
ChCUC1-qF	GCCACCTGGGTTTAGGTTTC	*ChCUC1* forward for *C. hirsuta*
ChCUC1-qR	GCACAGGAGAAATTGGAGTCA	*ChCUC1* reverse for *C. hirsuta*
ChLAS-qF	ACGACGACGGAGATATGCTTG	*ChLAS* forward for *C. hirsuta*
ChLAS-qR	ATGATCCACAGCCTCGGAGA	*ChLAS* reverse for *C. hirsuta*

Table S5. Parameter values used in the simulations shown in this work

Description	Symbol	Model	
		PIN efficiency modulation model	PIN polarization modulation model
Auxin diffusion coefficient	k_D	.3	.4
Auxin production rate	k_{AS}	.8	.8
Auxin degradation rate	k_{AT}	.02	.02
Transport efficiency of unphosphorylated PIN	T_{basal}	.007	.011
Transport efficiency of phosphorylated PIN	T_{pho}	.07	N/A
Effect of the middle domain identity on CUC production	k_{MDC}	.08[a]	.15[a]
Effect of the proximal identity on CUC degradation	k_{PC}	.1	.1
Sensitivity of CUC to degradation by auxin	k_{AC}	.003	.002
CUC turnover rate	k_{CT}	.1	.1
PIN synthesis rate	k_{PS}	.1	.1
PIN turnover rate	k_{PT}	.025	.025
Effect of auxin on PIN synthesis	k_{AP}	.0001	.0001
Effect of CUC on PIN synthesis	k_{CP}	.01	.02
Effect of CUC on PIN phosphorylation (half saturation)	$k_{0.5}$	5	5
Effect of CUC on PIN phosphorylation (Hill coefficient)	H	1	2
Maximum sensitivity of PIN to differences in auxin concentration in neighbors	b_{max}	1.2	1.2
Sensitivity of PIN to differences in auxin flux in cell edges	c	N/A	2
Effect of auxin efflux on F allocation to cell edges	α	N/A	1
F deallocation rate from cell edges	γ	N/A	.1
Middle domain identity for each tissue column	MD	[0,4,6,8,9,8,6,4,0]	[0,4,6,9,9,9,6,4,0]
Proximal identity for every tissue row	$PROX$	[0,0,0,0,0,0,1, 3,5,8,9,9,9,9,9]	[0,0,0,0,0,0,0, 0,1,3,5,8,9,9,9]
Auxin perfect source	$A_{(6,2)}$	300	300
Auxin noise limit (%)	ε_A	0 - 15	0 - 15
CUC noise limit (%)	ε_C	0 - 20	0 - 20
Integration time step	dt	0.1 - 0.001[b]	0.1 - 0.001[b]

N/A. Not applicable.

[a] The *cuc* mutant was simulated by setting k_{MDC} to 0.

[b] In simulations where auxin or CUC noise was applied, dt = 0.1.

Supplementary datasets and movies

Dataset S1. Time-course transcriptome analysis of *Cardamine hirsuta RCOp::LhG4:GR; 6xOp::ChCUC1:Venus*.

This data consists of a .xlsx file with multiple tabs. Differentially expressed genes (fold change > 1.5 and adjusted *p*-value < 0.05) 2, 4, 6 and 8 hours after induction of *RCOp::LhG4:GR; 6xOp::ChCUC1:Venus* with dexamethasone, and list of ChCUC1 responsive leaf genes (genes showing differential expression at more than one time).

Dataset S2. Enrichment peaks and their associated genes identified by ChCUC1 ChIP-seq and DAP-seq.

This data consists of a .xlsx file with multiple tabs. The following gene structure categories were defined: promoter (-3kb to -1kb), promoter-TSS (-1kb to +100bp), CDS exon, intron, TTS (+100 bp to +1kb), intergenic.

Dataset S3. High-confidence ChCUC1 targets.

This data consists of a .xlsx file with multiple tabs. ChCUC1 high confidence binding genes, defined as the intersection between those detected by ChIP-seq and DAP-seq. ChCUC1 high confidence targets, defined as the intersection between ChCUC1 high confidence binding genes and the genes differentially expressed after dexamethasone induction of *Cardamine hirsuta RCOp::LhG4:GR; 6xOp::ChCUC1:Venus*.

Movie S1. Simulation of leaf margin patterning using the PIN polarization modulation model.

This movie corresponds to a simulation using the parameter values described in Table S5 (PIN polarization modulation model). Auxin, red; PIN, green; CUC, magenta.

Movie S2. Simulation of leaf margin patterning using the PIN efficiency modulation model.

This movie corresponds to a simulation using the parameter values described in Table S5 (PIN efficiency modulation model). Auxin, red; PIN, green; CUC, magenta.

References

1. Y. Eshed, S. F. Baum, J. L. Bowman, Distinct mechanisms promote polarity establishment in carpels of Arabidopsis. *Cell* **99**, 199-209 (1999).
2. M. I. Rast-Somssich *et al.*, Alternate wiring of a *KNOXI* genetic network underlies differences in leaf development of *A. thaliana* and *C. hirsuta*. *Genes Dev.* **29**, 2391-2404 (2015).
3. J. Craft *et al.*, New pOp/LhG4 vectors for stringent glucocorticoid-dependent transgene expression in Arabidopsis. *Plant J.* **41**, 899-918 (2005).
4. E. Benková *et al.*, Local, efflux-dependent auxin gradients as a common module for plant organ formation. *Cell* **115**, 591-602 (2003).
5. R. Siligato *et al.*, MultiSite gateway-compatible cell type-specific gene-inducible system for plants *Plant Physiol.* **170**, 627-641 (2015).
6. M. Karimi, B. De Meyer, P. Hilson, Modular cloning in plant cells. *Trends Plant Sci.* **10**, 103-105 (2005).
7. S. Sprunck *et al.*, Egg cell-secreted EC1 triggers sperm cell activation during double fertilization. *Science* **338**, 1093-1097 (2012).
8. F. Fauser, S. Schiml, H. Puchta, Both CRISPR/Cas-based nucleases and nickases can be used efficiently for genome engineering in Arabidopsis thaliana. *Plant J.* **79**, 348-359 (2014).
9. M. G. Heisler *et al.*, Patterns of auxin transport and gene expression during primordium development revealed by live imaging of the Arabidopsis inflorescence meristem. *Curr. Biol.* **15**, 1899-1911 (2005).
10. M. Barkoulas, A. Hay, E. Kougioumoutzi, M. Tsiantis, A developmental framework for dissected leaf formation in the Arabidopsis relative *Cardamine hirsuta*. *Nat. Genet.* **40**, 1136-1141 (2008).
11. F. Huang *et al.*, Phosphorylation of conserved PIN motifs directs *Arabidopsis* PIN1 polarity and auxin transport. *Plant Cell* **22**, 1129-1142 (2010).
12. A. S. Hay *et al.*, *Cardamine hirsuta*: a versatile genetic system for comparative studies. *Plant J.* **78**, 1-15 (2014).
13. T. L. Shimada, T. Shimada, I. Hara-Nishimura, A rapid and non-destructive screenable marker, FAST, for identifying transformed seeds of *Arabidopsis thaliana*. *Plant J.* **61**, 519-528 (2010).
14. Z. Zhang *et al.*, A WOX/auxin biosynthesis module controls growth to shape leaf form. *Curr. Biol.* **30**, 4857-4868.e4856 (2020).
15. A. Leigh, S. Sevanto, J. D. Close, A. B. Nicotra, The influence of leaf size and shape on leaf thermal dynamics: does theory hold up under natural conditions? *Plant, Cell Environ.* **40**, 237-248 (2017).
16. F. P. Kuhl, C. R. Giardina, Elliptic Fourier features of a closed contour. *Comput. graph. image process.* **18**, 236-258 (1982).
17. N. Bhatia *et al.*, Auxin acts through MONOPTEROS to regulate plant cell polarity and pattern phyllotaxis. *Curr. Biol.* **26**, 3202-3208 (2016).
18. P. Barbier de Reuille *et al.*, MorphoGraphX: A platform for quantifying morphogenesis in 4D. *eLife* **4**, e05864 (2015).
19. S. Strauss *et al.*, Using positional information to provide context for biological image analysis with MorphoGraphX 2.0. *eLife* **11**, e72601 (2022).
20. T. D. Schmittgen, K. J. Livak, Analyzing real-time PCR data by the comparative CT method. *Nat. Protoc.* **3**, 1101-1108 (2008).
21. O. S. Lau, D. C. Bergmann, MOBE-ChIP: a large-scale chromatin immunoprecipitation assay for cell type-specific studies. *Plant J.* **84**, 443-450 (2015).
22. A. Bartlett *et al.*, Mapping genome-wide transcription-factor binding sites using DAP-seq. *Nat. Protoc.* **12**, 1659-1672 (2017).
23. S. Anders, W. Huber, Differential expression analysis for sequence count data. *Genome Biol* **11**, R106 (2010).

24. R. Mott *et al.*, The architecture of parent-of-origin effects in mice. *Cell* **156**, 332-342 (2014).
25. G. Yu, L.-G. Wang, Y. Han, Q.-Y. He, clusterProfiler: an R package for comparing biological themes among gene clusters. *OMICS: J. Integrative Biol.* **16**, 284-287 (2012).
26. G. Yu, L.-G. Wang, Q.-Y. He, ChIPseeker: an R/Bioconductor package for ChIP peak annotation, comparison and visualization. *Bioinformatics* **31**, 2382-2383 (2015).
27. R. S. Smith *et al.*, A plausible model of phyllotaxis. *Proc. Natl. Acad. Sci. U. S. A.* **103**, 1301-1306 (2006).
28. G. D. Bilsborough *et al.*, Model for the regulation of *Arabidopsis thaliana* leaf margin development. *Proc. Natl. Acad. Sci. U. S. A.* **108**, 3424-3429 (2011).
29. A. Runions, R. S. Smith, P. Prusinkiewicz, Computational models of auxin-driven development. *Auxin and its role in plant development*, 315-357 (2014).
30. M. Zourelidou *et al.*, Auxin efflux by PIN-FORMED proteins is activated by two different protein kinases, D6 PROTEIN KINASE and PINOID. *eLife* **3**, e02860 (2014).
31. P. Wang *et al.*, Phosphatidic acid directly regulates PINOID-dependent phosphorylation and activation of the PIN-FORMED2 auxin efflux transporter in response to salt stress. *Plant Cell* **31**, 250-271 (2019).
32. K. L. Ung *et al.*, Structures and mechanism of the plant PIN-FORMED auxin transporter. *Nature* **609**, 605-610 (2022).
33. M. Michniewicz *et al.*, Antagonistic regulation of PIN phosphorylation by PP2A and PINOID directs auxin flux. *Cell* **130**, 1044-1056 (2007).
34. J. r. Kleine-Vehn *et al.*, PIN auxin efflux carrier polarity is regulated by PINOID kinase-mediated recruitment into GNOM-independent trafficking in *Arabidopsis*. *Plant Cell* **21**, 3839-3849 (2009).
35. P. Dhonukshe *et al.*, Plasma membrane-bound AGC3 kinases phosphorylate PIN auxin carriers at TPRXS (N/S) motifs to direct apical PIN recycling. *Development* **137**, 3245-3255 (2010).
36. G. J. Mitchison, S. Brenner, A model for vein formation in higher plants. *Proc. R. Soc. Lond., Ser. B: Biol. Sci.* **207**, 79-109 (1980).
37. G. J. Mitchison *et al.*, The polar transport of auxin and vein patterns in plants. *Philos. Trans. R. Soc. Lond., Ser. B: Biol. Sci.* **295**, 461-471 (1981).
38. A.-G. Rolland-Lagan, P. Prusinkiewicz, Reviewing models of auxin canalization in the context of leaf vein pattern formation in Arabidopsis. *Plant J.* **44**, 854-865 (2005).
39. S. Stoma *et al.*, Flux-based transport enhancement as a plausible unifying mechanism for auxin transport in meristem development. *PLoS Comp. Biol.* **4**, e1000207 (2008).
40. D. Kierzkowski *et al.*, A growth-based framework for leaf shape development and diversity. *Cell* **177**, 1405-1418. e1417 (2019).
41. E. M. Bayer *et al.*, Integration of transport-based models for phyllotaxis and midvein formation. *Genes Dev.* **23**, 373-384 (2009).
42. H. Jönsson, M. G. Heisler, B. E. Shapiro, E. M. Meyerowitz, E. Mjolsness, An auxin-driven polarized transport model for phyllotaxis. *Proc. Natl. Acad. Sci. U. S. A.* **103**, 1633-1638 (2006).
43. F. G. Feugier, A. Mochizuki, Y. Iwasa, Self-organization of the vascular system in plant leaves: Inter-dependent dynamics of auxin flux and carrier proteins. *J. Theor. Biol.* **236**, 366-375 (2005).
44. F. G. Feugier, Y. Iwasa, How canalization can make loops: A new model of reticulated leaf vascular pattern formation. *J. Theor. Biol.* **243**, 235-244 (2006).
45. K. Abley, S. Sauret-Güeto, A. F. M. Marée, E. Coen, Formation of polarity convergences underlying shoot outgrowths. *eLife* **5**, e18165 (2016).
46. W. Ma, W. S. Noble, T. L. Bailey, Motif-based analysis of large nucleotide data sets using MEME-ChIP. *Nat. Protoc.* **9**, 1428-1450 (2014).
47. A. Maugarny-Calès *et al.*, Dissecting the pathways coordinating patterning and growth by plant boundary domains. *PLoS Genet.* **15**, e1007913 (2019).
48. C. Segonzac *et al.*, The shoot apical meristem regulatory peptide CLV3 does not activate innate immunity. *Plant Cell* **24**, 3186-3192 (2012).

49. Y. Cheng, G. Qin, X. Dai, Y. Zhao, *NPY* genes and AGC kinases define two key steps in auxin-mediated organogenesis in *Arabidopsis*. *Proc. Natl. Acad. Sci. U. S. A.* **105**, 21017-21022 (2008).
50. T. Blein *et al.*, A conserved molecular framework for compound leaf development. *Science* **322**, 1835-1839 (2008).
